# Aggregation‐Induced Emission Molecular Design for Mitigating Non‐Radiative Energy Loss in Organic Solar Cells

**DOI:** 10.1002/adma.202519588

**Published:** 2026-01-20

**Authors:** Yingze Zhang, Rongkun Zhou, Mingjie Rong, Xianghao Zeng, Ho Ming Ng, Ruijie Ma, Joshua Yuk Lin Lai, Chao Li, Shuwei Qiu, Heng Liu, Hao Xia, Lei Zhu, Guangye Zhang, Xinhui Lu, Zilong Zheng, Jun Liu, He Yan, Sai Ho Pun, Gang Li

**Affiliations:** ^1^ Department of Chemistry Guangdong‐Hong Kong‐Macao Joint Laboratory of Optoelectronic and Magnetic Functional Materials Energy Institute and Hong Kong Branch of Chinese National Engineering Research Center for Tissue Restoration & Reconstruction Hong Kong University of Science and Technology Hong Kong China; ^2^ Changchun Institute of Applied Chemistry Chinese Academy of Sciences Changchun China; ^3^ Faculty of Materials and Manufacturing Beijing University of Technology Beijing China; ^4^ Guangdong‐Hong Kong Joint Laboratory For Carbon Neutrality Jiangmen Laboratory of Carbon Science and Technology Jiangmen Guangdong China; ^5^ Department of Electrical and Electronic Engineering Research Institute for Smart Energy (RISE) Photonic Research Institute (PRI) The Hong Kong Polytechnic University Hong Kong China; ^6^ College of New Materials and New Energies Shenzhen Technology University Shenzhen China; ^7^ Department of Physics The Chinese University of Hong Kong Hong Kong China; ^8^ School of Chemistry and Chemical Engineering Frontiers Science Center For Transformative Molecules Shanghai Key Lab of Electrical Insulation & Thermal Aging Shanghai Jiao Tong University Shanghai China

**Keywords:** aggregation‐induced emission, electron acceptor, non‐radiative energy loss, organic solar cells

## Abstract

Non‐radiative energy loss remains a critical bottleneck limiting the open‐circuit voltage (*V*_OC_) and efficiency of organic solar cells (OSCs). Here, we introduce a molecular design strategy that leverages aggregation‐induced emission (AIE) to suppress aggregation‐caused quenching and enhance solid‐state photoluminescence quantum yield (PLQY), thereby mitigating non‐radiative recombination. A prototypical AIE motif, tetraphenylethylene, was incorporated into the terminal group of a Y‐series non‐fullerene acceptor to yield dTPE, which exhibits distinct AIE characteristics not previously observed in high‐performance Y‐series acceptors. Photoluminescence studies reveal that dTPE achieves a threefold enhancement in PLQY compared to L8BO‐C4 in the film, leading to an electroluminescence external quantum efficiency more than an order of magnitude higher than that of D18:L8BO‐C4. Consequently, the binary D18:dTPE device achieves a remarkably low non‐radiative recombination loss of 0.130 eV. When incorporated as a guest into D18:L8BO‐C4 blends, dTPE enables a non‐radiative voltage loss of only 0.190 eV and an unprecedented *V*_OC_ of 0.93 V, yielding an efficiency of 20.5%. To our knowledge, this represents the highest *V*_OC_ reported for OSCs with efficiencies above 20%. This work establishes AIE molecular design as an effective pathway to overcome intrinsic limitations of Y‐series acceptors and provides guiding principles for mitigating non‐radiative energy loss in next‐generation OSCs.

## Graphical abstract

Aggregation‐induced emission strategy is first used to overcome aggregation‐caused quenching in Y‐series acceptors. By integrating tetraphenylethylene, the dTPE exhibits three‐fold PLQY enhancement and significantly suppressed non‐radiative energy loss. An ultra‐low *E*_loss_ of 0.130 eV and a record *V*_OC_ of 0.93 V are achieved, resulting in a PCE over 20.5%. This work establishes AIE design as a guiding principle for high‐performance organic photovoltaics.

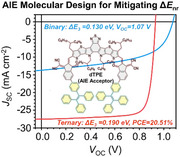

## 1 Introduction

**FIGURE 1:**
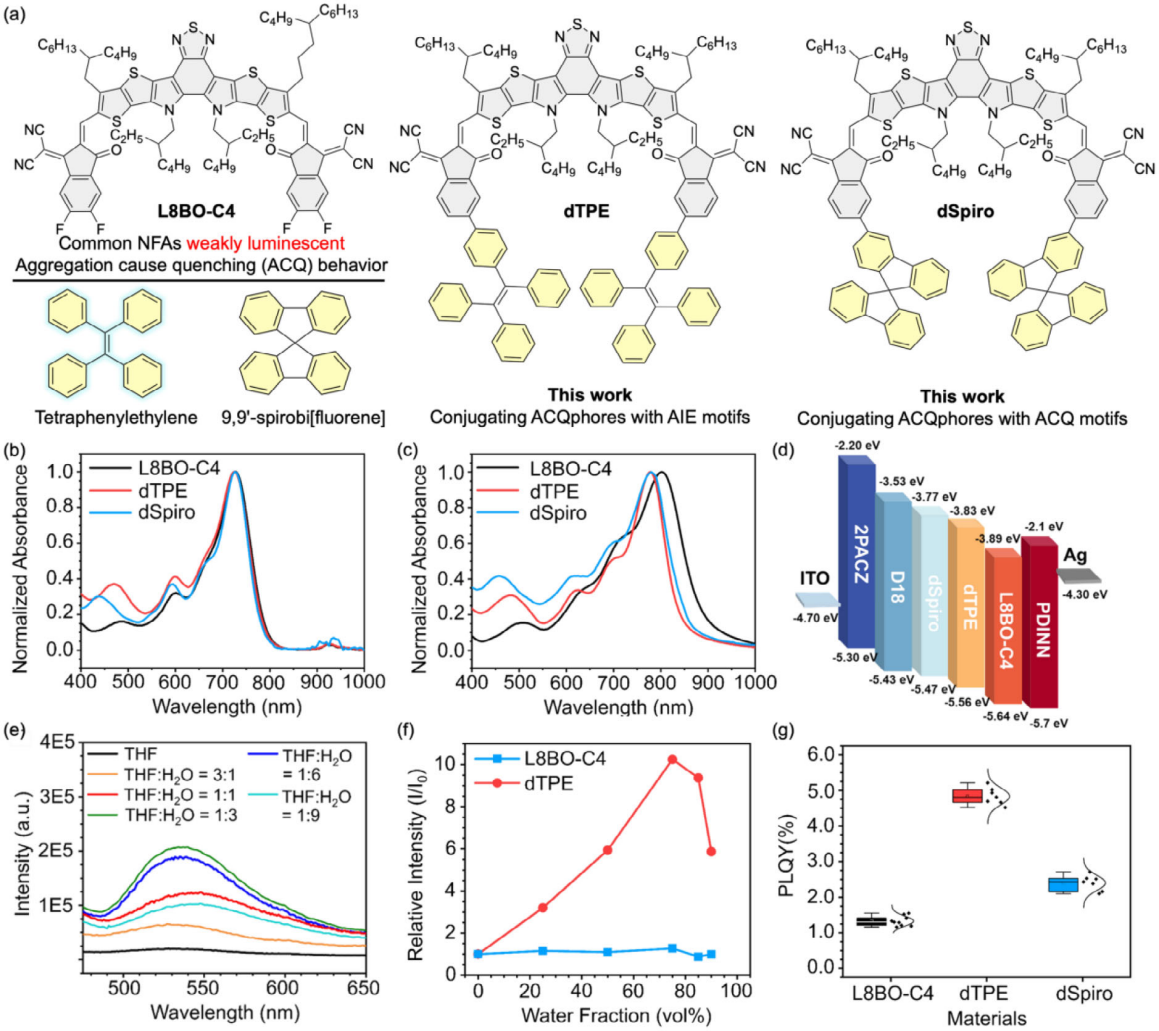
(a) Molecular design and chemical structures of L8BO‐C4, tetraphenylethylene, 9,9′‐spirobi[fluorene], dTPE, and dSpiro. Normalized absorption spectra of these materials (b) in solutions and (c) in films. (d) Energy level diagram of the materials in this work. (e) PL spectra of dTPE in the mixture of tetrahydrofuran (THF) and water. (f) Plots of the relative emission intensity of L8BO‐C4 and dTPE versus water fraction. (g) PLQY of L8BO‐C4, dTPE, and dSpiro neat films.

Organic solar cells (OSCs) have made remarkable progress in the past decade, with power conversion efficiencies (PCEs) now exceeding 20% [1, 2, 3, 4, 5, 6, 7, 8, 9, 10, 11, 12]. Nevertheless, further advancement toward the efficiency range of perovskite and silicon photovoltaics is hampered by large non‐radiative energy losses (Δ*E*_nr_), which severely suppress the open‐circuit voltage (*V*_OC_) and limit device performance [13, 14, 15, 16, 17, 18, 19, 20]. One of the fundamental challenges lies in the molecular design of state‐of‐the‐art Y‐series non‐fullerene acceptors (NFAs) [21, 22, 23, 24, 25, 26, 27]. Their rigid and planar donor–acceptor backbones favor charge transfer but simultaneously suppress radiative decay. As a result, nearly all high‐performing NFAs are weakly luminescent in the solid state (Figure 1a, left), constrained by aggregation‐caused quenching (ACQ) behavior that enforces persistent Δ*E*_nr_ losses [28, 29]. Overcoming this photophysical bottleneck is widely regarded as a critical frontier for breaking the efficiency plateau of OSCs [30, 31, 32, 33].

One widely explored approach to reducing Δ*E*_nr_ has been the incorporation of ternary guest components, which often yield improved photoluminescence quantum yield (PLQY) and electroluminescence quantum efficiency (EQE_EL_) [34, 35, 36]. A seminal study by Gao and co‐workers established guiding principles for the design of guest materials [37]. The guest‐based binary blend should exhibit a higher EQE_EL_ than the host system, thereby facilitating thermal population of radiative states and suppressing non‐radiative decay. Consistent with these principles, many successful ternary systems employ luminescent guest acceptors. For example, Sun and co‐workers introduced a triphenylamine‐functionalized NFA (Z‐Tri) that suppressed non‐radiative losses and boosted EQE_EL_ by more than an order of magnitude compared with PM6:L8‐BO [38]. We demonstrated oligomers with D‐A‐D‐A‐D and A‐D‐A‐D‐A configuration(named 5BDTBDD and 5BDDBDT) give higher EQE_EL_ of up to 0.05%, enable high‐performance ternary OSCs with low energy loss [34]. These studies highlight the importance of enhancing both PLQY and EQE_EL_ in acceptors as a direct route to suppress Δ*E*_nr_. However, the intrinsic ACQ character of Y‐series cores remains unchanged, placing an inherent ceiling on such strategies.

A transformative solution is to invert this paradigm by harnessing aggregation‐induced emission (AIE). In contrast to ACQ systems, AIE luminogens are non‐emissive in dilute solution but become highly luminescent upon aggregation, making them ideally suited for the condensed morphology of OSC active layers [39, 40]. Embedding AIE motifs into NFAs thus offers a conceptually powerful and previously unexplored strategy to couple favorable electronic properties with enhanced solid‐state luminescence. In this study, we present a new design strategy that integrates AIE‐active motifs directly into Y‐series acceptors. By incorporating tetraphenylethylene (TPE) into the terminal groups, we developed the acceptor dTPE (Figure 1a, middle), along with a control analogue dSpiro based on spirobifluorene (Figure 1a, right), which features a rigid aromatic group of similar size but lacks AIE character. Strikingly, dTPE films exhibit pronounced AIE behavior with a PLQY three times higher than L8BO‐C4, a feature not previously observed in high‐performance Y‐series acceptors, whereas dSpiro retains conventional ACQ character. As a result, the guest binary of D18:dTPE achieved an EQE_EL_ more than an order of magnitude compared with the host binary of D18:L8BO‐C4 and a remarkably low Δ*E*_nr_ of 0.130 eV. When incorporated dTPE as a guest into D18:L8‐BO‐C4 blends, the enhanced PLQY translated into a remarkably low non‐radiative voltage loss of 0.190 eV and an unprecedented *V*_OC_ of 0.93 V, yielding a PCE of 20.5%. To our knowledge, this represents the highest *V*_OC_ reported for OSCs with efficiencies above 20%.

More than achieving another efficiency milestone, this work establishes a new conceptual framework for molecular design in OSCs. By transforming ACQ into AIE, we demonstrate a generalizable approach to mitigating non‐radiative recombination losses at the molecular level. These findings position AIE not as a niche phenomenon but as a guiding design principle for next‐generation organic semiconductors, pointing the way toward OSCs with both high efficiency and intrinsically low energy loss.

## 2 Results and Discussion

The synthetic routes for dTPE and dSpiro are straightforward, requiring only an additional Suzuki coupling step to obtain the IC‐TPE and IC‐Spiro terminal groups from commercially available bromine precursors. Detailed synthesis procedures and characterization, including NMR and mass spectra, are provided in Schemes S1 and S2 and Figures S19–S28. Both molecules show good solubility in common processing solvents such as chloroform and chlorobenzene, ensuring their suitability for device fabrication.

The optical absorption spectra of L8BO‐C4, dTPE, and dSpiro in solution and thin film are compared in Figure 1b,c, with key parameters summarized in Table S3. In chloroform solution, all three molecules display nearly identical absorption maxima around 727–728 nm, with extinction coefficients in the range of 1.2–1.8 × 10^5^ L·mol^−^^1^·cm^−^^1^, indicating that incorporation of TPE or Spiro units exerts minimal influence on the intrinsic electronic transitions of the molecular backbone. By contrast, notable differences arise in the solid state. Upon film formation, L8BO‐C4 exhibits a pronounced redshift of 74 nm, whereas dTPE and dSpiro show more moderate shifts of 52 and 53 nm, respectively, reflecting their distinct aggregation behaviors. The corresponding optical bandgaps (*E*_g_) were determined to be 1.40 eV for L8BO‐C4, 1.46 eV for dTPE, and 1.44 eV for dSpiro. Charge transport was evaluated using electron‐only devices via the space‐charge limited current (SCLC) method (Figure S8). The electron mobilities (*µ*_e_) decrease in the order L8BO‐C4 > dTPE > dSpiro, with values of 9.67 × 10^−^^4^, 5.28 × 10^−^^4^, and 2.87 × 10^−^^4^ cm^2^ V^−^^1^ s^−^^1^, respectively. Cyclic voltammetry (Figure S3) revealed slight upshifts in both highest occupied molecular orbital (HOMO) and lowest unoccupied molecular orbital (LUMO) levels for dTPE (−5.56/−3.83 eV) and dSpiro (−5.47/−3.77 eV) compared to L8BO‐C4 (−5.64/−3.89 eV), consistent with the weaker electron‐deficient character of the modified terminal groups and intramolecular charge transfer effect. Density functional theory (DFT) calculations further corroborate these observations, showing that dTPE forms a favorable cascade energy alignment with D18 and L8BO‐C4 (Figures 1d; S1 and S2), which can facilitate charge separation and transport in devices. Thermogravimetric analysis (TGA) reveals that dTPE exhibits a higher thermal decomposition temperature (330.1°C) than L8BO‐C4 (322.4°C) (Figure S31a). Furthermore, the glass transition temperatures (*T*_g_) were obtained from the temperature‐dependent UV–vis absorption spectra. As illustrated in Figure S31b, the *T*_g_ of dTPE, L8BO‐C4, and L8BO‐C4:dTPE films are calculated to 125.3°C, 121.5°C, and 122.3°C, respectively. These results demonstrate that dTPE exhibits superior thermal properties compared to L8BO‐C4.

Fluorescence spectroscopy was used to probe the luminescence characteristics of dTPE in comparison with conventional Y‐series acceptors. In dilute tetrahydrofuran solution, both dTPE and L8BO‐C4 exhibited weak emission in the 500–650 nm region (Figures 1e; S4). Upon gradual addition of water as a poor solvent to induce aggregation, a striking divergence emerged. dTPE developed a pronounced emission peak centered at ∼530 nm, and its photoluminescence intensity increased tenfold at a THF/water ratio of 1:3. By contrast, the emission of L8BO‐C4 remained essentially unchanged under identical conditions (Figure 1f), confirming the presence of AIE behavior in dTPE. Additional excitation at 530 nm further underscored these differences (Figure S5). Upon addition of water, L8BO‐C4 displayed severe ACQ, with its emission intensity dropping to only 5.3% of the original. In contrast, dTPE retained 72.9% of its fluorescence intensity, which remained stable with higher water fractions. This resilience can be attributed to the sterically hindered TPE unit, which suppresses detrimental π–π stacking and alleviates ACQ. The impact of this design is directly reflected in the solid‐state PLQYs. As summarized in Figures 1g and S6 and S7, the film PLQYs of L8BO‐C4, dTPE, and dSpiro were 1.33 ± 0.13%, 4.69 ± 0.19%, and 2.43 ± 0.12%, respectively. The combination of AIE activation and ACQ suppression thus enables dTPE to achieve a more than threefold enhancement in solid‐state luminescence relative to L8BO‐C4. Such an improvement is expected to directly mitigate non‐radiative recombination losses in organic solar cells, as the Δ*E*_nr_ is quantitatively linked to the EQE_EL_ through the relation Δ*E*_nr_ = −kT ln(EQE_EL_). Since EQE_EL_ in turn depends strongly on the PLQY of the active layer, the solid‐state luminescence of dTPE provides a direct pathway toward reducing Δ*E*_nr_ at the molecular level.

**FIGURE 2:**
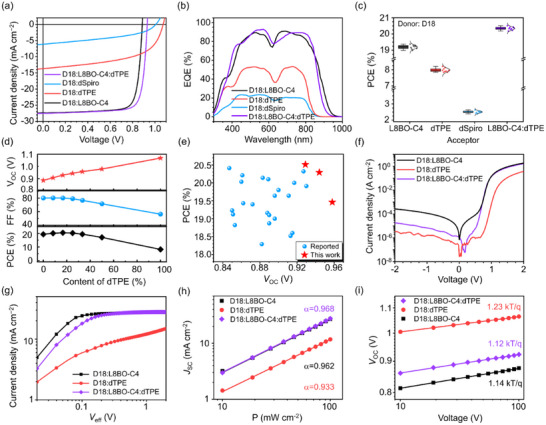
(a) Characteristic *J*–*V* curves of the optimized binary and ternary devices under simulated AM 1.5G irradiation (100 mW cm^−2^). (b) The corresponding EQE spectra and integrated *J*_SC_ values of binary and ternary devices. (c) Efficiency‐distributed box plots of the binary and ternary devices derived from 12 independent devices. (d) The dependence curves of *V*_OC_, *FF*, and PCE of the ternary devices on the dTPE content. (e) Efficiency‐distributed box plots of the binary and ternary devices based on D18:acceptor active layer. (e) Plots of the PCE versus *V*_OC_ of the devices reported in 2025. (f) The *J*–*V* curves of binary and ternary devices in dark conditions. (g) Photocurrent density versus effective bias characteristics, (h) *J*_SC_ versus *P*, and (i) *V*_OC_ versus *P* characteristics of the binary and ternary devices. In this figure, the D18:L8BO‐C4:dTPE ternary device was prepared with a blend ratio of 1:1:0.2.

Organic solar cell devices were fabricated in a conventional architecture of ITO/2PACz/D18:A1:(A2)/PDINN/Ag, with details provided in the supporting information. The current density–voltage (*J*–*V*) characteristics and photovoltaic parameters are shown in Figures 2a, S9, and summarized in Table 1. Among the binary devices, D18:dTPE yielded a moderate PCE of 8.21%, with a short‐circuit current density (*J*_SC_) of 13.69 mA cm^−^^2^, a fill factor (*FF*) of 56.4%, and an outstanding *V*_OC_ of 1.069 V. By contrast, D18:dSpiro delivered a much lower PCE of 2.63% with inferior *J*_SC_ (6.17 mA cm^−^^2^), *FF* (42.1%), and *V*_OC_ (1.005 V), which can be attributed to its elevated HOMO level and unfavorable exciton dissociation. These results indicate that while the sterically hindered TPE group compromises charge transport in neat blends, it simultaneously endows dTPE with an exceptionally high *V*_OC_, underscoring its huge potential as a guest acceptor in high‐performance systems. Therefore, dTPE was introduced into the benchmark D18:L8BO‐C4 system. As the dTPE fraction increased, the ternary devices displayed a systematic rise in *V*_OC_ from 0.886 V to as high as 1.069 V (Figure 1d; Table S1), while the *FF* remained stable at low guest contents but decreased at higher loadings. Consequently, the PCE exhibited a volcano‐like dependence on composition. The optimized ternary blend (1:1:0.2) achieved a champion PCE of 20.51%, surpassing the 19.37% of the D18:L8BO‐C4 binary device. External quantum efficiency (EQE) spectra (Figure 2b) confirmed the origin of the performance enhancement. The ternary device exhibited a slightly blue‐shifted spectrum and a modest increase in the short‐wavelength region, resulting in a *J*_SC_ of 27.51 mA cm^−^^2^, consistent with the integrated photocurrent (discrepancy < 5%), validating the accuracy of the *J*–*V* measurements. Reproducibility was verified through statistical analysis (Figure 2c). Notably, a blend ratio of 1:0.9:0.3 also delivered a high PCE of 20.29% while maintaining an elevated *V*_OC_ of 0.944 V, illustrating the robustness of the design. Importantly, the record‐high *V*_OC_ of 0.944 V for the optimized ternary device sets a new benchmark for organic solar cells with PCEs exceeding 20% (Figure 2e; Table S7).

**TABLE 1:** Summary of the detailed photovoltaic parameters of the binary and ternary OSCs.

Active layer	*V*_OC_ [V]	*J_SC_* [mA cm^−2^]	*J*_cal_ [mA cm^−2^]	*FF* [%]	PCE(PCE^a^) [%]
D18: L8BO‐C4	0.886 (0.885 ± 0.002)	27.33 (27.23 ± 0.14)	26.34	80.2 (79.8 ± 0.6)	19.37 (19.17 ± 0.20)
D18: dTPE	1.069 (1.067 ± 0.003)	13.69 (13.49 ± 0.21)	13.38	56.4 (56.0 ± 0.5)	8.21 (7.98 ± 0.23)
D18: dSpiro	1.005 (1.003 ± 0.002)	6.17 (6.03 ± 0.15)	5.83	42.1 (41.9 ± 0.4)	2.63 (2.51 ± 0.12)
D18: L8BO‐C4: dTPE (1:1:0.2)	0.929 (0.926 ± 0.003)	27.51 (27.34 ± 0.19)	26.41	80.2 (79.6 ± 0.7)	20.51 (20.34 ± 0.17)

^a^ Average values obtained from 12 devices.

To gain further insights into exciton dissociation and charge collection, the photocurrent density (*J*_ph_) as a function of the effective voltage (*V*_eff_) was analyzed. As shown in Figure 2g, the charge collection efficiencies (*η*) of the D18:L8BO‐C4, D18:dTPE, and D18:L8BO‐C4:dTPE devices were determined to be 99.0%, 82.5%, and 99.3%, respectively. The relatively low *η* of the D18:dTPE device reflects its limited charge transport arising from the less favorable active layer morphology. In contrast, the ternary device achieves a slightly higher *η* than the binary counterpart, demonstrating that the incorporation of dTPE facilitates exciton dissociation and charge collection, in good agreement with its improved *J*_SC_. To investigate recombination behavior, dark *J*–*V* and light‐intensity–dependent measurements were carried out. In OSCs, dark current originates primarily from carrier recombination within the active layer or at the interfaces [41, 42, 43]. As shown in Figure 2f, the dark current densities (*J*_d_) at −2 V bias for the D18:L8BO‐C4, D18:dTPE, and D18:L8BO‐C4:dTPE devices were 2.67 × 10^−^^4^, 3.26 × 10^−^^6^, and 1.89 × 10^−^^5^ A cm^−^^2^, respectively. The ternary device therefore exhibits a *J*_d_ one order of magnitude lower than that of the binary device, highlighting the suppression of non‐radiative recombination and the consequent reduction in energy loss. The recombination kinetics were further analyzed by monitoring the dependence of *J*_SC_ and *V*_OC_ on light intensity (*P*) (Figure S12). The *J*_SC_–*P* relationship follows *J*_SC_ ∝ *P* ^α^, where α characterizes the degree of bimolecular recombination [44, 45, 46]. As shown in Figure 2h, the extracted α values for D18:L8BO‐C4, D18:dTPE, and D18:L8BO‐C4:dTPE devices are 0.962, 0.933, and 0.968, respectively. The α value of the ternary device is closer to unity than that of the binary device, indicating its reduced bimolecular recombination. For *V*_OC_–*P* analysis, the slope of *V*_OC_ versus ln(*P*) indicates the extent of trap‐assisted recombination [47, 48, 49]. As presented in Figure 2i, the slopes of the D18:L8BO‐C4, D18:dTPE, and D18:L8BO‐C4:dTPE devices are 1.14, 1.23, and 1.12 kT/q, respectively, confirming the suppression of trap‐assisted recombination in the ternary system. Charge transport characteristics were evaluated using SCLC method [50, 51]. As presented in Figures S10 and S11, the *µ*_e_ and *µ*_h_ of the D18:dTPE device are 3.14 × 10^−^^4^ and 7.64 × 10^−^^4^ cm^2^ V^−^^1^ s^−^^1^, respectively. For the D18:L8BO‐C4 binary film, *µ*_e_ and *µ*_h_ are 4.66 × 10^−^^4^ and 6.49 × 10^−^^4^ cm^2^ V^−^^1^ s^−^^1^, with a *µ*_h_/*µ*_e_ ratio of 1.39. Incorporating dTPE increases both carrier mobilities to 5.67 × 10^−^^4^ (*µ*_e_) and 7.55 × 10^−^^4^ cm^2^ V^−^^1^ s^−^^1^ (*µ*_h_), while reducing the *µ*_h_/*µ*_e_ ratio to 1.30. The detailed parameters are summarized in Table S2. These results suggest a more balanced and efficient charge transport in the ternary blend. Taken together, the higher exciton dissociation efficiency, balanced carrier mobilities, and suppressed recombination losses in the D18:L8BO‐C4:dTPE device account for its superior photovoltaic performance compared to the binary control.

**TABLE 2:** Detailed energy loss analysis of the optimized ternary and binary devices.

Active layer	*E*_g_ ^a^ [eV]	q*V*_OC_ [eV]	*E_loss_* [eV]	q*V_OC_^SQ^* ^b^ [eV]	Δ*E*_1_ [eV]	Δ*E*_2_ [eV]	Δ*E*_nr_ ^c^ [eV]	*EQE*_EL_	*E_U_* ^d^ [meV]
D18: L8‐BOC4	1.425	0.882	0.543	1.164	0.261	0.066	0.219	2.29 × 10^−4^	25.06
D18:dTPE	1.516	1.064	0.452	1.251	0.265	0.065	0.130	5.68 × 10^−3^	26.05
D18:dSpiro	1.522	1.005	0.517	1.259	0.263	0.071	0.194	3.53 × 10^−4^	28.07
D18:L8‐BOC4:dTPE	1.445	0.929	0.516	1.181	0.264	0.066	0.190	5.39 × 10^−4^	23.22

^a^ *E*_g_ values are deduced from the intercrossing of the normalized absorption and emission spectra.

^b^ q*V*_OC_^SQ^ is the maximum *V*_OC_ by the SQ limit.

^c^ Δ*E_nr_* is determined from the EQE_EL_.

^d^ *E*_U_ is commonly‐used to evaluate the energetic disorder degree in organic semiconductor device, which can be calculated from the EQE band tails [52, 53].

**FIGURE 3:**
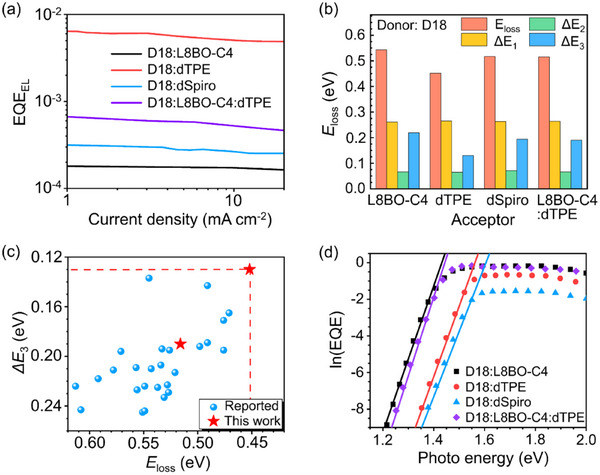
(a) EQE_EL_ as a function of injection current and the (b) statistical diagram of energy loss for the binary and ternary devices. (c) Plots of the Δ*E*_3_ versus *E*_loss_ of the OSCs reported in the literatures. (d) Extraction of Urbach energy from ln(EQE) in the long‐wavelength edge.

To elucidate the origin of the high *V*_OC_ in TPE‐based devices, we conducted a comprehensive energy loss (*E*_loss_) analysis (Figure S13 and Table 2). As shown in Figure 3a, the EQE_EL_ values for D18:L8BO‐C4, D18:dTPE, D18:dSpiro, and D18:L8BO‐C4:dTPE are 2.29 × 10^−^^4^, 5.68 × 10^−^^3^, 3.53 × 10^−^^4^, and 5.39 × 10^−^^4^, respectively. Compared to L8BO‐C4, dSpiro benefits from its bulky end group, which weakens molecular packing and suppresses ACQ, thereby slightly enhancing its EQE_EL_. Remarkably, despite sharing a sterically bulky end group motif with dSpiro, dTPE delivers an EQE_EL_ as high as 0.57%, more than an order of magnitude higher than both dSpiro and L8BO‐C4. This EQE_EL_ value is among the best reported for state‐of‐the‐art OSC acceptors (Figure S14), and originates from the intrinsic AIE behavior of dTPE, which promotes strong solid‐state luminescence. As a result, the D18:dTPE binary device achieves an exceptionally low *E*_loss_ of 0.452 eV, significantly lower than that of D18:L8BO‐C4 (0.543 eV) and D18:dSpiro (0.517 eV). Incorporation of 20 wt.% dTPE into the D18:L8BO‐C4 host further reduces *E*_loss_ from 0.543 to 0.516 eV in the ternary device. Figure 3b highlights that suppression of Δ*E*_nr_ is the dominant factor underlying this reduced *E*_loss_. It is widely recognized that minimizing Δ*E*_nr_ is one of the most critical yet challenging tasks in pushing OSC performance toward its theoretical limit, and only a few molecular design strategies have proven effective [8, 54, 55, 56, 57]. Here, dTPE achieves a Δ*E*_nr_ as low as 0.130 eV, representing the lowest non‐radiative loss reported to date in OSCs (Figure 3c; Table S6). The ternary device also benefits, with Δ*E*_nr_ reduced to 0.190 eV compared to 0.219 eV for the binary control. These results provide compelling evidence that the AIE molecular design is an effective pathway to enhance luminescence in non‐fullerene acceptors and simultaneously suppress non‐radiative recombination losses in OSCs. We further evaluated energetic disorder through the Urbach energy (*E*_U_), extracted from Fourier‐transform photocurrent spectroscopy (FTPS). As shown in Figure 3d, the optimized ternary blend exhibits a *E*_U_ of 23.22 meV, significantly lower than that of D18:L8BO‐C4 (25.06 meV), D18:dTPE (26.05 meV), and D18:dSpiro (28.07 meV). Electrochemical impedance spectroscopy was also performed to investigate the shunt resistance (*R*_sh_). As shown in Figure S34, the *R*_sh_ value of D18:L8BO‐C4:dTPE‐based device (17.3 kΩ) is higher than that of D18:L8BO‐C4‐based device (15.1 kΩ). The reduced *E*_U_ and enhanced *R*_sh_ indicates that the incorporation of dTPE promotes more ordered π–π stacking in the blend film, further contributing to suppressed energetic disorder and improved *V*_OC_.

**FIGURE 4:**
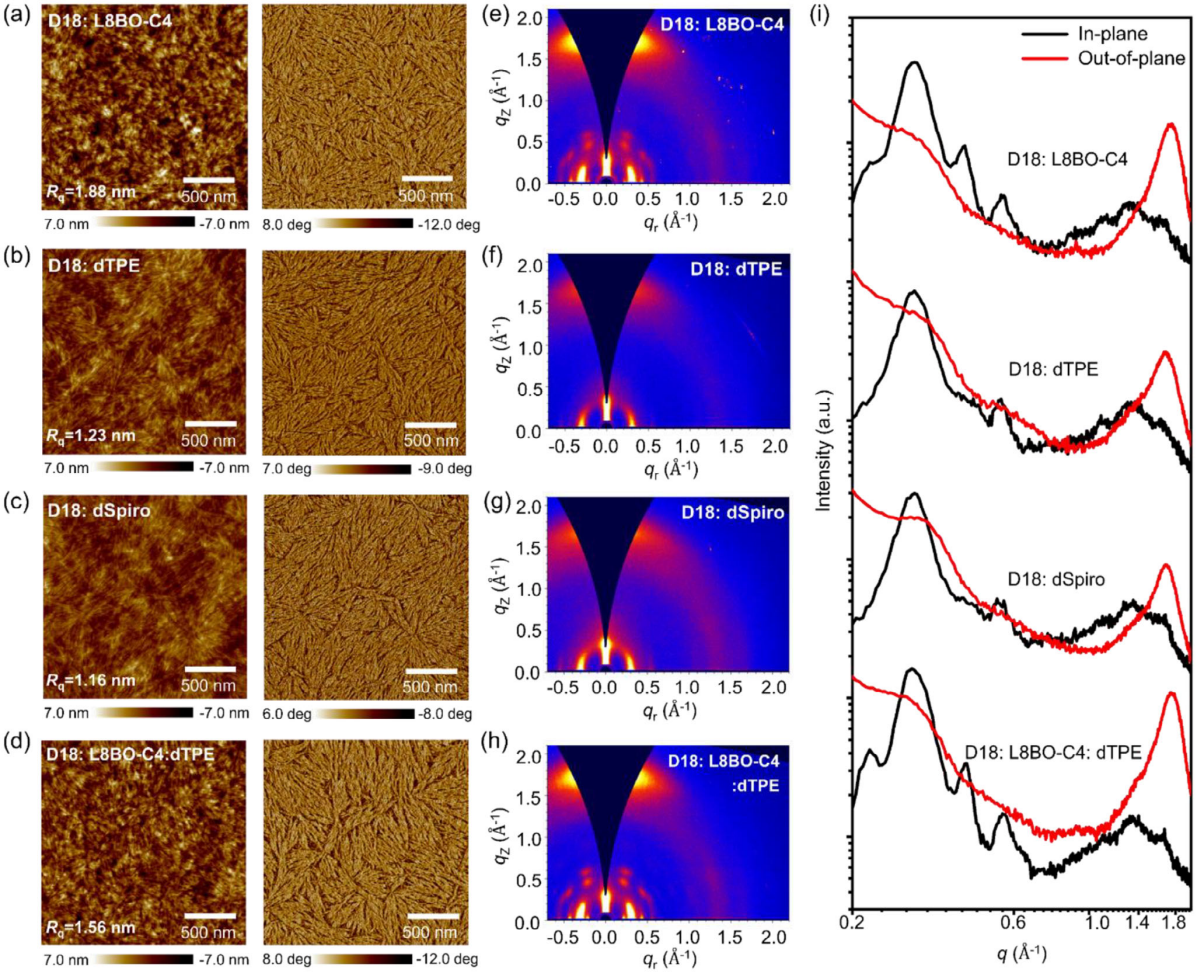
The AFM height images and phase images of (a) D18: L8BO‐C4, (b) D18: dTPE, (c) D18: dSpiro, and (d) D18: L8BO‐C4: dTPE blended films. The 2D GIWAXS profiles of (e) D18: L8BO‐C4, (f) D18: dTPE, (g) D18: dSpiro, (h) D18: L8BO‐C4: dTPE blended films, and i) the corresponding 1D line‐cut curves along OOP and IP directions of the binary and ternary blended films.

Atomic force microscopy (AFM) was employed to probe the surface morphology of the active layers. As shown in Figure 4a–d, all four blend films exhibit well‐defined fibrillar features. The binary D18:L8BO‐C4 film displays a root‐mean‐square roughness (*R*_q_) of 1.88 nm, while both the D18:dTPE (*R*_q_ = 1.23 nm) and D18:dSpiro (*R*_q_ = 1.26 nm) films exhibit lower roughness values, which can be attributed to their relatively weaker crystallinity. Interestingly, incorporation of 20 wt.% dTPE into the D18:L8BO‐C4 host results in a ternary film with a reduced roughness of 1.56 nm, accompanied by enlarged fiber dimensions and a more favorable fibrous interpenetrating network. This morphology is consistent with the enhanced charge transport observed in the ternary devices, supporting more efficient exciton dissociation and carrier collection. To gain deeper insight into molecular packing, grazing‐incidence wide‐angle X‐ray scattering (GIWAXS) measurements were carried out on both neat and blend films (Figures 4e–i; S15). The three acceptor neat films all adopt a preferential face‐on orientation, with distinct (010) diffraction peaks in the out‐of‐plane (OOP) direction (Table S4). The *q*_z_ positions of these peaks correspond to π–π stacking distances of 3.56 Å (L8BO‐C4), 3.86 Å (dTPE), and 3.89 Å (dSpiro), confirming that the bulky end groups of dTPE and dSpiro relax their molecular packing. Coherence length (CL) analysis further indicates weaker crystallinity in dTPE (31 Å) and dSpiro (17 Å) compared with L8BO‐C4 (36 Å). The blend films exhibit similar face‐on orientation, but with notable differences in crystallinity (Table S5). The D18:L8BO‐C4 film shows an out‐of‐plane (010) coherence length of 26 Å, which decreases to 23 and 20 Å in the D18:dTPE and D18:dSpiro films, respectively, consistent with their less crystalline nature. Unexpectedly, however, the D18:L8BO‐C4:dTPE ternary blend shows π–π stacking distance reduced from 3.62 to 3.58 Å and the CL increased from 26 to 29 Å. The incorporation of dTPE promotes a more ordered stacking at D18/L8BO‐C4 interface, resulting in shorter π‐π stacking distances. This observation is intriguing, as dTPE itself displays weaker crystallinity and looser packing in its neat film.

**FIGURE 5:**
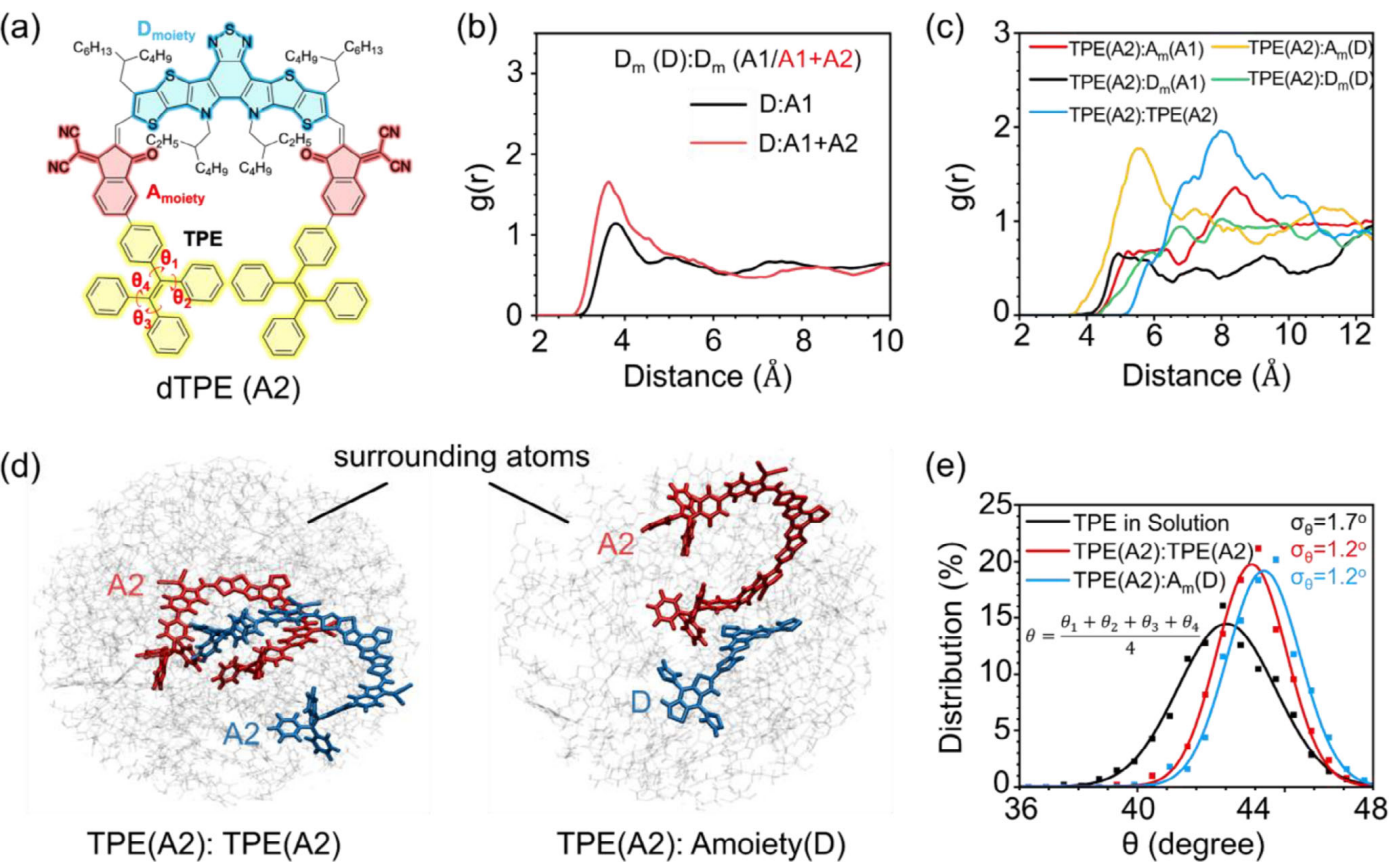
MD simulations revealing the restricted intramolecular rotation of dTPE in ternary blend. (a) The donor moiety (*D*_moiety_), acceptor moiety (*A*_moiety_), and TPE moiety of dTPE (denoted as A2). (b) g(r) between the *D*_moiety_ of donor and the *D*_moiety_ of acceptor in binary (black lines) and ternary (red lines) blends. The *D*_moiety_ of acceptor in binary blend and ternary blend are denoted as D_m_(A1) and *D*_m_(A1+A2), respectively. (c) g(r) between the TPE moiety of A2 and the other moieties. (d) Representative snapshots of the TPE(A2):TPE(A2) and TPE(A2):*A*_m_(D) stacking configurations extracted from the MD simulated ternary blend. (e) Gaussian fitted distributions of the average intramolecular torsion angles (θ) of the TPE moieties in solution, TPE(A2):TPE(A2) stacking, and TPE(A2):*A*_m_(D) stacking calculated from 500 frames of the MD trajectory. σ_θ_ represents the torsion angle disorder.

To gain further mechanistic understanding of why the ternary blend exhibits improved order despite the bulky TPE group as well as to elucidate the origin of restricted intramolecular rotation (RIR), molecular dynamics (MD) simulations were carried out on both binary (D:A1) and ternary (D:A1:A2) systems. Intermolecular packing was evaluated using the radial distribution function, g(r). As shown in Figures 5b and S18, the g(r) between the donor and acceptor in the ternary blend exhibits a markedly sharper first peak at 3.6 Å compared to the weaker peak at 3.8 Å in the binary blend. This result indicates that the incorporation of dTPE promotes more ordered packing and a shorter π–π stacking distance at the D:A interface, consistent with the GIWAXS analysis. We then examined the local environment of the TPE moieties of dTPE to understand the origin of the RIR effect. The g(r) analysis in Figure 5c reveals two dominant interactions including TPE self‐aggregation (TPE(A2):TPE(A2)) and stacking between TPE and the acceptor moiety of the donor (TPE(A2):A_moiety_(D)). Representative snapshots of these configurations are displayed in Figure 5d. In both cases, adjacent molecular fragments intercalate between the phenyl rings of TPE, creating steric hindrance that restricts intramolecular torsional motion. This restriction is confirmed by a narrower torsion angle distribution (Figure 5e), with the standard deviation (σ_θ_) decreasing from 1.7° in solution to 1.2° in the blend. Such restricted intramolecular rotation serves as the molecular basis for activating aggregation‐induced emission while simultaneously stabilizing intermolecular packing in the ternary system. These results demonstrate that dTPE, as a guest acceptor, can not only reduce the Δ*E*_nr_ of OSCs, but also enhance device performance by optimized blended morphology.

## 3 Conclusion

In summary, we have demonstrated that aggregation‐induced emission molecular design offers a powerful approach to suppressing non‐radiative recombination losses in OSCs. By introducing TPE units into the Y‐series acceptor, dTPE exhibits AIE behavior that suppresses ACQ and enhances film‐state PLQY. This molecular‐level advancement directly translates into a high EQE_EL_ and a remarkable non‐radiative recombination loss as low as 0.130 eV in binary devices, as well as a record *V*_OC_ of 0.93 V with a Δ*E*_nr_ of 0.190 eV and a PCE of 20.5% in ternary blends. Beyond these record metrics, this study highlights a generalizable strategy that bridges the molecular photophysics of AIE with photovoltaic performance optimization. We believe this work not only enriches the design toolbox for high‐efficiency OSCs but also points toward a future where molecular design of luminescent acceptors with enhanced PLQY becomes a central strategy for suppressing non‐radiative losses.

## Conflicts of Interest

The authors declare no conflicts of interest.

## Supporting information




**Supporting File 1**: adma72166‐sup‐0001‐Data.zip.

**Supporting File 2**: adma72166‐sup‐0002‐SuppMat.docx.

## Data Availability

The data that support the findings of this study are available in the supplementary material of this article.

## References

[1] Y. Lin , J. Wang , Z. G. Zhang , et al., “An Electron Acceptor Challenging Fullerenes for Efficient Polymer Solar Cells,” Advanced Materials 27 (2015): 1170–1174. 25580826 10.1002/adma.201404317

[2] J. Yuan , Y. Zhang , L. Zhou , et al., “Single‐Junction Organic Solar Cell with over 15% Efficiency Using Fused‐Ring Acceptor with Electron‐Deficient Core,” Joule 3 (2019): 1140–1151.

[3] Y. Cui , H. Yao , J. Zhang , et al., “Single‐Junction Organic Photovoltaic Cells With Approaching 18% Efficiency,” Advanced Materials 32 (2020): 1908205. 10.1002/adma.201908205 32227399

[4] C. Li , J. Zhou , J. Song , et al., “Non‐Fullerene Acceptors with Branched Side Chains and Improved Molecular Packing to Exceed 18% Efficiency in Organic Solar Cells,” Nature Energy 6 (2021): 605–613.

[5] J. Yi , G. Zhang , H. Yu , and H. Yan , “Advantages, Challenges and Molecular Design of Different Material Types used in Organic Solar Cells,” Nature Reviews Materials 9 (2023): 46–62.

[6] L. Guo , J. Song , J. Deng , et al., “Suppression of Charge Recombination Induced by Solid Additive Assisting Organic Solar Cells With Efficiency Over 20%,” Advanced Materials 37 (2025): 2504396. 10.1002/adma.202504396 40178357

[7] H. Mou , Y. Yin , H. Chen , et al., “Transient Dipole Strategy Boosts Highly Oriented Self‐Assembled Monolayers for Organic Solar Cells Approaching 21% Efficiency,” Journal of the American Chemical Society 147 (2025): 21241–21251. 40476461 10.1021/jacs.5c08124

[8] N. Yang , S. Zhang , Y. Cui , J. Wang , S. Cheng , and J. Hou , “Molecular Design for Low‐Cost Organic Photovoltaic Materials,” Nature Reviews Materials 10 (2025): 404–424.

[9] G. Chen , H. Huang , W. Ma , et al., “Thickness‐Tolerant A1–A2 Polyelectrolyte Cathode Interlayers via Direct Arylation Polycondensation for 20.5% Efficiency Organic Solar Cells,” Advanced Functional Materials (2025): 16196.

[10] J. Fu , H. Li , H. Liu , et al., “Two‐Step Crystallization Modulated through Acenaphthene Enabling 21% Binary Organic Solar Cells and 83.2% Fill Factor,” Nature Energy (2025): 1–1.

[11] C. Xu , J. Yang , S. Gámez‐Valenzuela , et al., “A Bithiophene Imide‐Based Polymer Donor for Alloy‐Like Ternary Organic Solar Cells with Over 20.5% Efficiency and Enhanced Stability,” Energy & Environmental Science 18 (2025): 5913–5925.

[12] C. Li , Y. Cai , P. Hu , et al., “Organic Solar cells with 21% Efficiency Enabled by a Hybrid Interfacial Layer with Dual‐Component Synergy,” Nature Materials 24 (2025): 1626–1634. 40681865 10.1038/s41563-025-02305-8

[13] X.‐K. Chen , D. Qian , Y. Wang , et al., “A Unified Description of Non‐Radiative Voltage Losses in Organic Solar Cells,” Nature Energy 6 (2021): 799–806.

[14] G. Zhang , F. R. Lin , F. Qi , et al., “Renewed Prospects for Organic Photovoltaics,” Chemical Reviews 122 (2022): 14180–14274. 35929847 10.1021/acs.chemrev.1c00955

[15] H. Xiang , F. Sun , X. Zheng , et al., “Tackling Energy Loss in Organic Solar Cells via Volatile Solid Additive Strategy,” Advanced Science 11 (2024): 2401330. 38634564 10.1002/advs.202401330 PMC11220641

[16] J. Zhu , R. Zeng , E. Zhou , et al., “A Refined Bulk P–I–N Structure in All‐Polymer Solar Cells To Achieve 20.1% Efficiency and Improved Stability,” Journal of the American Chemical Society 147 (2025): 24491–24501. 40589082 10.1021/jacs.5c04656

[17] J. Zhang , W. Wei , Z. Luo , et al., “Halogenation‐Engineered Acceptor Enables 20.14% Efficiency in Hydrocarbon‐Solvent Processed OSCs: From Binary Trade‐Offs to Ternary Synergy in Exciton and Energy Loss Management,” Angewandte Chemie International Edition 137 (2025): 202512237. 10.1002/anie.202512237 40888489

[18] J. Zhang , X. Duan , X. Li , et al., “Achieving 20% Efficiency in Binary Organic Solar Cells with Suppressed Non‐Radiative Recombination via Triphenylamine Halides,” Energy & Environmental Science 18 (2025): 5378–5388.

[19] C. Li , J. Song , H. Lai , et al., “Non‐Fullerene Acceptors with High Crystallinity and Photoluminescence Quantum Yield Enable >20% Efficiency Organic Solar Cells,” Nature Materials 24 (2025): 433–443. 39880932 10.1038/s41563-024-02087-5

[20] Q. Jiang , X. Yuan , Y. Li , et al., “A Structurally Simple Polymer Donor Enables High‐Efficiency Organic Solar Cells With Minimal Energy Losses,” Angewandte Chemie International Edition 64 (2025): 202416883. 10.1002/anie.202416883 40074709

[21] C. Duan and L. Ding , “The New Era for Organic Solar Cells: Non‐Fullerene Small Molecular Acceptors,” Science Bulletin 65 (2020): 1231–1233. 36747408 10.1016/j.scib.2020.04.030

[22] S. Li , C.‐Z. Li , M. Shi , and H. Chen , “New Phase for Organic Solar Cell Research: Emergence of Y‐Series Electron Acceptors and Their Perspectives,” ACS Energy Letters 5 (2020): 1554–1567.

[23] Y. Zou , H. Chen , X. Bi , et al., “Peripheral Halogenation Engineering Controls Molecular Stacking to Enable Highly Efficient Organic Solar Cells,” Energy & Environmental Science 15 (2022): 3519–3533.

[24] J. Ge , L. Xie , R. Peng , and Z. Ge , “Organic Photovoltaics Utilizing Small‐Molecule Donors and Y‐Series Nonfullerene Acceptors,” Advanced Materials 35 (2023): 2206566. 10.1002/adma.202206566 36482012

[25] Y. Ding , W. A. Memon , S. Xiong , et al., “Molecular Design of Dimeric Acceptor Enables Binary Organic Solar Cells With 19.78% Efficiency and Enhanced Stability,” Advanced Materials 37 (2025): 2501671. 10.1002/adma.202501671 40025944

[26] K. Liu , Y. Jiang , F. Liu , et al., “The Critical Isomerization Effect of Core Bromination on Nonfullerene Acceptors in Achieving High‐Performance Organic Solar Cells With Low Energy Loss,” Advanced Materials 37 (2025): 2413376. 10.1002/adma.202413376 39740183

[27] L. Chen , W. Liang , A. Sergeev , et al., “Benzannulation of Furan: A Strategy for Stable and High‐Performance Furan‐Containing Giant Electron Acceptor with Efficiency Exceeding 20%,” Energy & Environmental Science 18 (2025): 6608–6617.

[28] Y. Geng , T. Li , Z. Zhang , and Y. Lin , “To Maximize Luminescence for an Efficient Organic Solar Cell †,” Chinese Journal of Chemistry 42 (2024): 3157–3168.

[29] H. Xia , C. You , J. Fu , et al., “Unveiling Energy Loss Mechanisms to Empower Ternary Organic Solar Cells With Over 20% Efficiency: A Systematic Oligomeric Approach,” Advanced Materials 37 (2025): 2501428. 40574419 10.1002/adma.202501428 PMC12447044

[30] J. Benduhn , K. Tvingstedt , F. Piersimoni , et al., “Intrinsic Non‐Radiative Voltage Losses in Fullerene‐Based Organic Solar Cells,” Nature Energy 2 (2017): 17053.

[31] D. Qian , Z. Zheng , H. Yao , et al., “Design Rules for Minimizing Voltage Losses in High‐Efficiency Organic Solar Cells,” Nature Materials 17 (2018): 703–709. 30013057 10.1038/s41563-018-0128-z

[32] S. Ullbrich , J. Benduhn , X. Jia , et al., “Emissive and Charge‐Generating Donor–Acceptor Interfaces for Organic Optoelectronics with Low Voltage Losses,” Nature Materials 18 (2019): 459–464. 30936478 10.1038/s41563-019-0324-5

[33] Q. Liu and K. Vandewal , “Understanding and Suppressing Non‐Radiative Recombination Losses in Non‐Fullerene Organic Solar Cells,” Advanced Materials 35 (2023): 2302452. 10.1002/adma.202302452 37201949

[34] H. Xia , Y. Zhang , W. Deng , et al., “Novel Oligomer Enables Green Solvent Processed 17.5% Ternary Organic Solar Cells: Synergistic Energy Loss Reduction and Morphology Fine‐Tuning,” Advanced Materials 34 (2022): 2107659. 10.1002/adma.202107659 34997631

[35] H. Yu , Y. Wang , C. H. Kwok , et al., “A Polymer Acceptor with Double‐Decker Configuration Enhances Molecular Packing for High‐Performance All‐Polymer Solar Cells,” Joule 8 (2024): 2304–2324.

[36] Z. Chen , H. Yao , J. Wang , et al., “Restrained Energetic Disorder for High‐Efficiency Organic Solar Cells via a Solid Additive,” Energy & Environmental Science 16 (2023): 2637–2645.

[37] Y. Wang , J. Yu , R. Zhang , et al., “Origins of the Open‐Circuit Voltage in Ternary Organic Solar Cells and Design Rules for Minimized Voltage Losses,” Nature Energy 8 (2023): 978–988.

[38] Y. Chen , X. Duan , J. Zhang , et al., “Reducing Energy Loss by Developing Luminescent Triphenylamine Functionalized Electron Acceptor for High Performance Organic Solar Cells,” Energy & Environmental Science 18 (2025): 6214–6223.

[39] J. Mei , N. L. Leung , R. T. Kwok , J. W. Lam , and B. Z. Tang , “Aggregation‐Induced Emission: Together We Shine, United We Soar!,” Chemical Reviews 115 (2015): 11718–11940. 26492387 10.1021/acs.chemrev.5b00263

[40] X. Feng , X. Wang , C. Redshaw , and B. Z. Tang , “Aggregation Behaviour of Pyrene‐Based Luminescent Materials, From Molecular Design and Optical Properties to Application,” Chemical Society Reviews 52 (2023): 6715–6753. 37694728 10.1039/d3cs00251a

[41] A. H. Fallahpour , S. Kienitz , and P. Lugli , “Origin of Dark Current and Detailed Description of Organic Photodiode Operation Under Different Illumination Intensities,” IEEE Transactions on Electron Devices 64 (2017): 2649–2654.

[42] Y. Zhang , Y. Yu , X. Liu , et al., “An n‐Type All‐Fused‐Ring Molecule With Photoresponse to 1000 nm for Highly Sensitive Near‐Infrared Photodetector,” Advanced Materials 35 (2023): 2211714. 10.1002/adma.202211714 36842062

[43] O. J. Sandberg , C. Kaiser , S. Zeiske , et al., “Mid‐Gap Trap State‐Mediated Dark Current in Organic Photodiodes,” Nature Photonics 17 (2023): 368–374.

[44] Y. Zhang , N. Wang , Y. Wang , J. Zhang , J. Liu , and L. Wang , “All‐Polymer Indoor Photovoltaic Modules,” iScience 24 (2021): 103104. 34611609 10.1016/j.isci.2021.103104 PMC8476653

[45] Y. Yu , Y. Zhang , J. Miao , J. Liu , and L. Wang , “An n ‐Type All‐Fused‐Ring Molecule With Narrow Bandgap,” CCS Chemistry 5 (2023): 486–496.

[46] B. Wu , Y. Li , K. Liu , et al., “An Asymmetric Polymerized Small Molecular Acceptor with Temperature‐Dependent Aggregation and Superior Batch‐To‐Batch Reproducibility for Efficient All‐Polymer Solar Cells,” Nano Energy 128 (2024): 109874.

[47] J. Wang , C. Sun , Y. Li , et al., “Polymer‐Like Tetramer Acceptor Enables Stable and 19.75% Efficiency Binary Organic Solar Cells,” Nature Communications 16 (2025): 1784. 10.1038/s41467-025-57118-9 PMC11840019 39971926

[48] Y. Jiang , K. Liu , F. Liu , et al., “20.6% Efficiency Organic Solar Cells Enabled by Incorporating a Lower Bandgap Guest Nonfullerene Acceptor Without Open‐Circuit Voltage Loss,” Advanced Materials 37 (2025): 2500282. 10.1002/adma.202500282 40018842

[49] Y. Ge , Y. Wu , Y. Hai , et al., “Aggregation Engineering of Toluene‐Processed Acceptor Layer Enables Over 19% Efficiency of Air‐Blade‐Coated Organic Solar Cells,” Advanced Materials 37 (2025): 2502579. 10.1002/adma.202502579 40297926

[50] Y. Shi , Y. Chang , K. Lu , et al., “Small Reorganization Energy Acceptors Enable Low Energy Losses in Non‐Fullerene Organic Solar Cells,” Nature Communications 13 (2022): 3256. 10.1038/s41467-022-30927-y PMC9174259 35672325

[51] Z. Li , H. Yao , Z. Chen , et al., “A New Nonfullerene Acceptor with Suppressed Energy Disorder for High‐Efficiency Organic Solar Cells,” CCS Chemistry 6 (2024): 2749–2757.

[52] M. Yang , B. Yin , G. Hu , et al., “Sensitive Short‐Wavelength Infrared Photodetection with a Quinoidal Ultralow Band‐Gap n‐Type Organic Semiconductor,” Chem 10 (2024): 1425–1444.

[53] L. Shao , J. Yang , Y. Huang , et al., “Molecular Order Control of Nonfullerene Acceptors Enables Ultralow Dark Current and High Responsivity in Short‐Wavelength Infrared Organic Photodetectors,” Chemistry of Materials 36 (2024): 5775–5787.

[54] M. Azzouzi , T. Kirchartz , and J. Nelson , “Factors Controlling Open‐Circuit Voltage Losses in Organic Solar Cells,” Trends in Chemistry 1 (2019): 49–62.

[55] D. He , F. Zhao , C. Wang , and Y. Lin , “Non‐Radiative Recombination Energy Losses in Non‐Fullerene Organic Solar Cells,” Advanced Functional Materials 32 (2022): 2111855.

[56] N. Wei , Y. Guo , H. Song , Y. Liu , H. Lu , and Z. Bo , “Reducing Non‐Radiative Energy Losses in Non‐Fullerene Organic Solar Cells,” Chemsuschem 18 (2025): 202402169. 10.1002/cssc.202402169 39483107

[57] L. Xue , Q. Xie , W. Xie , et al., “Precise Side‐Chain Engineering Optimizes Polymer Pre‐Aggregation and Crystallinity for Efficient Organic Solar Cells With Minimized Non‐Radiative Energy Loss,” Aggregate 6 (2025): 70103.

