# Supplementary material for: Aggregation‐Induced Emission Molecular Design for Mitigating Non‐Radiative Energy Loss in Organic Solar Cells
Source: Adv Mater. 2026 Jan 20;38(12):e19588. doi: 10.1002/adma.202519588 (PMC12933007; doi:10.1002/adma.202519588)
Supplement: Supplementary file 2 — Supporting File 2: adma72166‐sup‐0002‐SuppMat.docx. [file ADMA-38-e19588-s001.docx]

Supporting Information

Aggregation-Induced Emission Molecular Design for Mitigating Non-Radiative Energy Loss in Organic Solar Cells

Yingze Zhang, Rongkun Zhou, Mingjie Rong, Xianghao Zeng, Ho Ming Ng, Ruijie Ma, Joshua Yuk Lin Lai, Chao Li, Shuwei Qiu, Heng Liu, Hao Xia, Lei Zhu, Guangye Zhang, Xinhui Lu, Zilong Zheng, Jun Liu, He Yan*, Sai Ho Pun*, Gang Li*

1. **Experimental Section**

*Materials and general characterization:* Polymer donors D18 were purchased from Solar Materials Inc. L8BO-C4 was synthesized by Dr. C. Li. L8BO-C4 was selected as the host acceptor because it represents the state-of-the-art, high-performance electron acceptor, which can achieve higher PCE in devices than L8BO.[^1^](#_ENREF_1) Unless noted otherwise, all the reagents and chemicals were purchased from commercial sources and used as received. Details of the synthetic procedures for dTPE and dSpiro and the corresponding structural characterization are presented in Supporting information. Nuclear magnetic resonance spectra were recorded using a Bruker AV 400 MHz NMR spectrometer using deuterated chloroform as the solvent and trimethylsilane as the internal reference at room temperature. Mass spectra were recorded using a MALDI Micro MX mass spectrometer. Uv-vis absorption spectra were measured by using a Shimadzu (model UV-3700) UV-Vis-NIR spectrophotometer.

*Cyclic voltammetry measurements:* were performed under a nitrogen atmosphere at a scan rate of 100 mV s^−1^ using a Zahner IM6e electrochemical workstation. A platinum plate coated with sample film was used as the working electrode, a platinum wire was used as the counter electrode and a saturated Ag/Ag^+^ electrode was used as the reference electrode. Tetra-n-butylammonium hexafluorophosphate (0.1 M) in anhydrous acetonitrile solution was used as the supporting electrolyte, and ferrocene/ferrocenium was used as the internal standard. Therefore, energy levels for the HOMO and the LUMO could be obtained from equations *E*_HOMO_= (*E*_ox_+ 4.33) eV and *E*_LUMO_ = −(*E*_red_ + 4.33) eV, respectively, where *E*_ox_ is the onset oxidation/reduction potential relative to Ag/AgCl.

*Device fabrication and characterization:* Organic solar cells (OSCs) were fabricated with a structure of ITO/2PACz/active layer/PDINN/Ag. Herein, 2PACz serves as the hole transport material and PDINN as the electron transport material. The ITO-coated substrates were ultrasonically cleaned sequentially with deionized water, acetone, and isopropyl alcohol, each for 15 minutes. The cleaned substrates were stored in isopropanol until use. Prior to film deposition, the substrates were dried under N₂ flow and treated with plasma cleaning for 3 minutes under a vacuum below 10 Pa. Subsequently, the substrates were transferred into an N₂ glovebox. A 2PACz monolayer (0.35 mg mL⁻¹ in ethanol) was spin-coated onto the ITO substrates at 3000 rpm for 30 s, followed by thermal annealing at 100 °C for 10 min. The active layers for the D18:L8BO-C4, D18:dTPE, D18:dSpiro, and D18:L8BO-C4:dTPE systems were prepared by spin-coating a blend of D18 and the respective acceptor(s) from chloroform solution (containing 6 mg mL⁻¹ trichlorobenzene as a solvent additive) at a total concentration of 11 mg mL⁻¹ onto the 2PACz layer. The optimal thickness of the active layer was approximately 110 nm. The weight ratios of D18 to acceptor were 1:1.2 for the binary systems, and 1:1:0.2 for the ternary D18:L8BO-C4:dTPE system. The active layers were then thermally annealed at 90 °C for 8 min. Next, a PDINN layer (1.1 mg mL⁻¹ in methanol) was spin-coated at 3000 rpm for 30 s. Finally, a 100-nm-thick Ag electrode was thermally evaporated under a vacuum of 2×10⁻⁴ Pa. The devices, with an active area of 4.1 mm², were tested through a mask defining an aperture of 2.25 mm². Current density–voltage (*J*–*V*) characteristics were measured under AM 1.5G illumination (100 mW cm⁻²) using a solar simulator (Enlitech SS-F5-3A), with the light intensity calibrated using a certified silicon reference cell (Enlitech SRC2020). The *J*–*V* curves were recorded with a Keithley 2400 Source Measure Unit. External quantum efficiency (EQE) spectra were acquired using an Enlitech QE-R3011 measurement system. The impedance tests were conducted using PARSTAT 3000A potentiostat at a frequency range from 10^5^ Hz to 1 Hz, with an applied voltage of 0 V and AC signal of 0.1.

*Carrier mobility tests:* SCLC method was used to study the carrier mobility of the neat and blend films. Hole-only devices were fabricated with a structure of is ITO/PEDOT:PSS (30 nm)/active layer/MoO_3_ (10 nm)/Ag (100 nm), and electron-only devices were fabricated with a structure of ITO/ZnO (15 nm)/active layer/PDINN (10 nm) /Ag (100 nm). *J*–*V* plots in the range of 0–8 V of the devices were measured using a Keithley 2400 source meter. The hole and electron mobilities were estimated using the SCLC method by fitting the *J*–*V* plots near the quadratic region according to the modified Mott-Gurney equation:

$$J=\frac{9}{8}\varepsilon_{r}\varepsilon_{0}\mu\frac{V^{2}}{L^{3}}exp\left[ 0.89\beta\frac{\sqrt{V}}{\sqrt{L}} \right]$$

where *J* is the current density, *ε*_0_ is the permittivity of free space, *ε*_r_ is the relative permittivity (assumed to be 3), *µ* is the zero-field mobility, *V* is the potential across the device (*V* = *V*_applied_ – *V*_bi_ – *V*_series_), *L* is the thickness of the active layer, and *β* is the field-activation factor.

Energy loss analysis: Fourier-transform photocurrent spectroscopy EQE (FTPS-EQE) spectra were measured by using a Vertex 70 from Bruker optics and a Quartz Tungsten-Halogen (QTH) lamp. The electroluminescence signal was collected with a monochromator and detected with a Si-CCD detector. Electroluminescence quantum efficiency (*EQE*_EL_) values were obtained from an in-house-built system, including a Hamamatsu silicon photodiode 1010B, a Keithley 2400 SourceMeter to provide voltage and record injected current, and a Keithley 485 Picoammeter to measure the emitted light intensity.

*Morphology characterization:* The AFM images were recorded using a SPA300HV (Seiko Instruments, Inc., Japan) in tapping mode. 2D-GIWAXS measurements were carried out with a Xeuss 2.0 SAXS/WAXS laboratory beamline using a Cu X-ray source (8.05 keV, 1.54 Å) and a Pilatus3R 300K detector. The incidence angle is 0.2^o^. All measurements were conducted under a vacuum environment to reduce air scattering

*Device stability characterizations:* The device encapsulation process is shown as follow: Firstly, a layer of UV-curable adhesive was evenly smeared on the electrode surface of device in glove box with N_2_ atmosphere. Subsequently, a piece of glass was put on the top of active area. Finally, the device was illuminated under UV light source (365 nm) for 10 minutes. The photostability data of the devices was measured by using Keithley 2400 source meter. The devices were stored under continuous 100 mW cm^-2^ illumination provided by LED-solar simulators in glove box (the average tested temperature was 25 ^o^C). For the thermal stability of OSCs, the devices were placed on a hot plate with a constant temperature of 85 ^o^C annealing in glove box. The devices were also measured by using the source meter.

*Calculation methods.* The MD simulations were performed via GROMACS (version 2018.4) software package.[^2^](#_ENREF_2)^,^ [^3^](#_ENREF_3) Velocity-rescale thermostat was applied for the temperature, and Berendsen thermostat was used to maintain a pressure of 1 atm. Two-dimensional periodic boundary conditions and the general amber force field (GAFF) were employed.^[4](#_ENREF_4)^ PME (Particle-Mesh Ewald) electrostatics were applied to calculate the long-range electrostatic interactions. The short-range electrostatic and van der Waals cutoff distance were both set to 1.2 nm. The equilibrium bond lengths and angles for the optimized geometries of D18, L8-BO-C4, dTPE, chloroform (CF), and Trichlorobenzene (TCB) molecules were calculated at the B3LYP/6-311G(d,p) level of DFT. The parameter refinement was achieved through frequency analysis using the Sobtop software. The atomic partial charges were fitted using the RESP (restrained electrostatic potential) method^[5](#_ENREF_5)^ via Multiwfn (version 3.6) software package.^[6](#_ENREF_6)^ The simulation boxes contained 75 D18 pentamer, 390 L8-BO-C4 for D18:L8-BO-C4 blend and contained 75 D18 pentamer, 340 L8-BO-C4, 50 dTPE for D18:L8-BO-C4:dTPE blend. Equilibration simulations of the active material solutions were conducted for 300 ns to establish self-assembly configurations. The number of CF and TCB were set to 25000 and 3750, respectively. The solvent evaporation dynamics were governed by distinct evaporation rates. In each 0.5 ns interval, we randomly removed 100 CF molecules from the box. In contrast, TCB was removed at a much slower rate of 10 molecules per interval. These rates were validated to ensure system re-equilibration between solvent extraction steps. A two-step evaporation process was simulated. CF was completely removed, followed by gradual TCB evaporation.

*Estimation of glass-transition temperature.* UV-vis spectroscopy was used to determine the glass transition temperature (*T*_g_). The absorption spectra of star-shaped acceptor films were measured with increasing temperatures from 20 to 200 °C. For the preparation of films, we used the same processing conditions as those for the OSC fabrication, to precisely correlate the estimated *T*_g_ with those in the OSC device. Then, the deviation metric (DMT) of each absorption spectra was calculated, following the method reported by Harald Ade.[^7^](#_ENREF_7)^,^ [^8^](#_ENREF_8)

$$DMT=\sum_{\lambda_{min}}^{\lambda_{max}} \left[ IRT\left( \lambda\right)-IT\left( \lambda\right) \right]$$

where *λ* is the absorption wavelength, *λ*_max_ and *λ*_min_ are the upper and lower bounds of the optical sweep, respectively, IRT(*λ*) and IT(*λ*) are the normalized absorption intensities of the as-cast and annealed films, respectively. Then, the *T*_g_ is determined to be the point where the two interpolated lines in low- and high-temperature regions intersect.

1. **Syntheses and characterizations**

**Reagents:** Tetrahydrofuran (THF), toluene, and o-dichlorobenzene (o-DCB) were dried using sodium or calcium hydroxide before use.


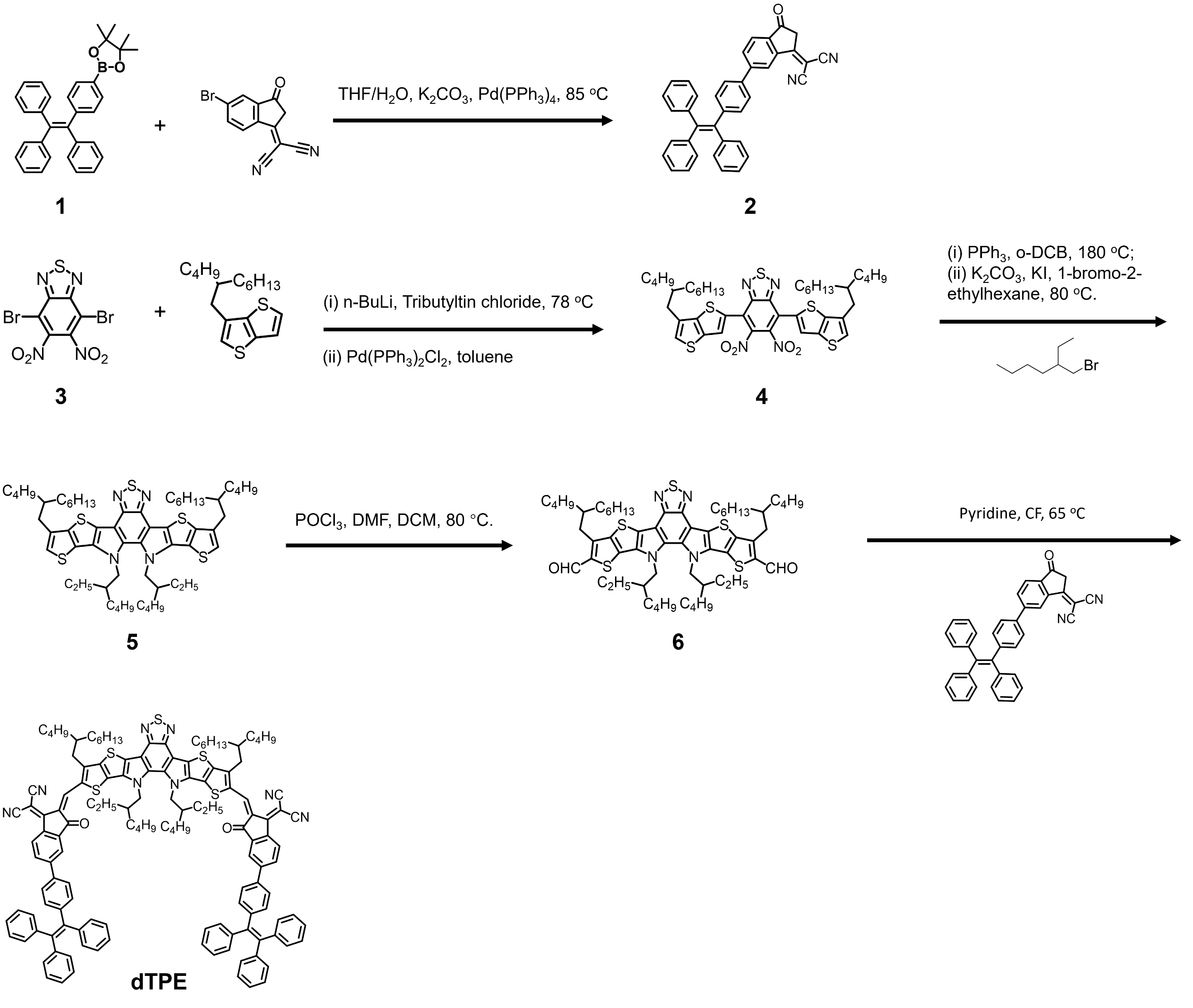


**Scheme S1.** Detailed synthesis routes of dTPE

**Compound 2:** Starting materials of compound **1** (1000 mg, 2.18 mmol) and 2-(5-bromo-3-oxo-2,3-dihydro-1H-inden-1-ylidene)malononitrile (200 mg, 0.73 mmol) were dissolved into tetrahydrofuran (25 mL) in a two-necked flask under argon. K_2_CO_3_ (553 mg, 4 mmol) dissolved in water (5 mL) was added to the two-necked flask. The solution was flushed with nitrogen for 10 min. Then, add Pd (PPh_3_)_4_ (70 mg) to the solution, then let the solution temperature to 85°C for 6 h. Washed with saturated salt water and ethyl acetate. The solvent was removed under reduced pressure. Finally, the residue was purified by silica gel chromatography (n-hexane/dichloromethane = 1:4, v/v) to afford compound **2** (360 mg, yield: 94.1%) as an orange solid. ^1^H NMR (400 MHz, CDCl_3_) δ (ppm) 8.65 (d, J = 8.4 Hz, 1H), 8.13 (d, J = 1.7 Hz, 1H), 8.07 (dd, J = 8.5, 1.9 Hz, 1H), 7.47-7.43 (m, 2H), 7.21-7.00 (m, 17H), 3.76 (s, 2H).

**Compound 4:** Under nitrogen protection, n-BuLi (2.8 mL, 7 mmol, 2.5 M in hexane) was added dropwise to the stirring solution of 3-(2-butyloctyl)thieno[3,2-b]thiophene (2.16 g, 7 mmol) in THF (64 mL) at -78 °C. After stirring at -78 °C for 1.5 hours, Tri-n-butyltin chloride (3.52 g, 10.8 mmol) was added. The solution was then allowed to warm to room temperature and stirred overnight. The mixture was concentrated under reduced pressure to get crude tributylstannane-substituted derivative, which was used directly to the next reaction. To solution of compound **3** (1.03 g, 2.68 mmol) and Pd(PPh_3_)_2_Cl_2_ (97 mg, 0.14 mmol) in toluene (48 mL) was added tributylstannane-substituted derivative (7 mmol) under nitrogen protection. The reaction mixture was heated to reflux and stirred overnight. After cooling to room temperature, the mixture was added water and extracted with dichloromethane. The organic phase was concentrated under reduced pressure to provide crude product, which was further purified with silica gel chromatography (n-hexane/dichloromethane = 4:1, v/v) to afford compound **4** (1.90 g, yield: 84.9%) as a red solid. ^1^H NMR (400 MHz, CDCl_3_): δ 7.71 (s, 2H), 7.15 (s, 2H), 2.72-2.70 (d, 4H), 1.87 (m, 2H), 1.32-1.27 (m, 32H), 0.89-0.85 (t, 12H).

**Compound 5:** Under nitrogen atmosphere, P(OEt)_3_ (10 mL) was added to the solution of compound **4** (1.68g, 2 mmol) in o-DCB (30 mL). The reaction mixture was heated to reflux at 180 ^o^C and stirred overnight. After cooling to room temperature, the solvent was removed under reduced pressure and the residue was added to 100 mL two-necked round bottom flask which containing 1-bromo-2-ethylhexane (6.19 g, 32 mmol), K_2_CO_3_ (4.42 g, 32 mmol), KI (5.32 g, 32 mmol) and DMF 60 mL). The reaction mixture was heated at 80 °C and stirred overnight. After cooling to room temperature, the mixture was added water and extracted with dichloromethane for three times. The combined organic phase was further washed with water, dried over MgSO_4_, filtrated and concentrated under reduced pressure. The organic phase was concentrated under reduced pressure to provide crude product, which was further purified with silica gel chromatography (n-hexane/dichloromethane = 8:1, v/v) to afford compound **5** (1.46 g, yield: 73%) as a yellow solid. ^1^H NMR (400 MHz, CDCl_3_): δ 6.97 (s, 2H), 4.59 (dd, J = 7.7, 2.5 Hz, 4H), 2.74 (d, J = 7.2 Hz, 4H), 2.06-2.00 (m, 4H), 1.39-1.22 (m, 36H), 0.92-0.83 (m, 24H), 0.66-0.58 (m, 12H).

**Compound 6:** Under nitrogen protection, POCl_3_ (4 mL) was added dropwise to a solution of anhydrous N,N-Dimethylformamide (DMF) (40 mL) at 0 °C and stirred at room temperature for another 30 min. Then, compound **5** (0.80 g, 0.8 mmol) dissolved in 1,2-dichloroethane (40 mL) was added to the mixture and stirred at 80 ºC overnight. The mixture was cooled to room temperature and extracted with dichloromethane. The combined organic extracts were washed with water for three times, dried with anhydrous MgSO_4_, filtered, and the solvent was removed under reduced pressure. The obtained crude product was further purified by column chromatography on silica gel with a mixture solvent of n-hexane and dichloromethane (2:3, v:v) as an eluent to afford compound **6** (0.74 g, yield: 87.9%) as a yellow solid. ^1^H NMR (400 MHz, CDCl_3_): δ 10.13 (s, 2H), 4.73-4.57 (d, 4H), 3.12 (d, J = 7.5 Hz, 4H), 2.11-1.98 (m, 4H), 1.50-1.21 (m, 36H), 0.98-0.82 (m, 24H), 0.71-0.66 (m, 6H), 0.62-0.57 (m, 6H).

**dTPE:** Compound **6** (63 mg, 0.06 mmol) and compound **2** (157 mg, 0.3 mmol) were dissolved into dry chloroform (20 mL) in a three-neck flask. The solution was flushed with nitrogen for 30 min. After 0.3 mL pyridine was added, the mixture was stirred at 65°C for 4 h. After cooling to room temperature, the reaction mixture was poured into water and extracted several times with chloroform. Then the solvent was removed under reduced pressure, and the crude product was purified by column chromatography on silica gel to yield dTPE as a black solid (113 mg, 91.1% yield) ^1^H NMR (400 MHz, CDCl_3_): δ 9.14-9.07 (s, 2H), 8.68 (d, J = 8.3 Hz, 2H), 8.68 (d, *J* = 8.3 Hz, 2H), 7.86 (d, J = 8.3 Hz, 2H), 7.43 (d, J = 8.3 Hz, 4H), 7.11-7.04 (m, 34H), 4.79 (d, J = 7.5 Hz, 4H), 3.09 (d, J = 6.9 Hz, 4H), 2.16-1.99 (m, 4H), 1.32-1.13 (m, 36H), 1.05-0.72 (m, 30H), 0.66-0.63 (m, 6H). MS (MALDI-TOF) m/z: [M+H]^+^ calcd for C_136_H_130_N_8_O_2_S_5_, 2068.89, found: 2068.90. ^13^C NMR (400 MHz, CDCl_3_) δ = 187.81, 160.22, 151.92, 146.97, 146.06, 144.47, 144.40, 142.86, 142.81, 142.74, 141.47, 139.37, 137.96, 137.14, 137.05, 135.54, 134.69, 134.35, 133.46, 132.89, 132.41, 131.67, 130.76, 130.74, 130.67, 129.21, 127.27, 127.24, 127.09, 126.19, 126.10, 125.79, 125.02, 120.84, 120.48, 115.10, 114.59, 112.78, 66.99, 55.00, 39.63, 39.31, 33.99, 32.93, 32.69, 31.20, 29.02, 28.23, 26.93, 25.96, 22.61, 22.37, 22.16, 22.02, 13.45, 13.43, 13.08, 9.67.


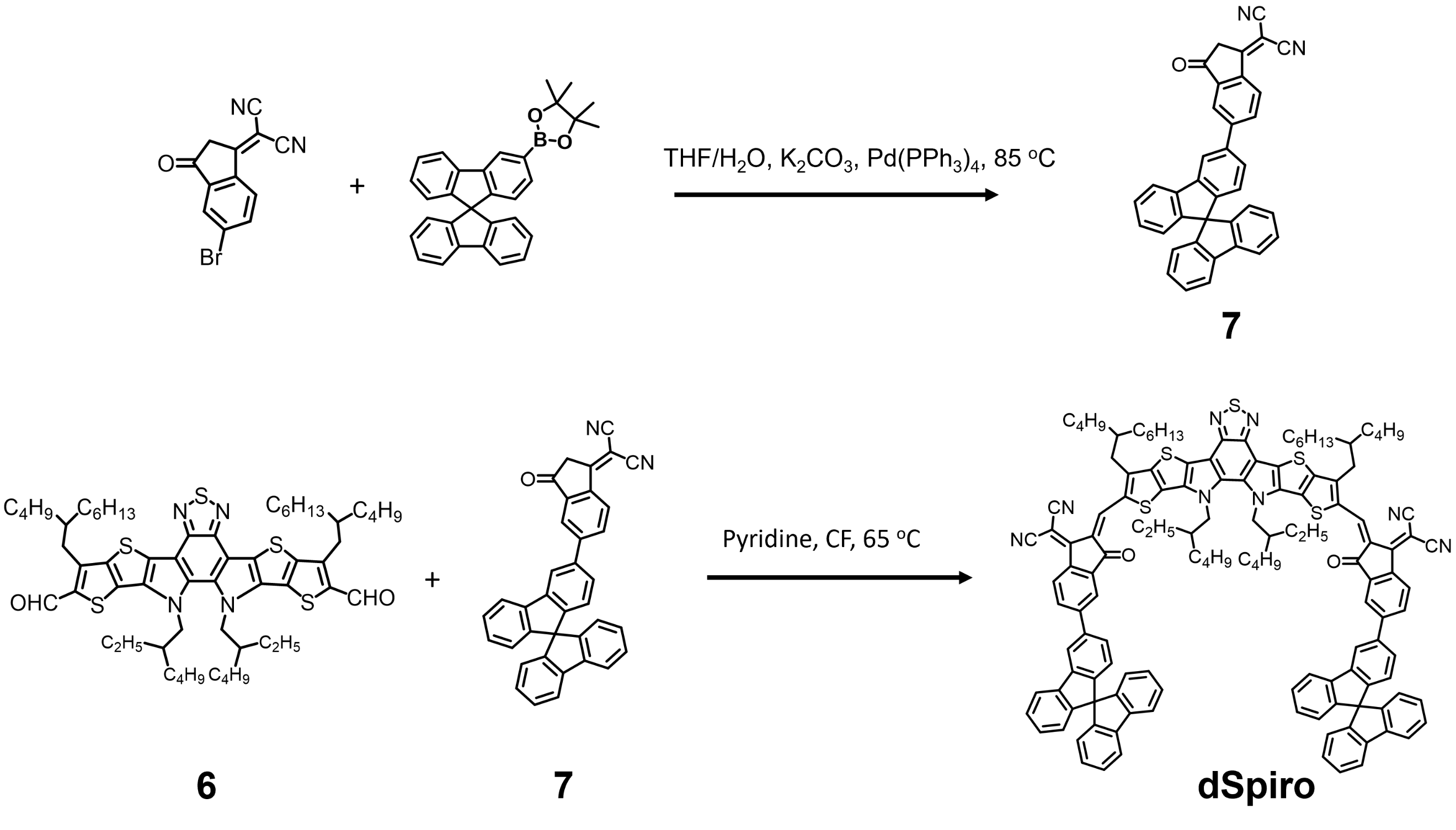


**Scheme S2.** Detailed synthesis routes of dSpiro

**Compound 7:** Starting materials of compound **1** (964 mg, 2.18 mmol) and 2-(5-bromo-3-oxo-2,3-dihydro-1H-inden-1-ylidene)malononitrile (200 mg, 0.73 mmol) were dissolved into tetrahydrofuran (25 ml) in a two-necked flask under argon. K_2_CO_3_ (553 mg, 4 mmol) dissolved in water (5 ml) was added to the two-necked flask. The solution was flushed with nitrogen for 10 min. Then, add Pd (PPh_3_)_4_ (70 mg) to the solution, then let the solution temperature to 85°C for 6 h. Washed with saturated salt water and ethyl acetate. The solvent was removed under reduced pressure. Finally, the residue was purified by silica gel chromatography (n-hexane/dichloromethane = 1:3, v/v) to afford compound **2** (338 mg, yield: 91.3%) as an orange solid. ^1^H NMR (400 MHz, CDCl_3_) δ (ppm) 8.75 (d, *J* = 8.4 Hz, 1H), 8.28 (d, *J* = 1.8 Hz, 1H), 8.20 (dd, *J* = 8.4, 1.8 Hz, 1H), 8.16 (d, *J* = 1.7 Hz, 1H), 7.95 (d, *J* = 7.6 Hz, 1H), 7.89 (d, *J* = 7.6 Hz, 2H), 7.48-7.38 (m, 4H), 7.22-7.12 (m, 3H), 6.89 (d, *J* = 7.9 Hz, 1H), 6.82-6.74 (m, 3H), 3.81 (s, 2H).

**dSpiro:** Compound **6** (63 mg, 0.06 mmol) and compound **7** (152 mg, 0.3 mmol) were dissolved into dry chloroform (20 mL) in a three-neck flask. The solution was flushed with nitrogen for 30 min. After 0.3 mL pyridine was added, the mixture was stirred at 65°C for 4 h. After cooling to room temperature, the reaction mixture was poured into water and extracted several times with chloroform. Then the solvent was removed under reduced pressure, and the crude product was purified by column chromatography on silica gel to yield dSpiro as a black solid (107 mg, 87.3% yield) ^1^H NMR (400 MHz, CDCl_3_): δ 9.16 (s, 2H), 8.78 (d, J = 8.2 Hz, 2H), 8.27 (d, J = 1.8 Hz, 2H), 8.19 (d, J = 1.7 Hz, 2H), 8.08 – 8.01 (m, 2H), 7.95 (d, J = 7.6 Hz, 2H), 7.91 (d, J = 7.7 Hz, 4H), 7.49-7.40 (m, 8H), 7.24-7.14 (m, 6H), 6.89 (d, J = 7.9 Hz, 2H), 6.82 (d, J = 7.6 Hz, 6H), 4.84 (d, J = 7.9 Hz, 4H), 3.16 (d, J = 7.2 Hz, 4H), 2.21-2.04 (m, 4H), 1.29-1.22 (m, 36H), 0.90-0.79 (m, 30H), 0.69-0.65 (m, 6H). MS (MALDI-TOF) m/z: [M+H]^+^ calcd for C_134_H_122_N_8_O_2_S_5_, 2036.80, found: 2036.75.

1. **Density functional theory calculations**


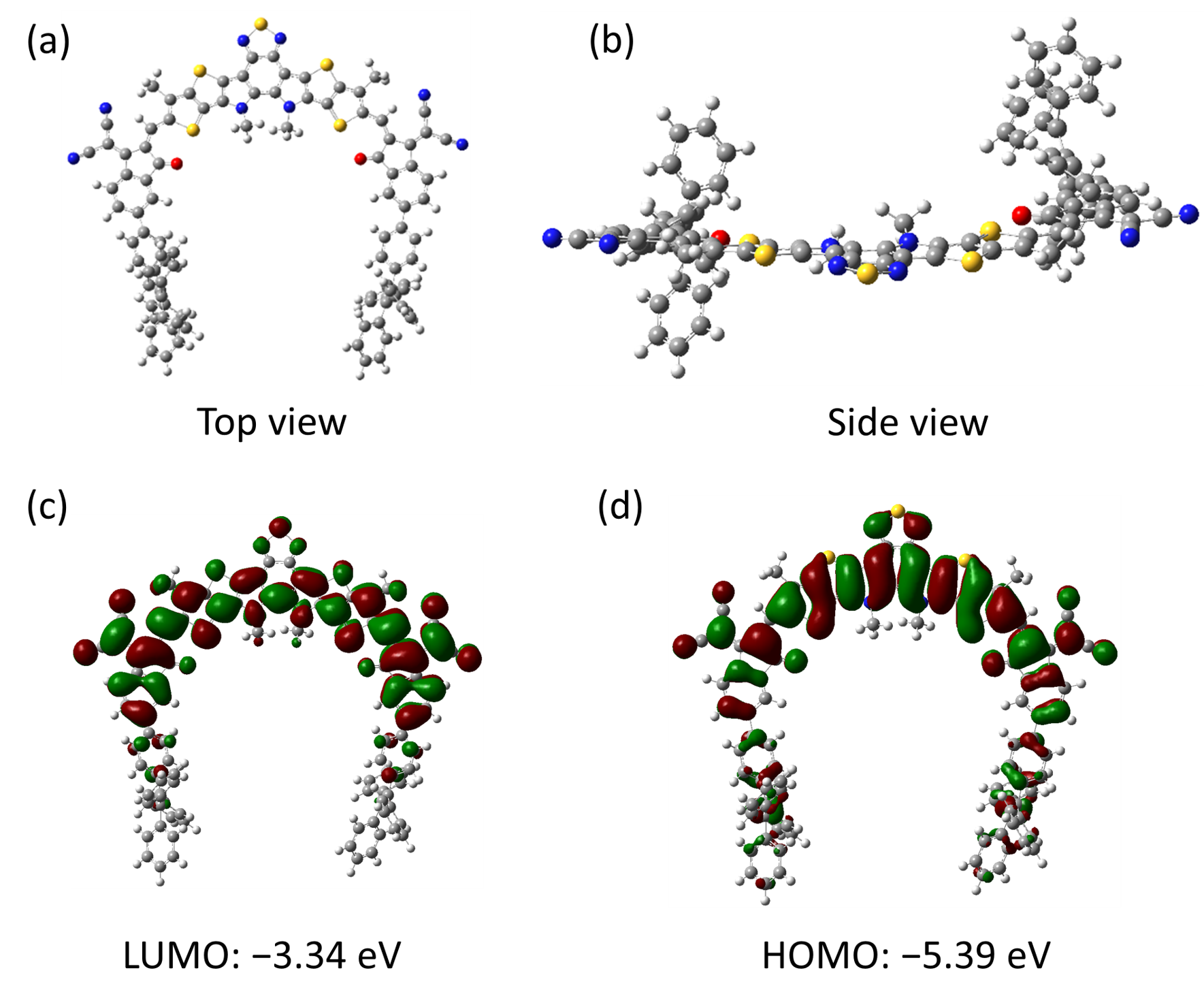


**Figure S1.** The (a) top view and (b) side view of the geometry-optimized structure for the model molecule of dTPE with all the long alkyl chains being replaced by methyl groups for simplification. The Kohn−Sham (c) LUMO and (d) HOMO patterns of the model molecule.


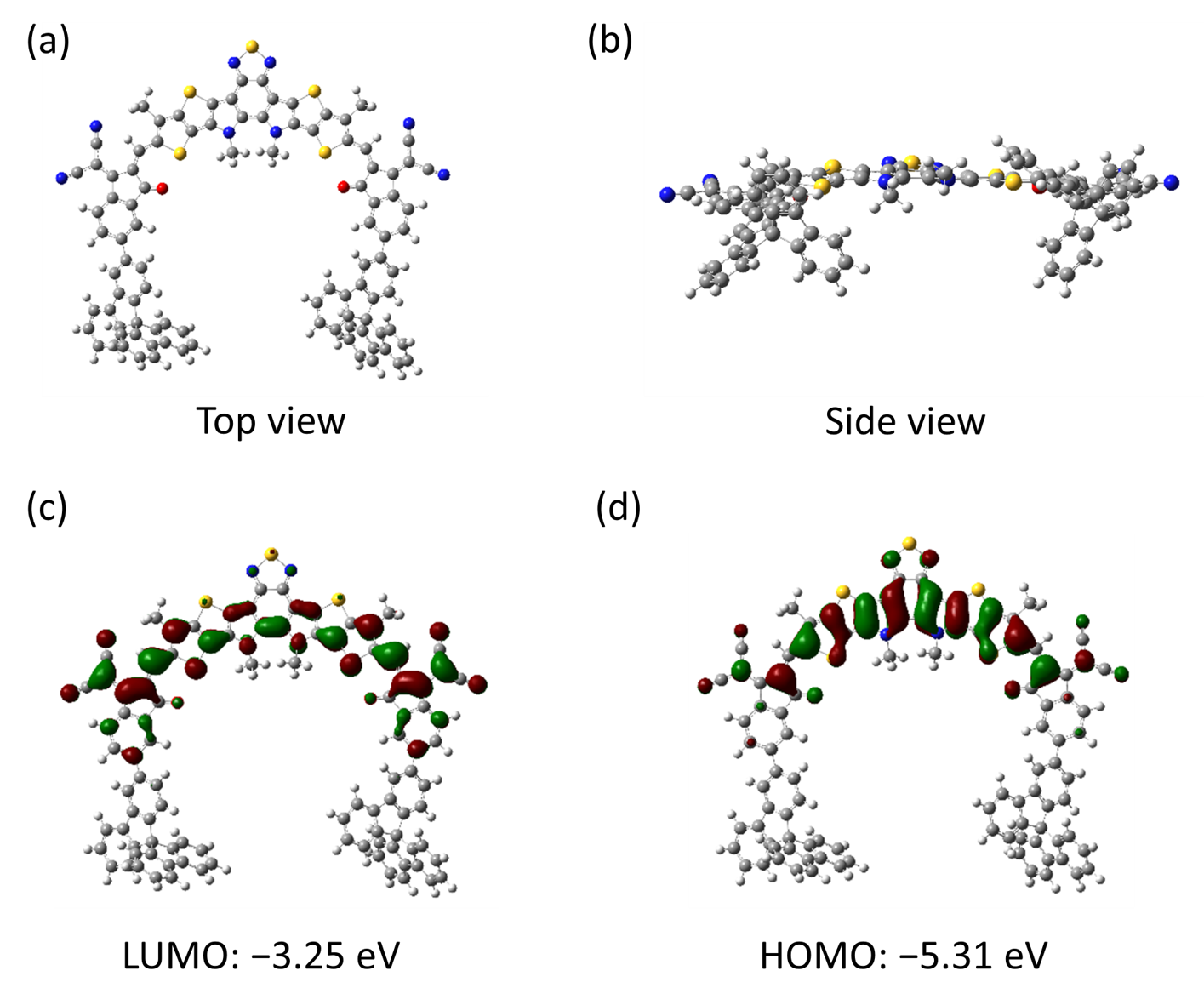


**Figure S2.** The (a) top view and (b) side view of the geometry-optimized structure for the model molecule of dSpiro with all the long alkyl chains being replaced by methyl groups for simplification. The Kohn−Sham (c) LUMO and (d) HOMO patterns of the model molecule.

1. **Basic optical and electrical measurements**


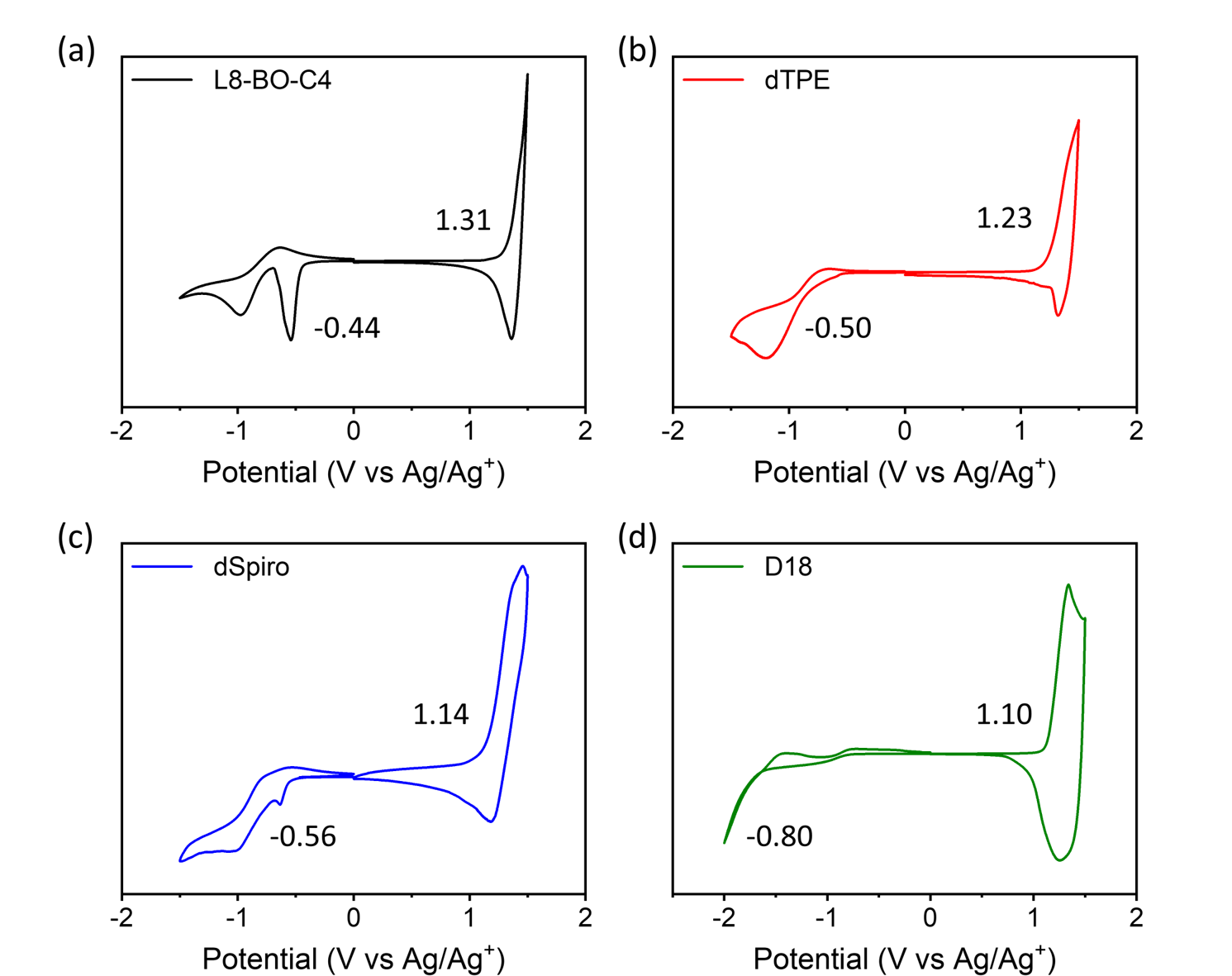


**Figure S3.** Cyclic voltammograms of (a) L8BO-C4, (b) dTPE, (c) dSpiro and (d) D18.


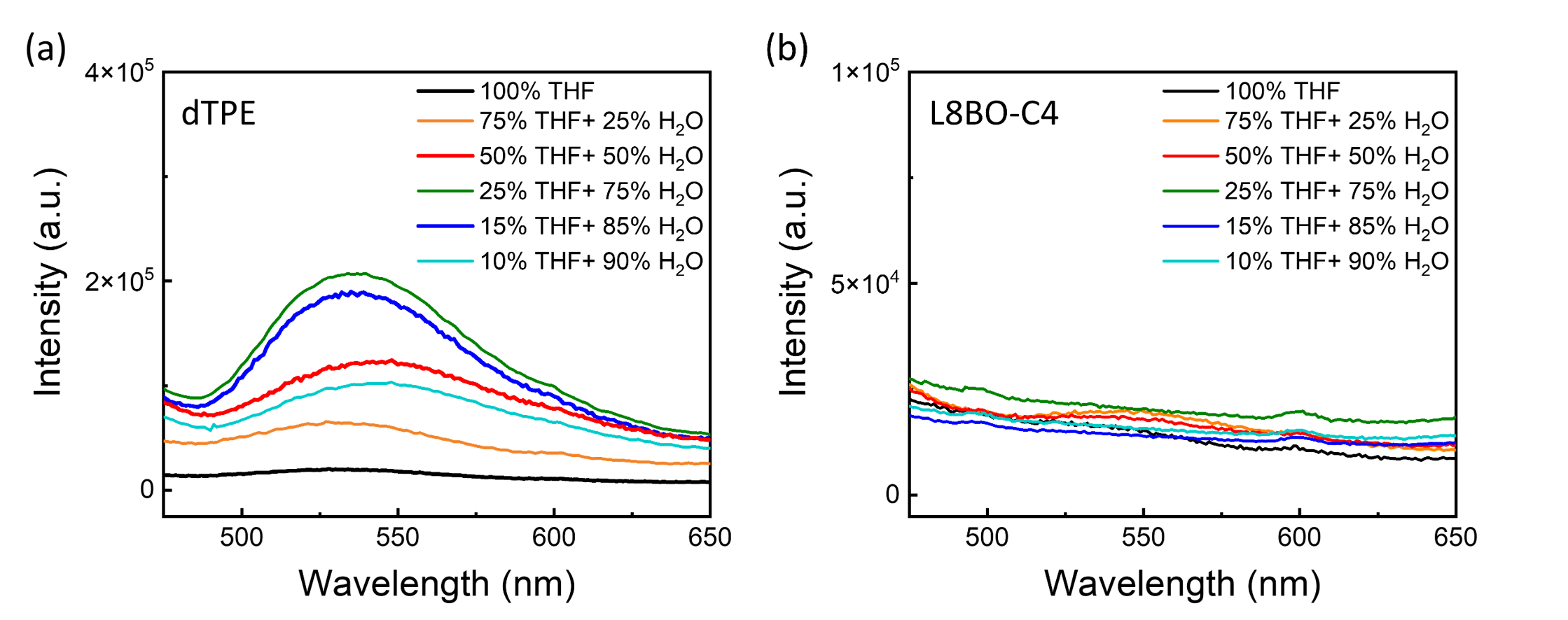


**Figure S4.** The PL spectra (excited at 365 nm, slit 5 nm) of (a) dTPE and (b) L8BO-C4 in the mixture of THF and water.


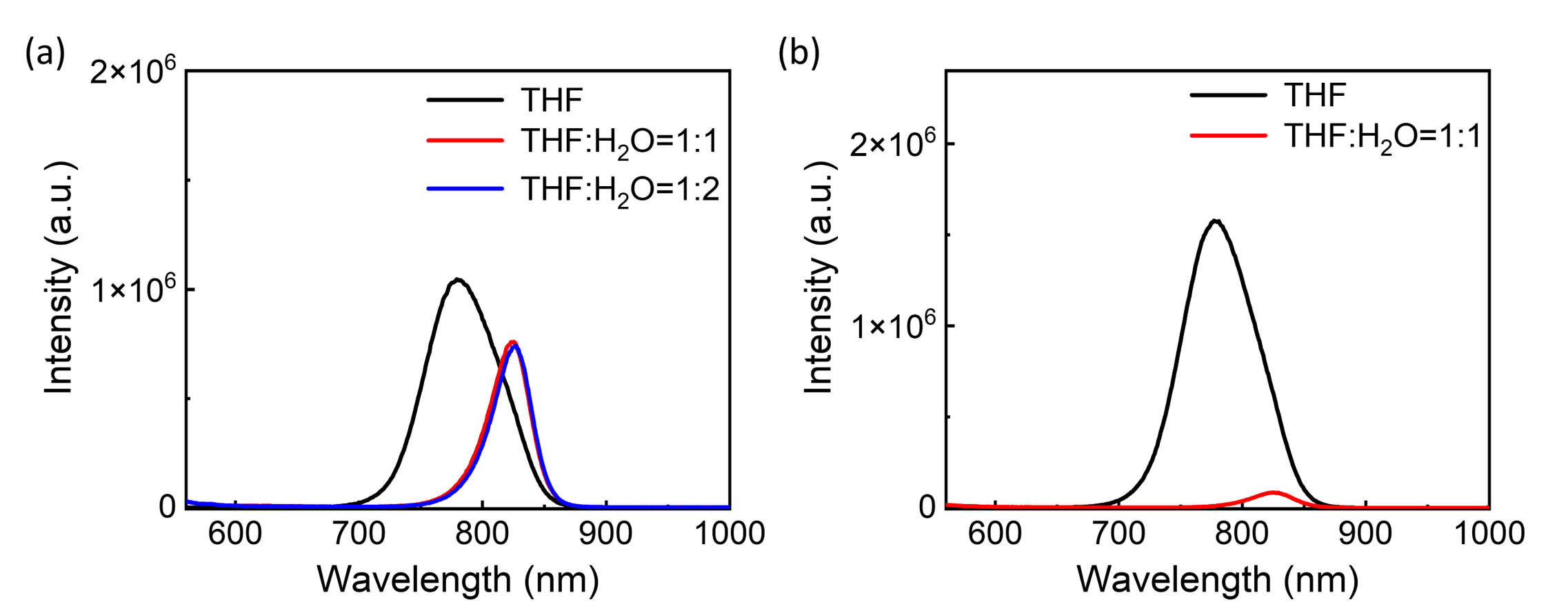


**Figure S5.** The PL spectra (excited at 530 nm, slit 5 nm) of (a) dTPE and (b) L8BO-C4 in the mixture of THF and water.


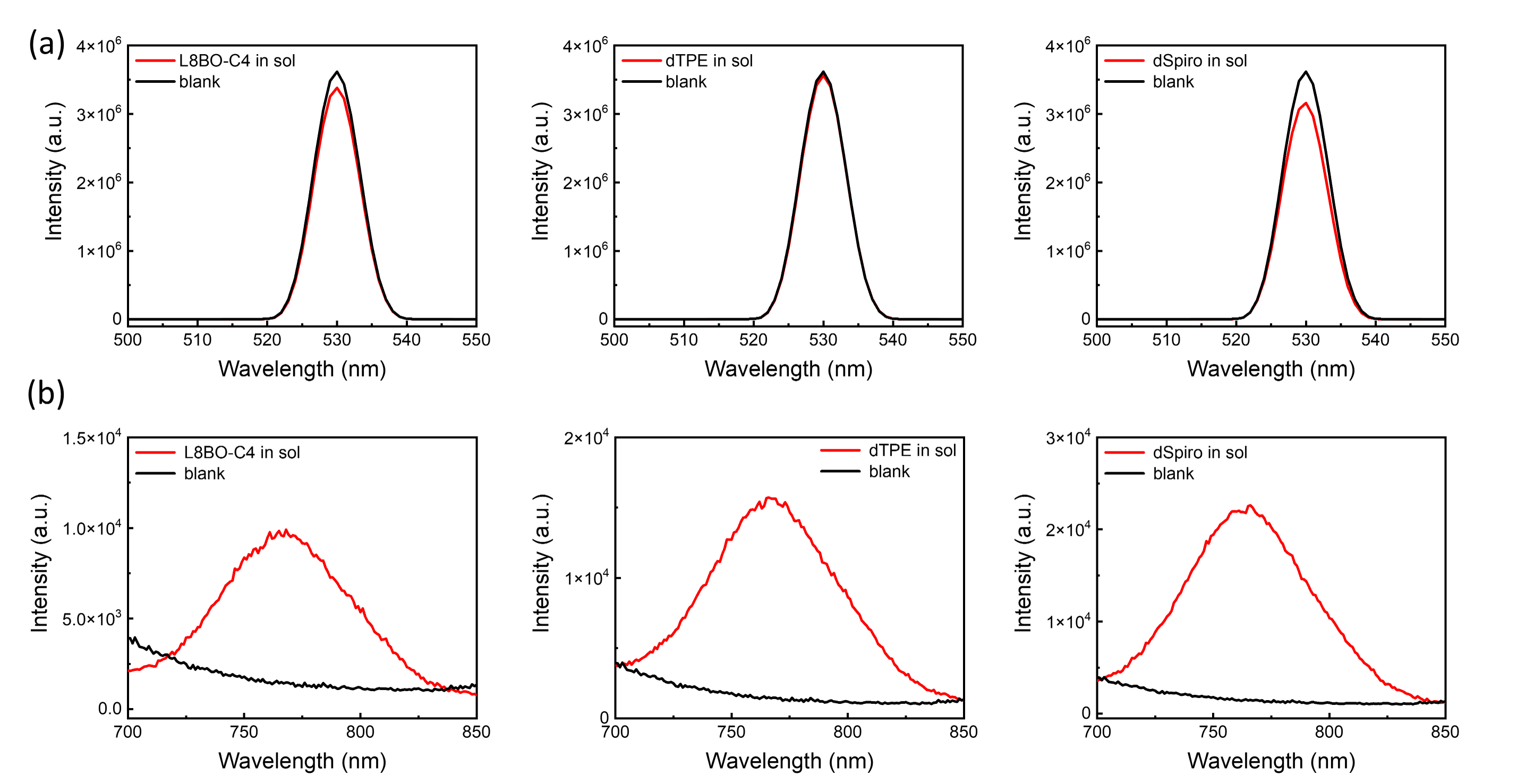


**Figure S6.** The (a) absorption and (b) emission spectra (excited at 530 nm, slit 5 nm) of L8BO-C4, dTPE and dSpiro in tetrahydrofuran solutions.


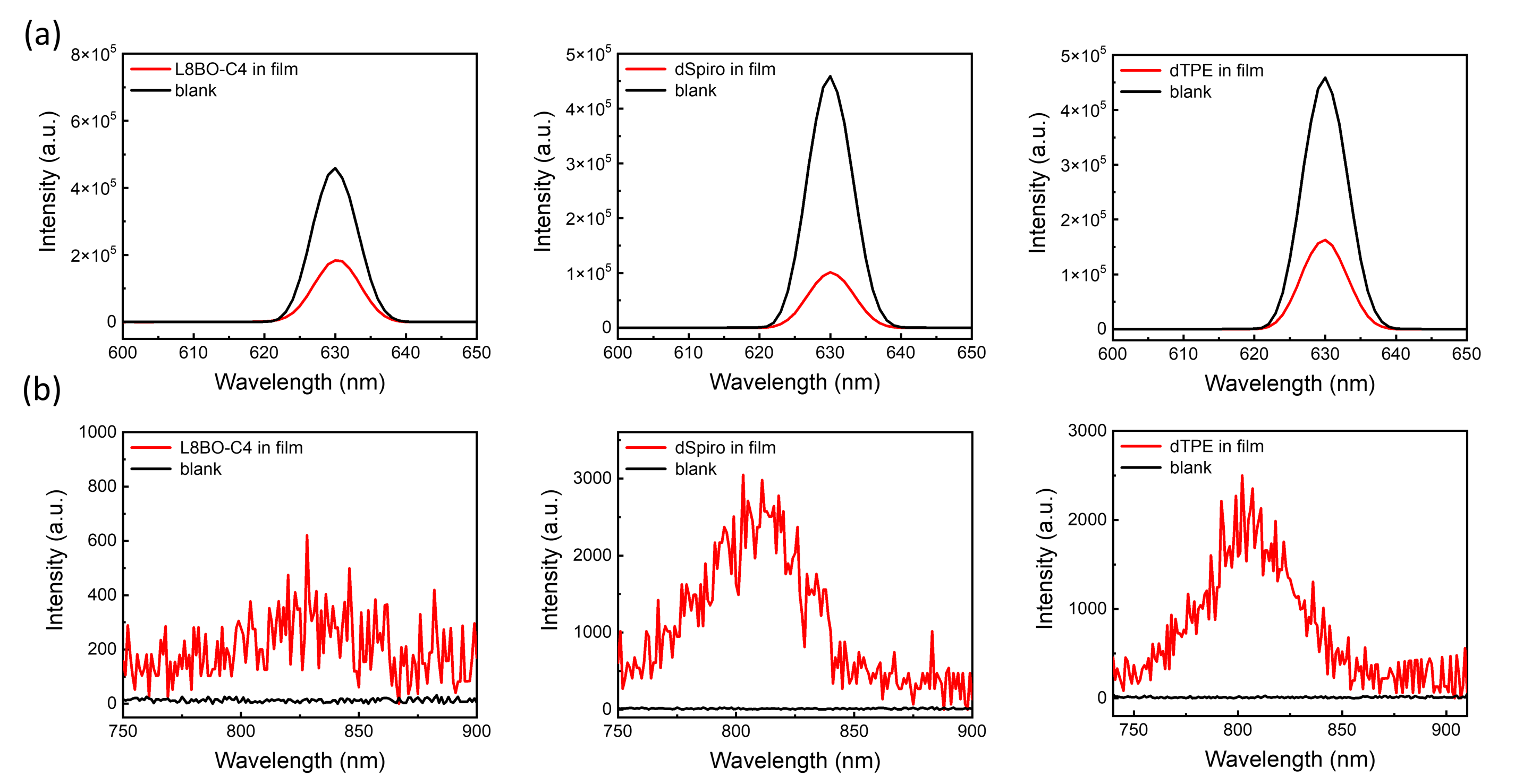


**Figure S7.** The (a) absorption and (b) emission spectra (excited at 630 nm, slit 5 nm) of L8BO-C4, dTPE and dSpiro in thin films.


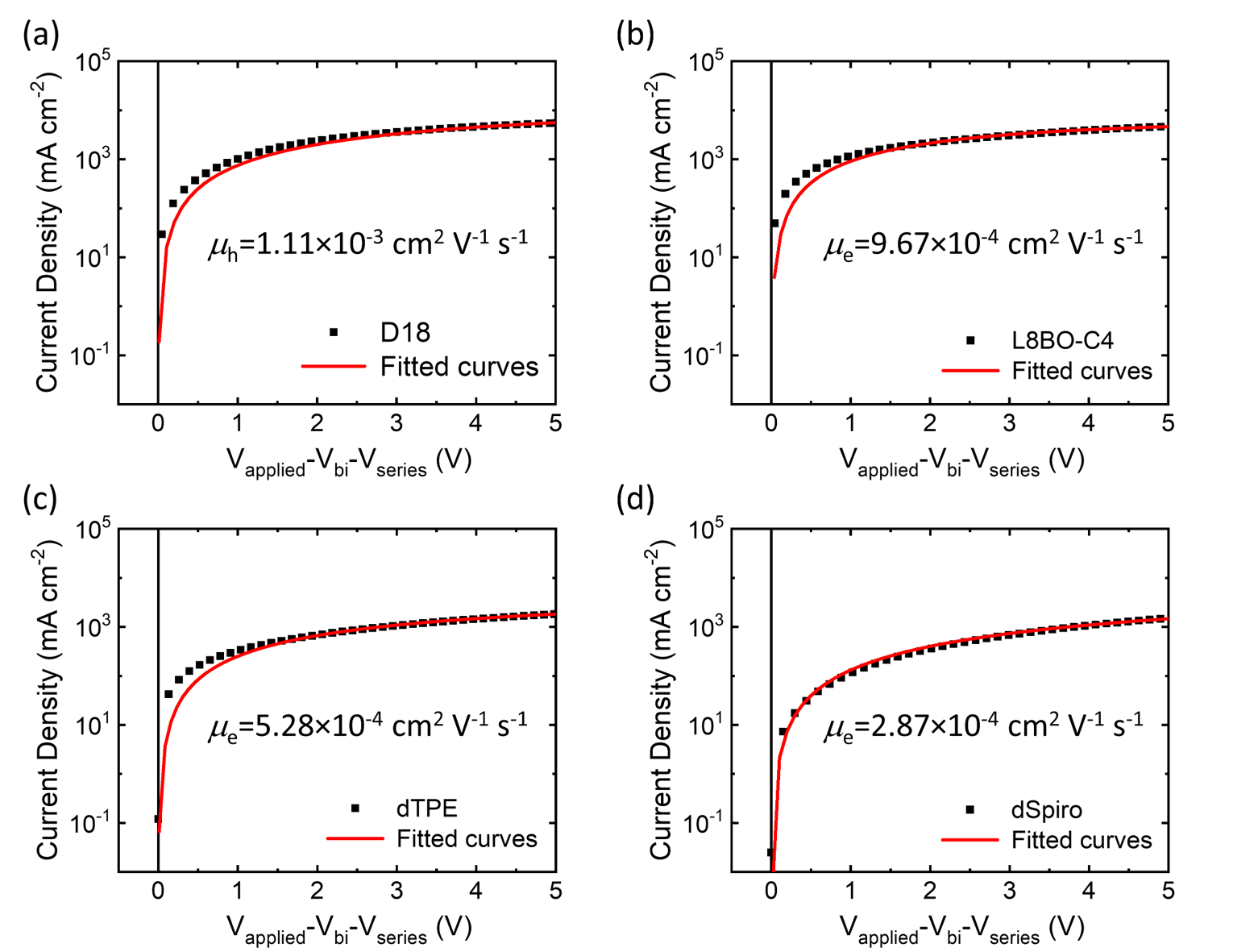


**Figure S8.** Space-charge-limited *J*-*V* plots from the hole-only device based on (a) D18 pure film and the electron-only device based on (b) L8BO-C4, (c) dTPE, and (d) dSpiro pure films.

1. **Photovoltaic performance of ternary devices**


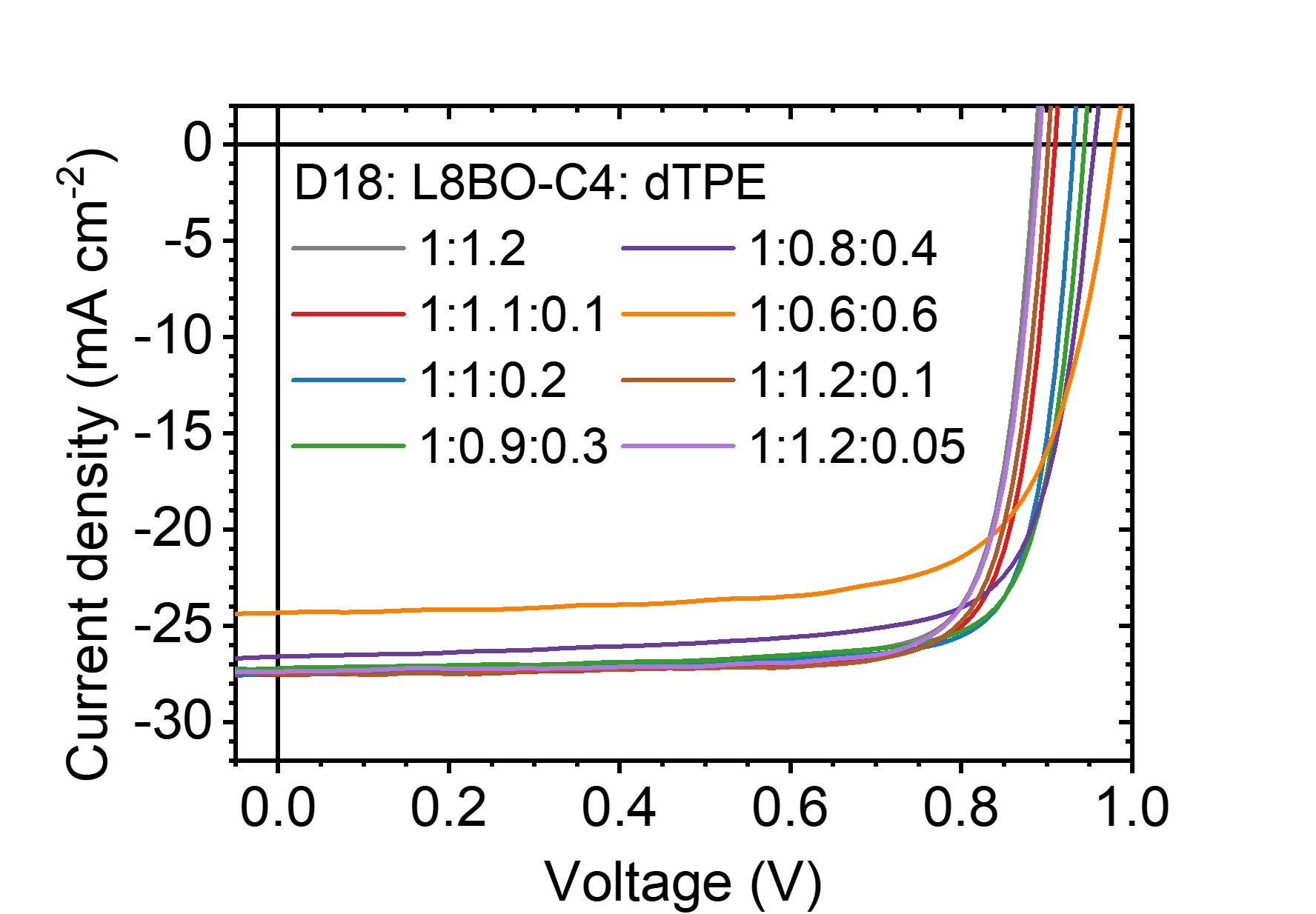


**Figure S9.** The *J*-*V* curves of ternary devices with different amounts of dTPE under simulated AM 1.5G irradiation (100 mW cm^-2^).

1. **SCLC measurements of blend films**


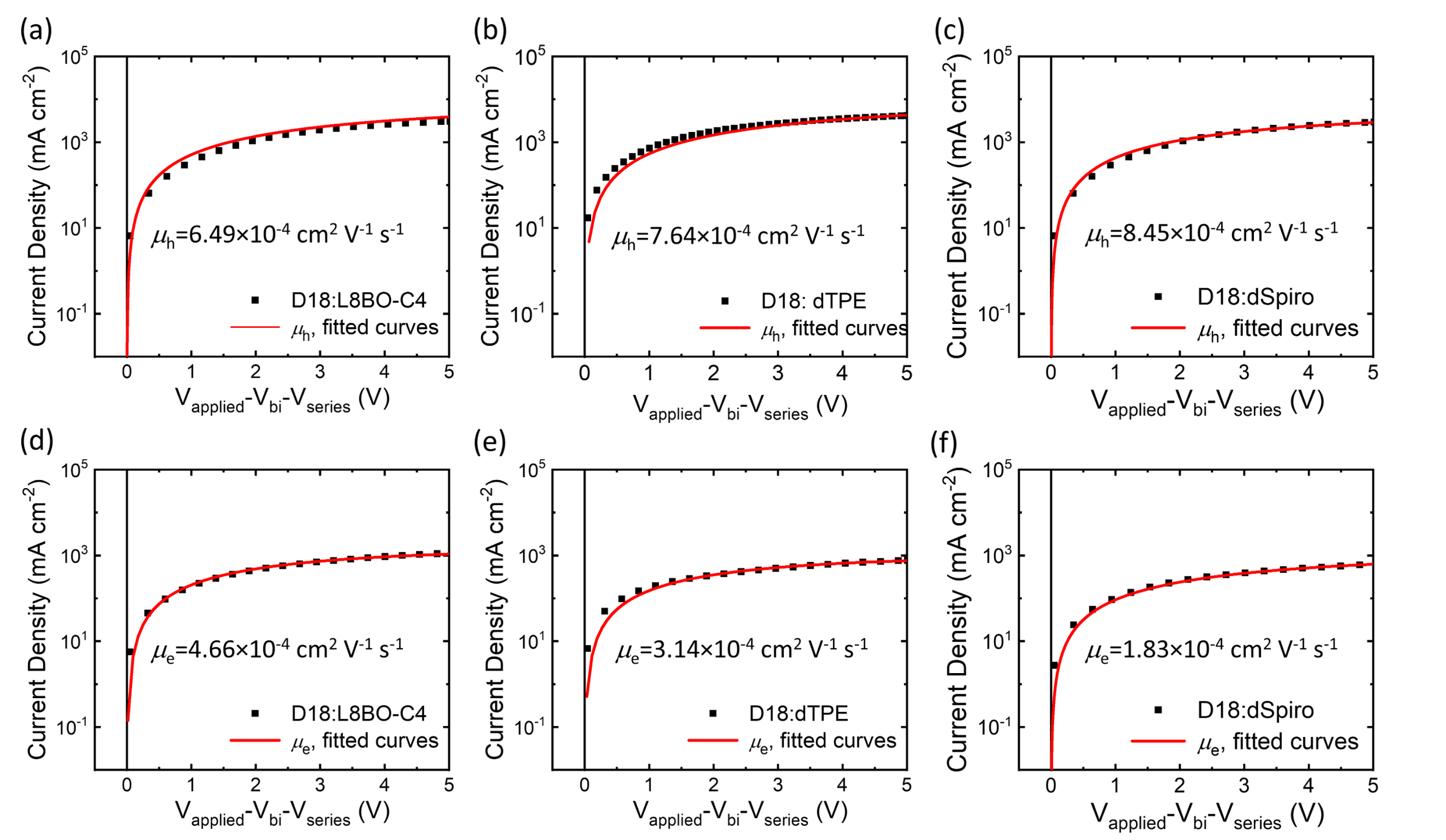


**Figure S10.** Space-charge-limited *J*-*V* plots from the hole-only device based on (a) D18:L8BO-C4, (b) D18:dTPE, and (c) D18:dSpiro binary blend film and the electron-only device based on (d) D18:L8BO-C4, (e) D18:dTPE, and (f) D18:dSpiro binary blend films.


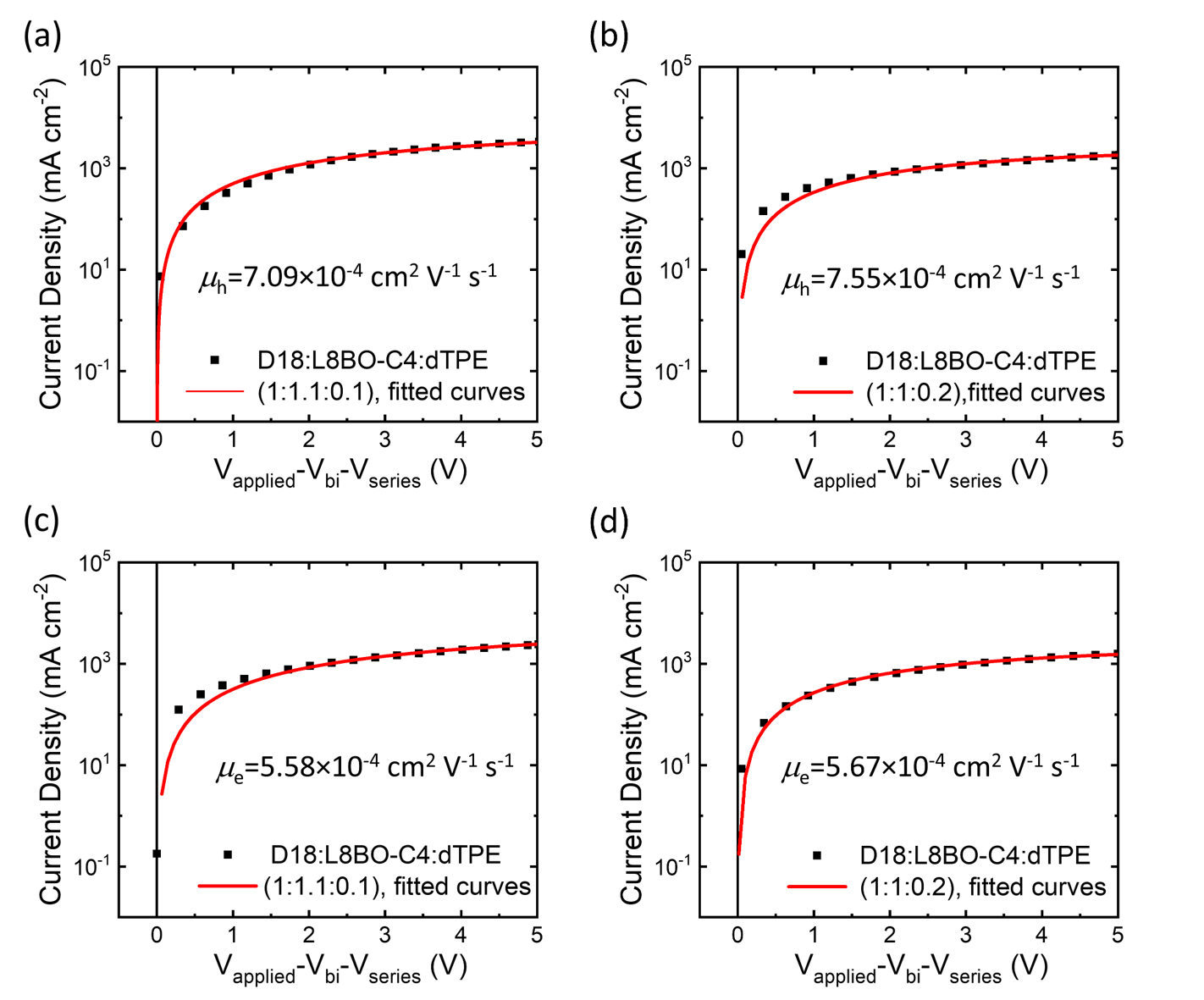


**Figure S11.** Space-charge-limited *J*-*V* plots from the hole-only device based on (a) D18:L8BO-C4:dTPE (1:1.1:0.1), and (b) D18:L8BO-C4:dTPE (1:1:0.2), and the electron-only device based on (c) D18:L8BO-C4:dTPE (1:1.1:0.1), and (d) D18:L8BO-C4:dTPE (1:1:0.2) ternary blend films.

1. **Light intensity dependence measurements**


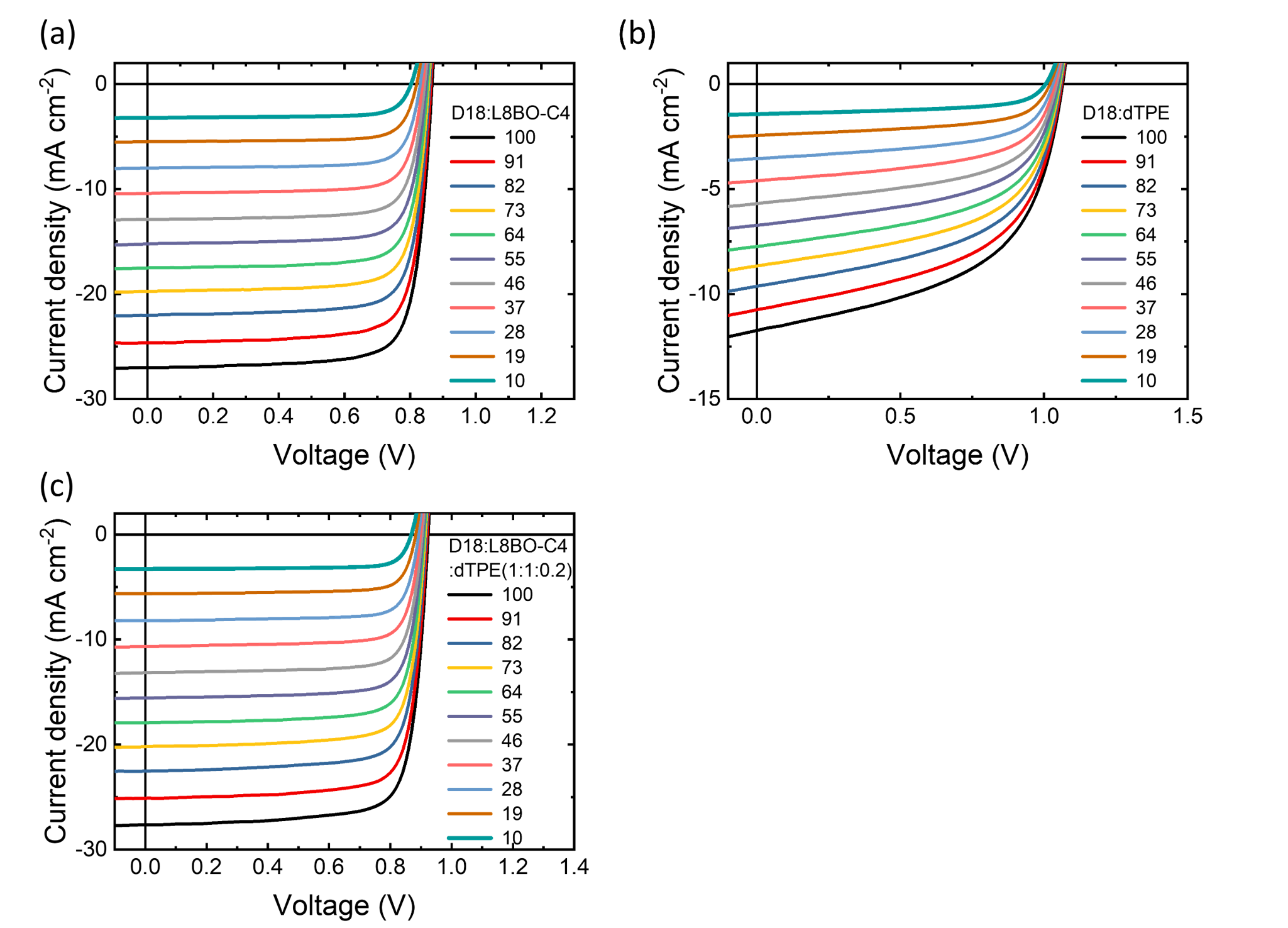


**Figure S12.** Characteristic *J*–*V* curves of (a) D18:L8BO-C4 devices, (b) D18: dTPE devices, and (c) D18:L8BO-C4:dTPE devices under different AM 1.5G illumination (10-100 mW cm^-2^).

1. **Energy loss measurements**


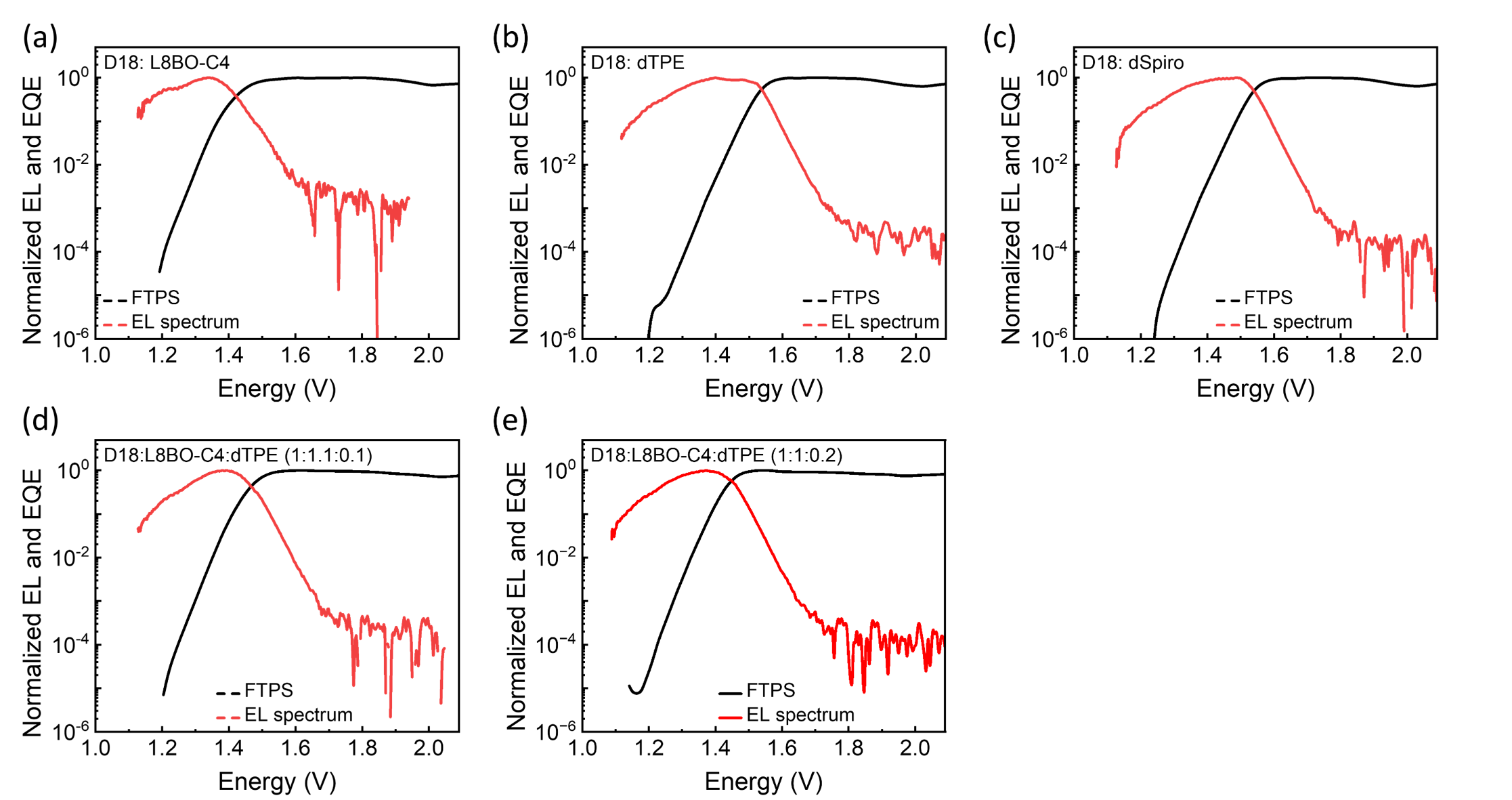


**Figure S13.** Normalized EL and normalized sEQE as a function of energy for OSCs based on (a) D18: L8BO-C4, (b) D18: dTPE, (c) D18: dSpiro, (d) D18: L8BO-C4: dTPE (1:1.1:0.1) and (e) D18: L8BO-C4: dTPE (1:1:0.2) devices.


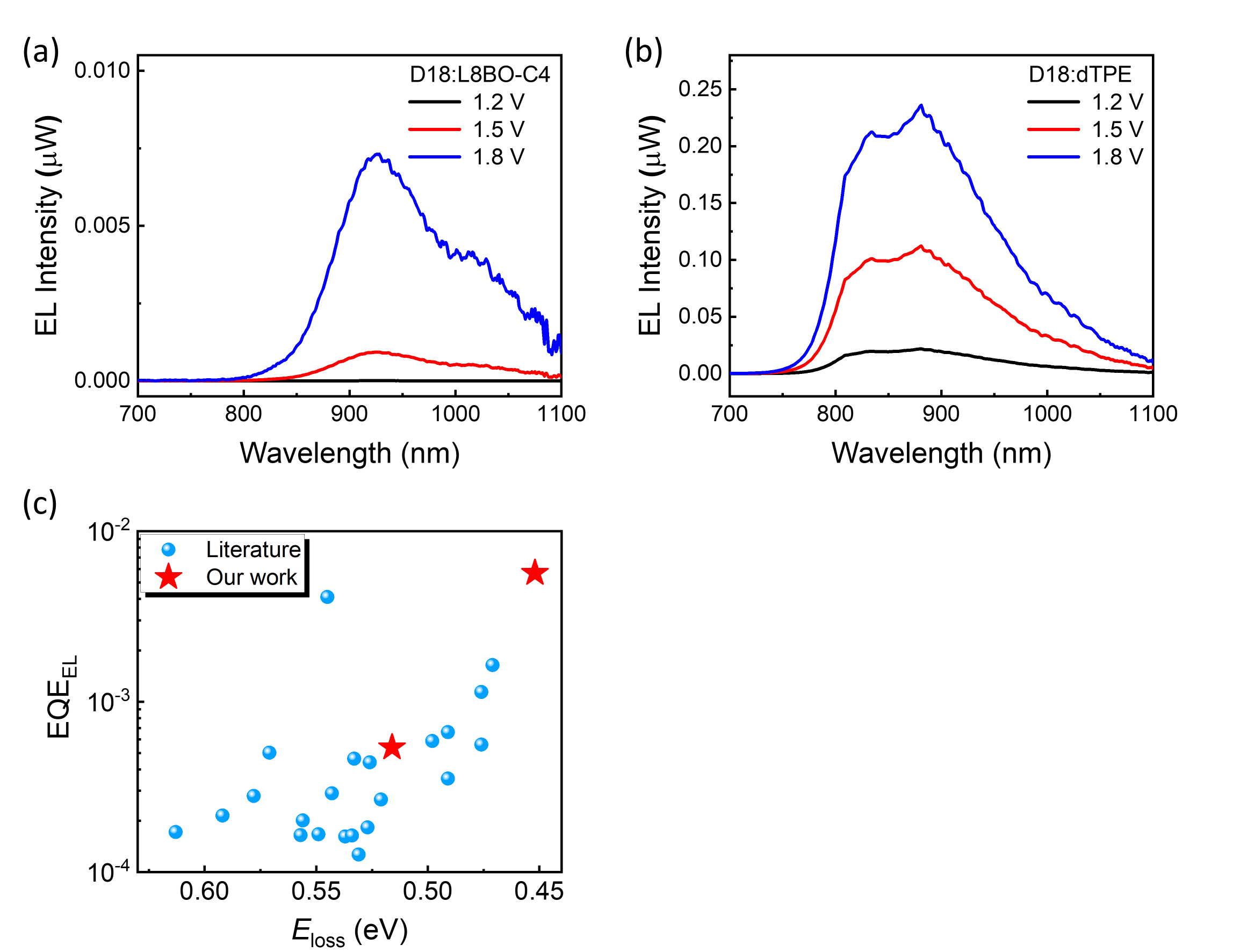


**Figure S14.** The EL spectra of (a) D18: L8BO-C4-based and (b) D18: dTPE-based devices under different bias voltage. (c) Plots of the EQE_EL_ versus E_loss_ of the OSCs reported in the literatures

1. **GIWAXS measurements for pure films**


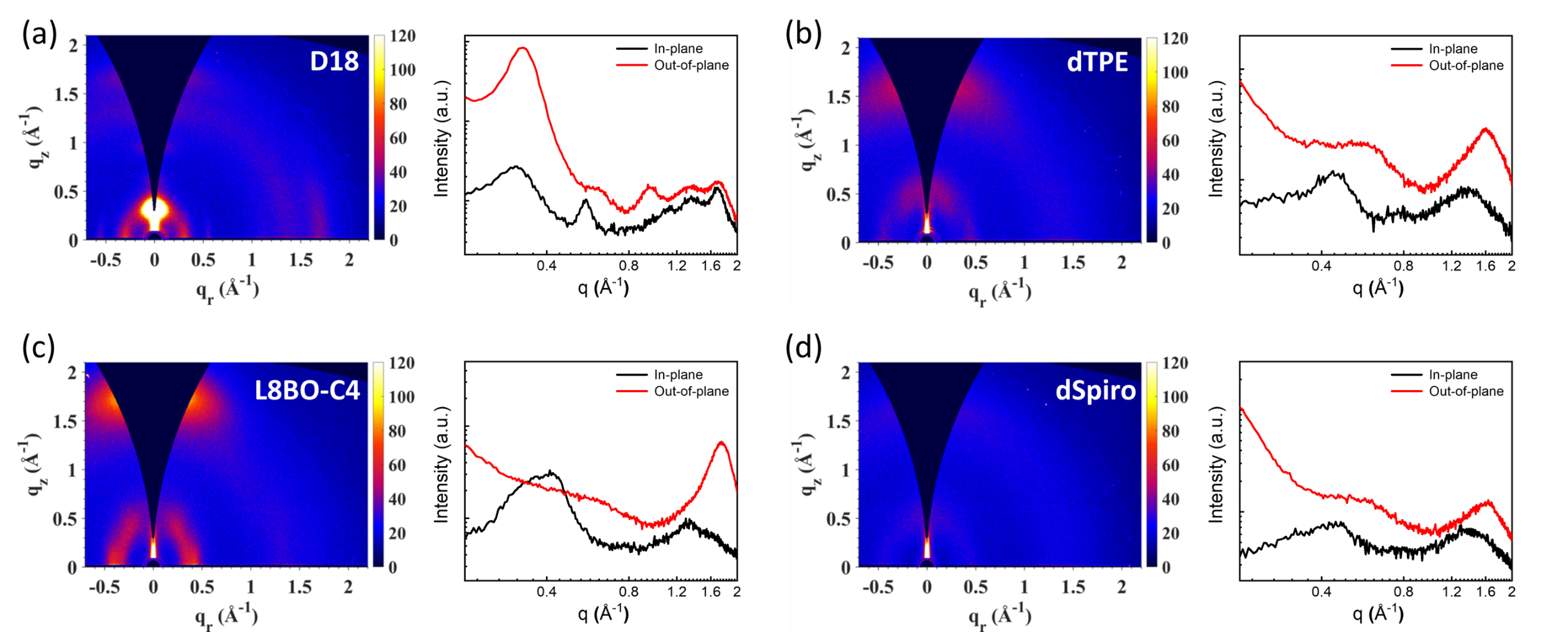


**Figure S15.** The 2D GIWAXS profiles and corresponding 1D line-cut curves along OOP and IP directions of (a) D18, (b) dTPE, (c) L8BO-C4, and (d) dSpiro pure films.

1. **Molecular dynamics simulation**


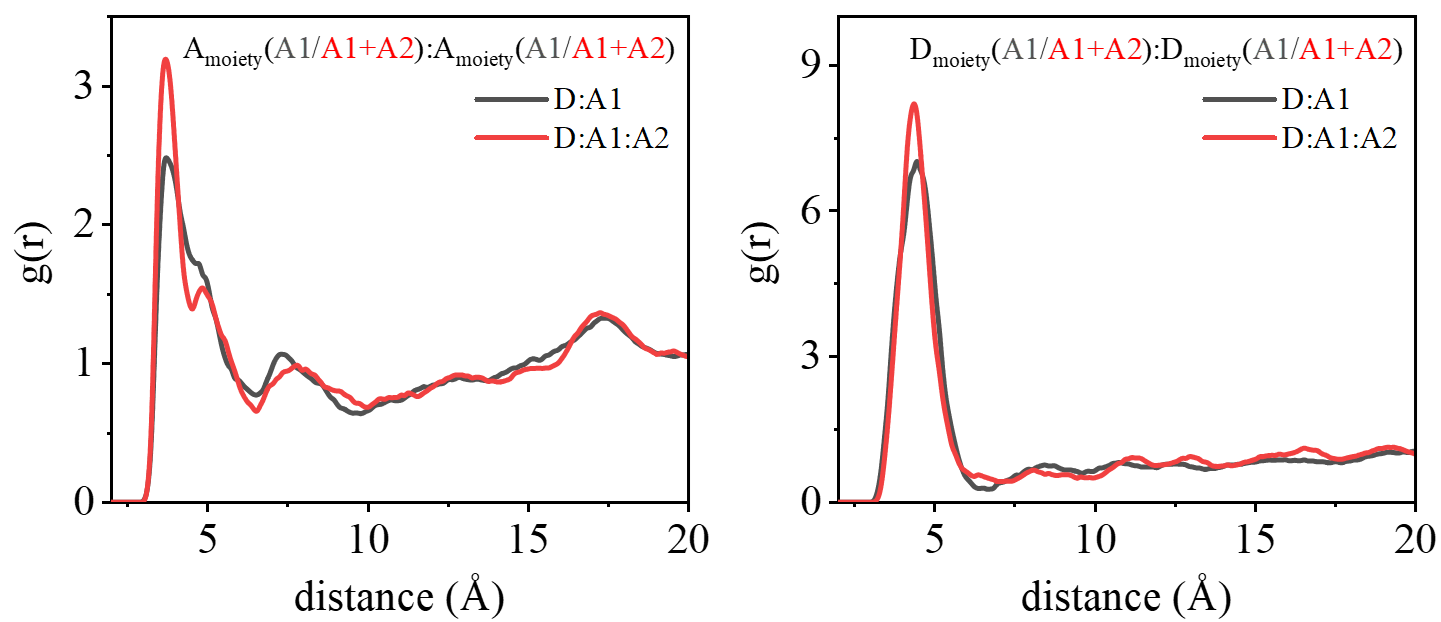


**Figure S16**. g(r) between the A_moiety_ of acceptor and between the D_moiety_ of acceptor in binary (black lines) and ternary (red lines) blends.


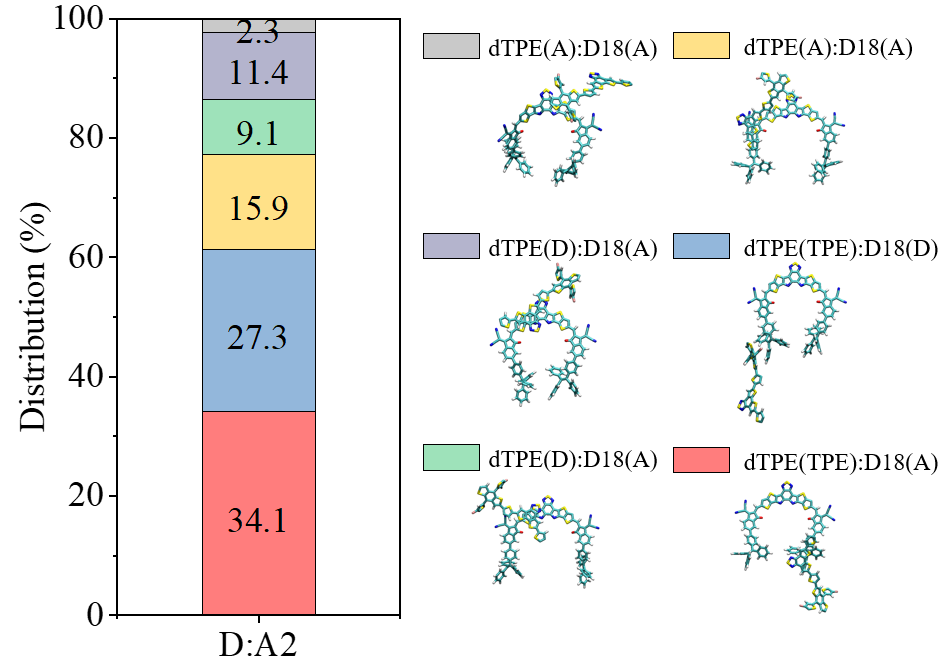


**Figure S17.** Distribution of the prevalent D:A2 stacking configurations within the ternary blend.


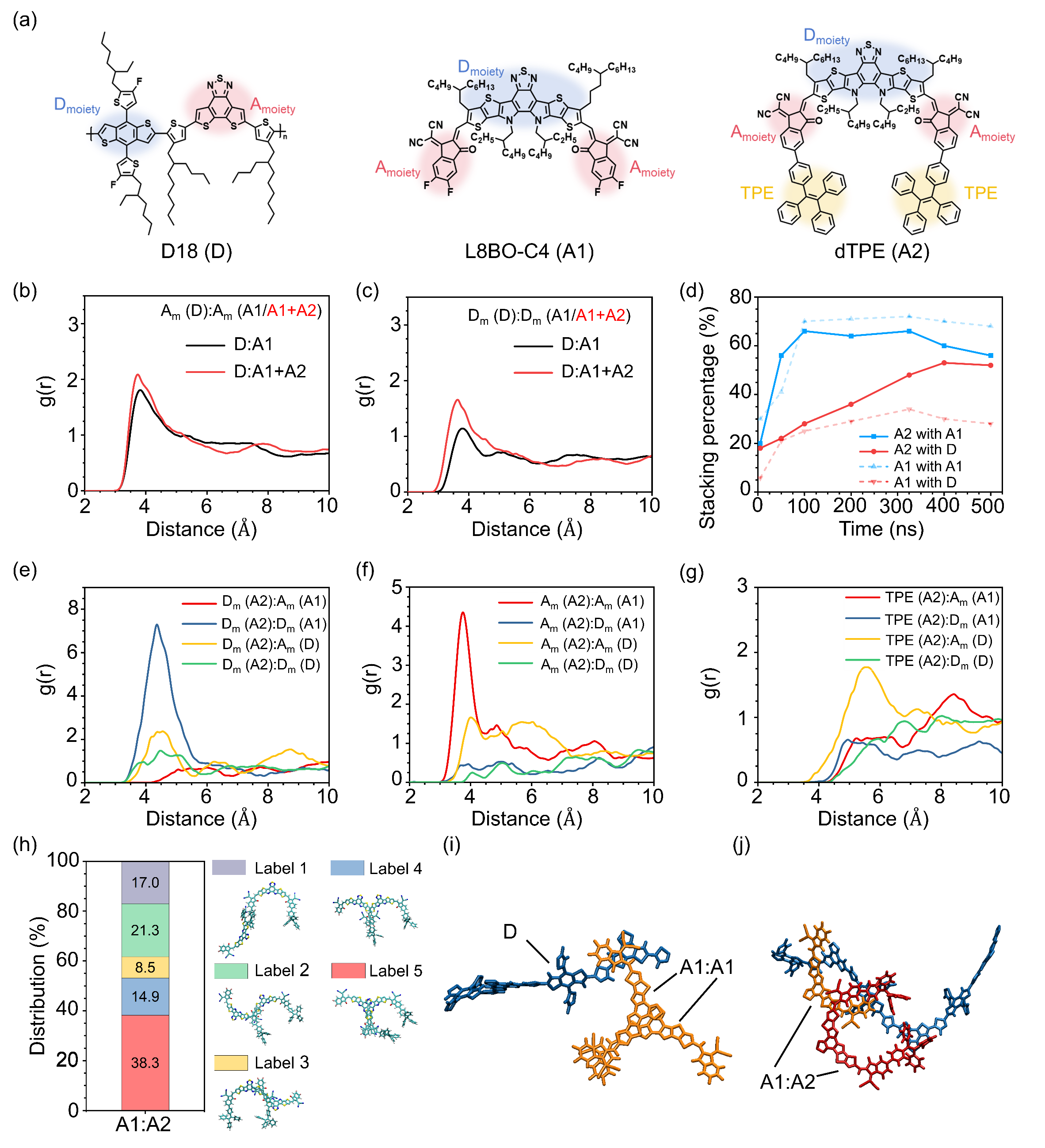


**Figure S18.** (a) The donor moiety (*D*_moiety_), acceptor moiety (*A*_moiety_) and TPE group of D18 (denoted as D) , L8-BO-C4 (denoted as A1) and dTPE (denoted as A2). (b,c) g(r) between (b) the Amoiety of donor and the Amoiety of acceptor, and between (c) the Dmoiety of donor and the Dmoiety of acceptor in binary (black lines) and ternary (red lines) blends. The A/Dmoiety of acceptor is denoted as A/Dmoiety(A1) in binary blend and is denoted as A/Dmoiety(A1+A2) in ternary blend. (d) Time evolution of the molecular stacking percentage during the film formation simulation. The solid lines represent the percentage of A2 that is stacked with A1 and D molecules, respectively. The dashed lines represent the percentage of A1 that is stacked with A1 and D molecules, respectively. (e-g) g(r) between the (e) Dmoiety, (f) Amoiety, and (g) TPE group of the A2 molecule with moieties of the D and A1 molecules. (h) Distribution of the prevalent A1:A2 stacking configurations within the ternary blend. (i-j) Snapshots of the interfacial molecular stacking configurations extracted from the MD simulations of the (i) binary and (g) ternary blends.

1. **^1^H NMR and MS spectra**


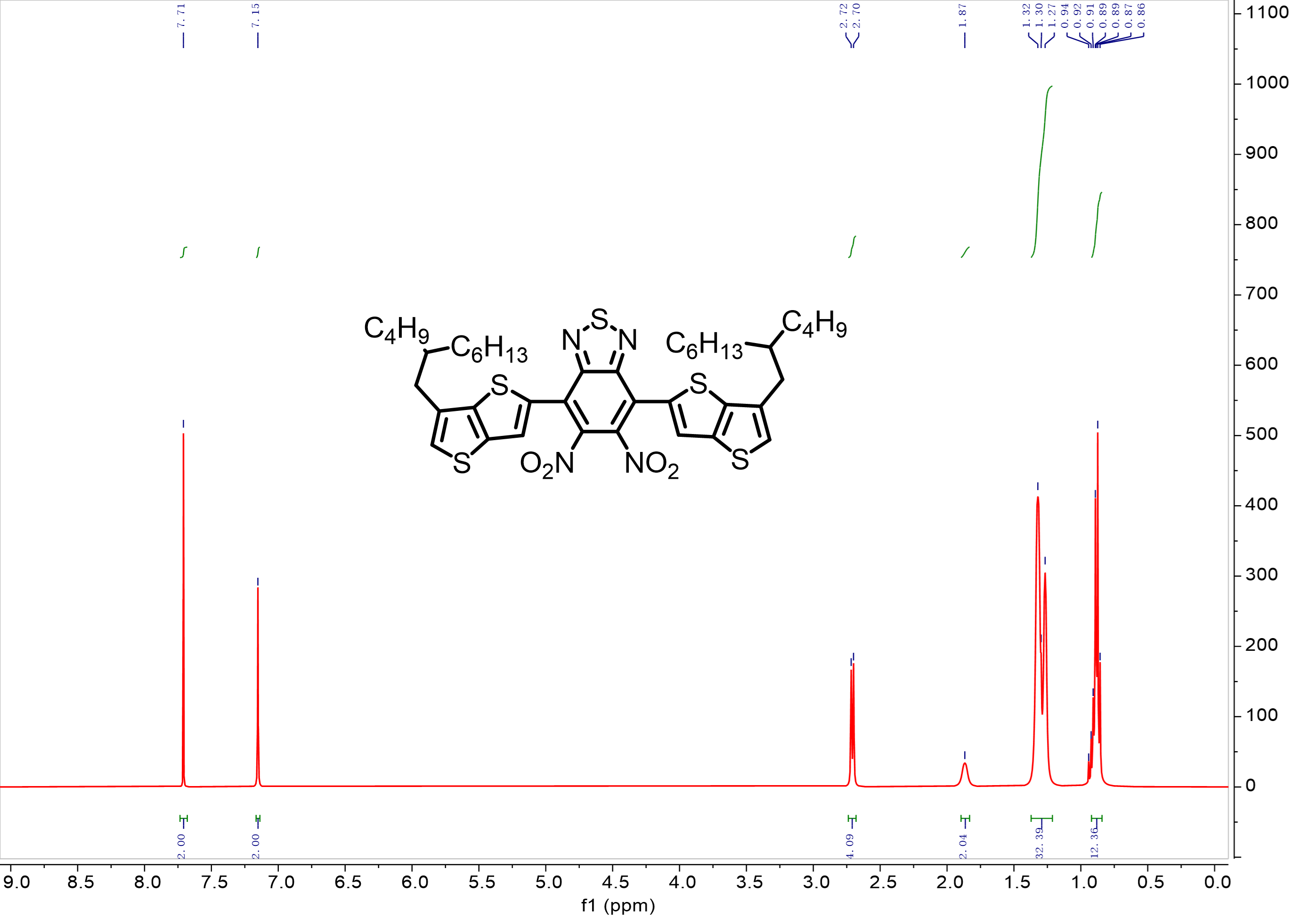


**Figure S19.** ^1^H NMR of compound **4**.


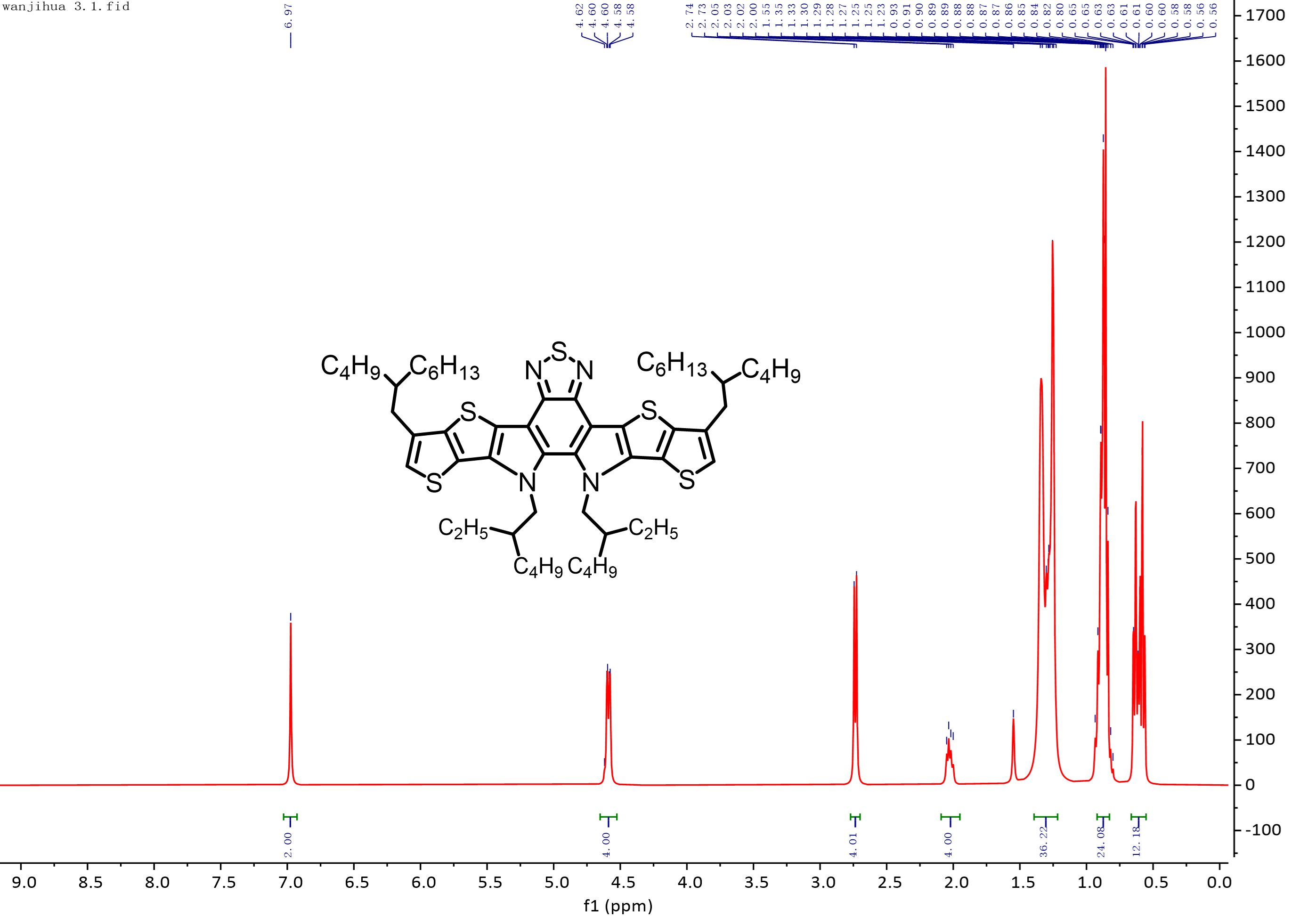


**Figure S20.** ^1^H NMR of compound **5**.


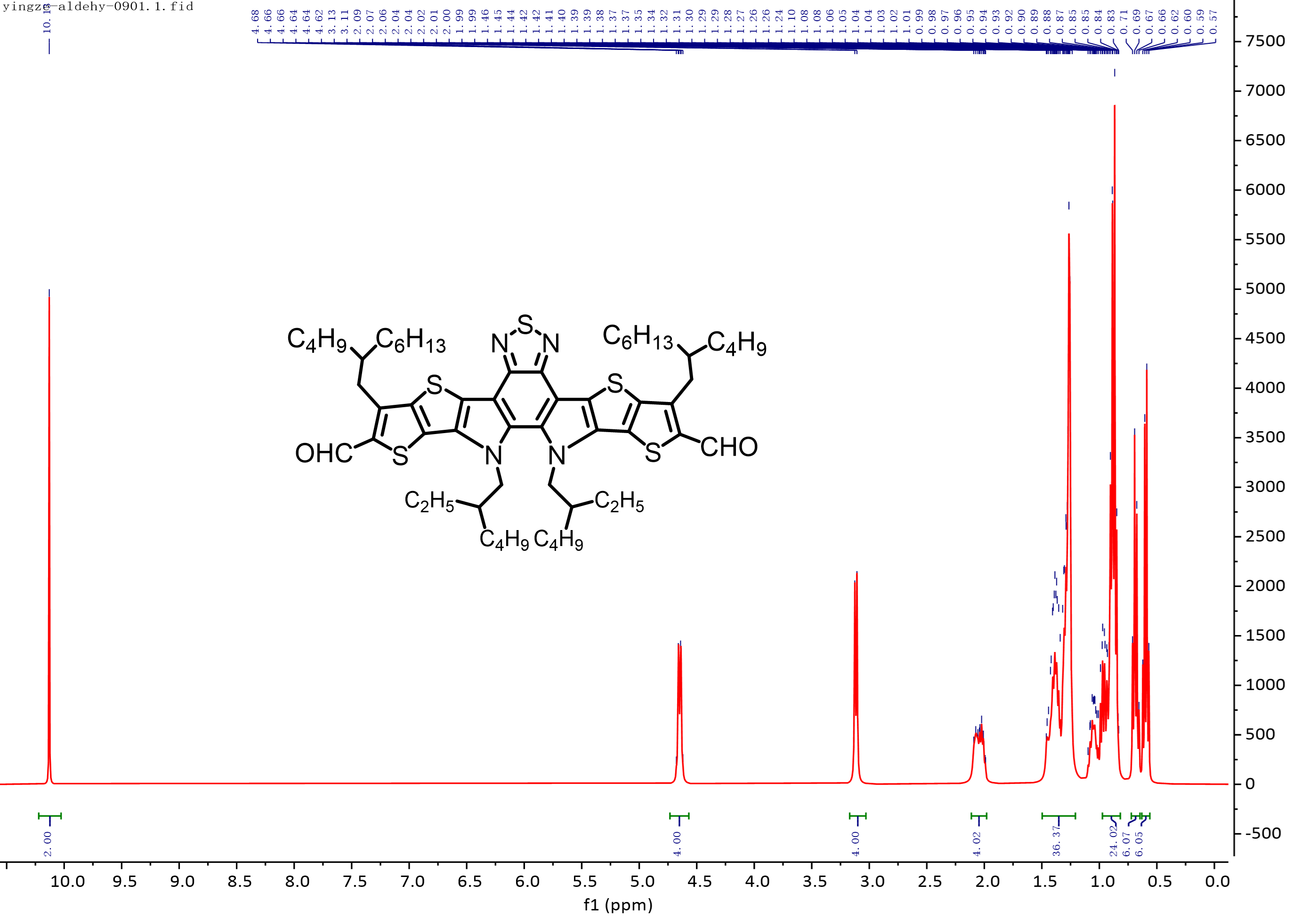


**Figure S21.** ^1^H NMR of compound **6**.


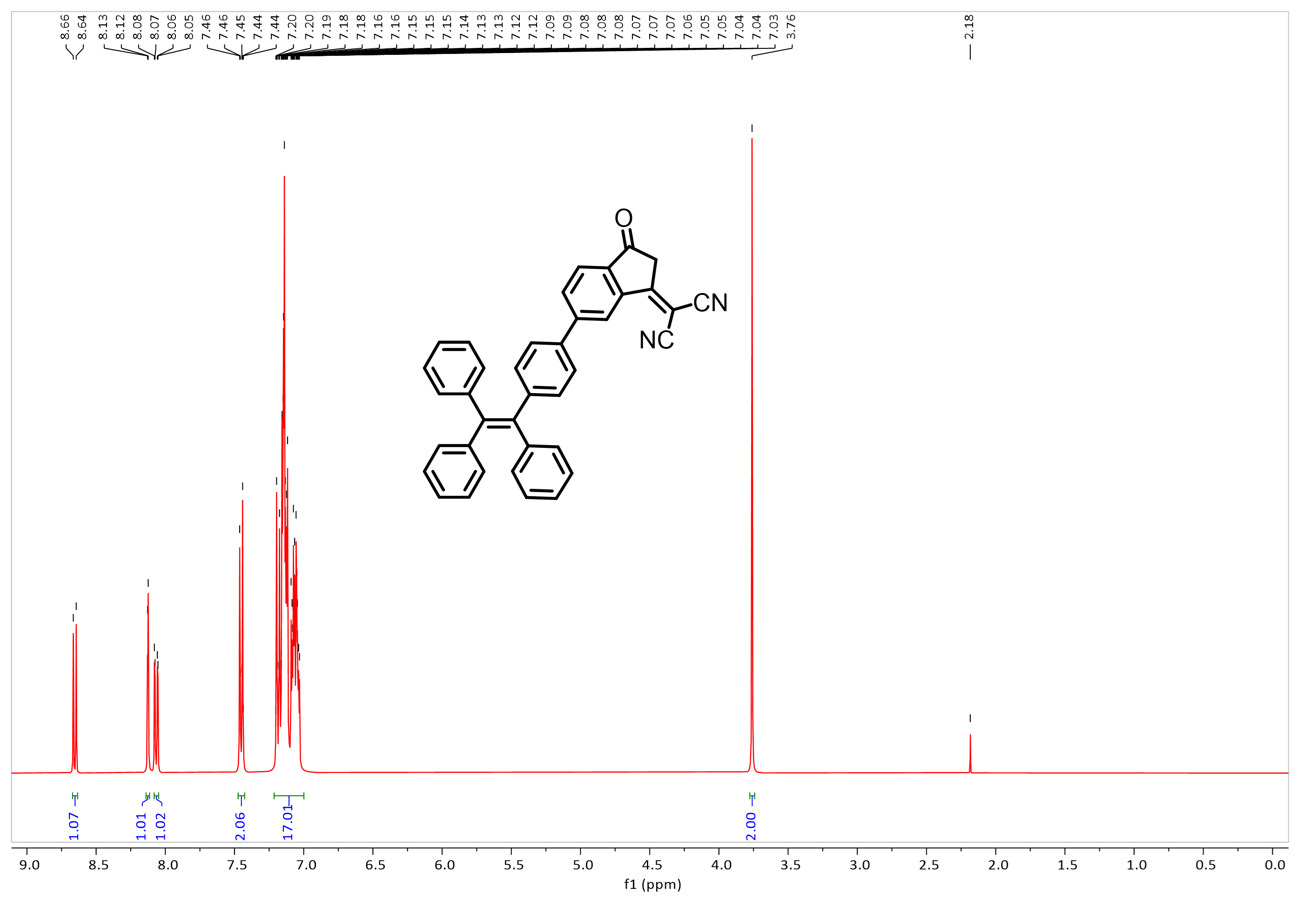


**Figure S22.** ^1^H NMR of compound **2** (IC-TPE).


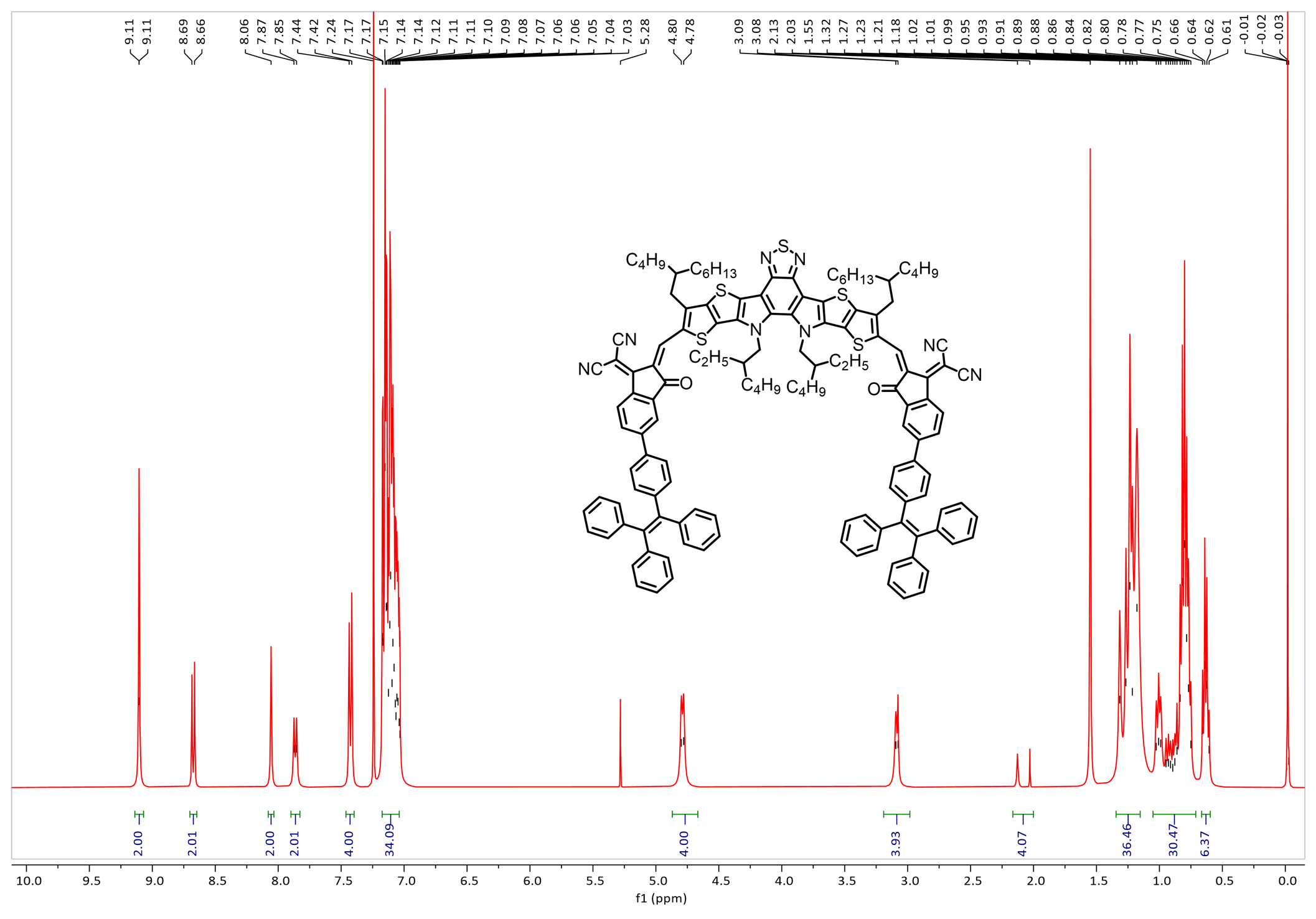


**Figure S23.** ^1^H NMR of dTPE.


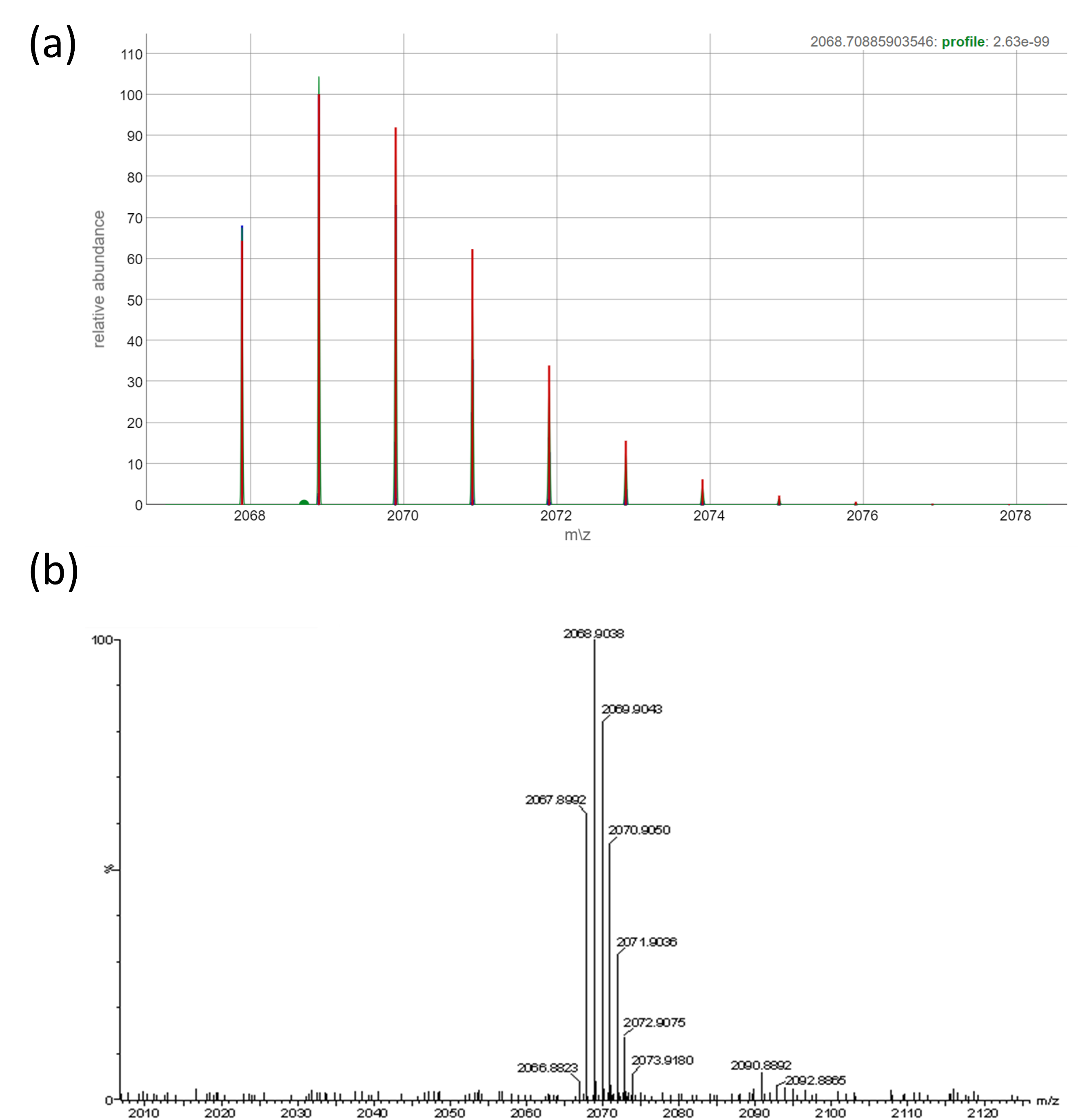


**Figure S24.** (a) Simulated isotopic patterns for the [M+H]^+^ ion and (b) the high-resolution MALDI-TOF MS spectra of dTPE.


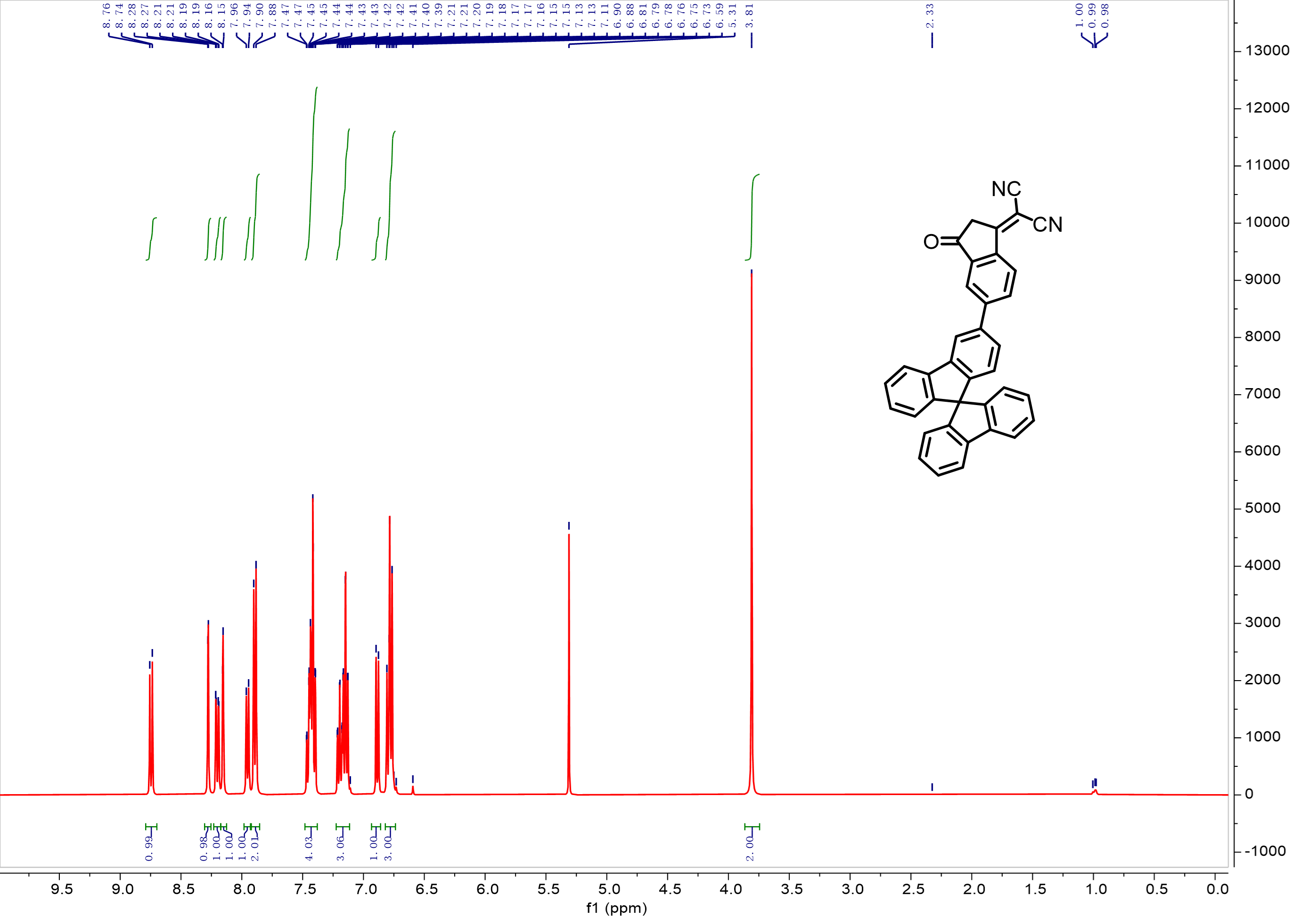


**Figure S25.** ^1^H NMR of compound **7**.


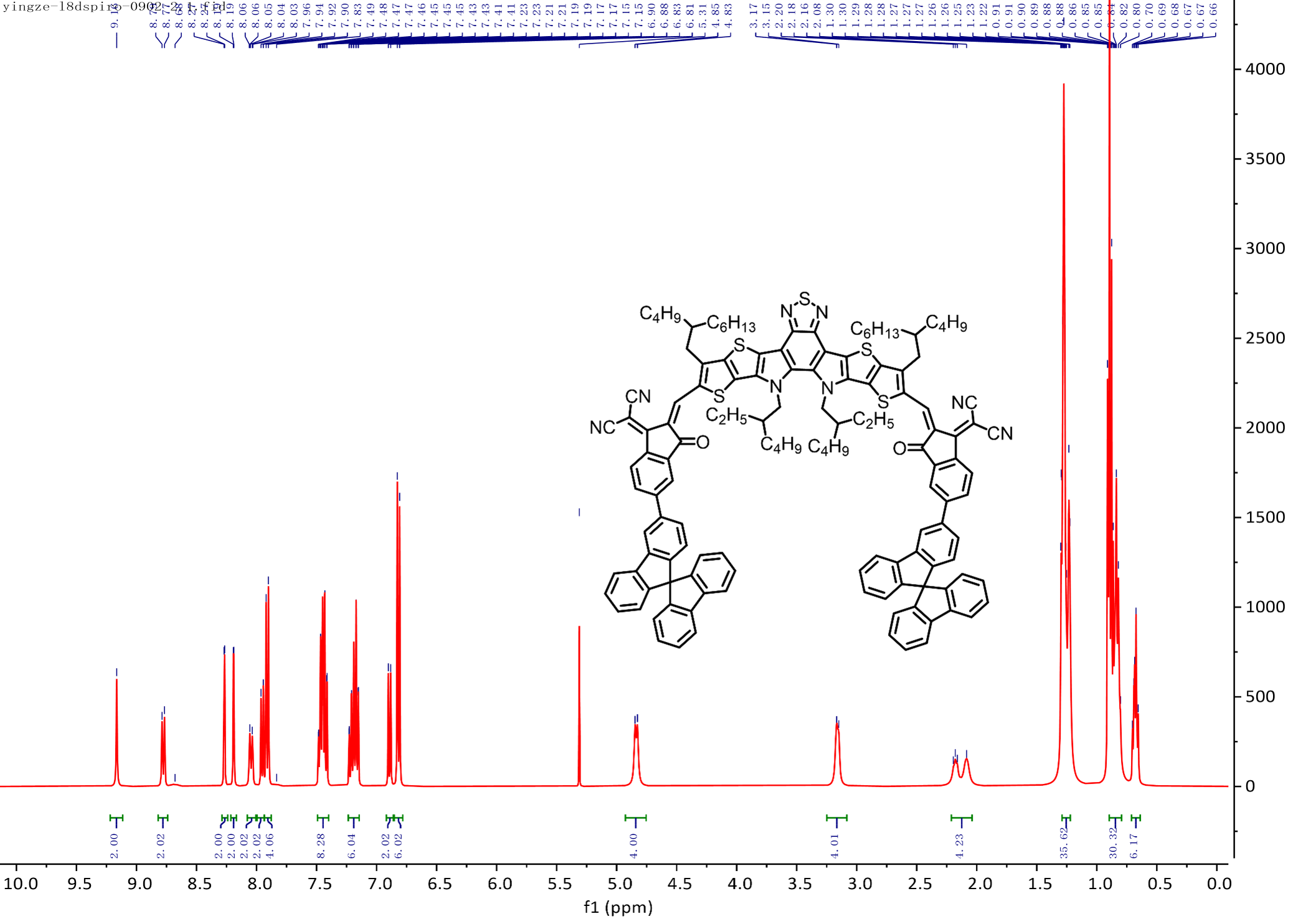


**Figure S26.** ^1^H NMR of dSpiro.

**
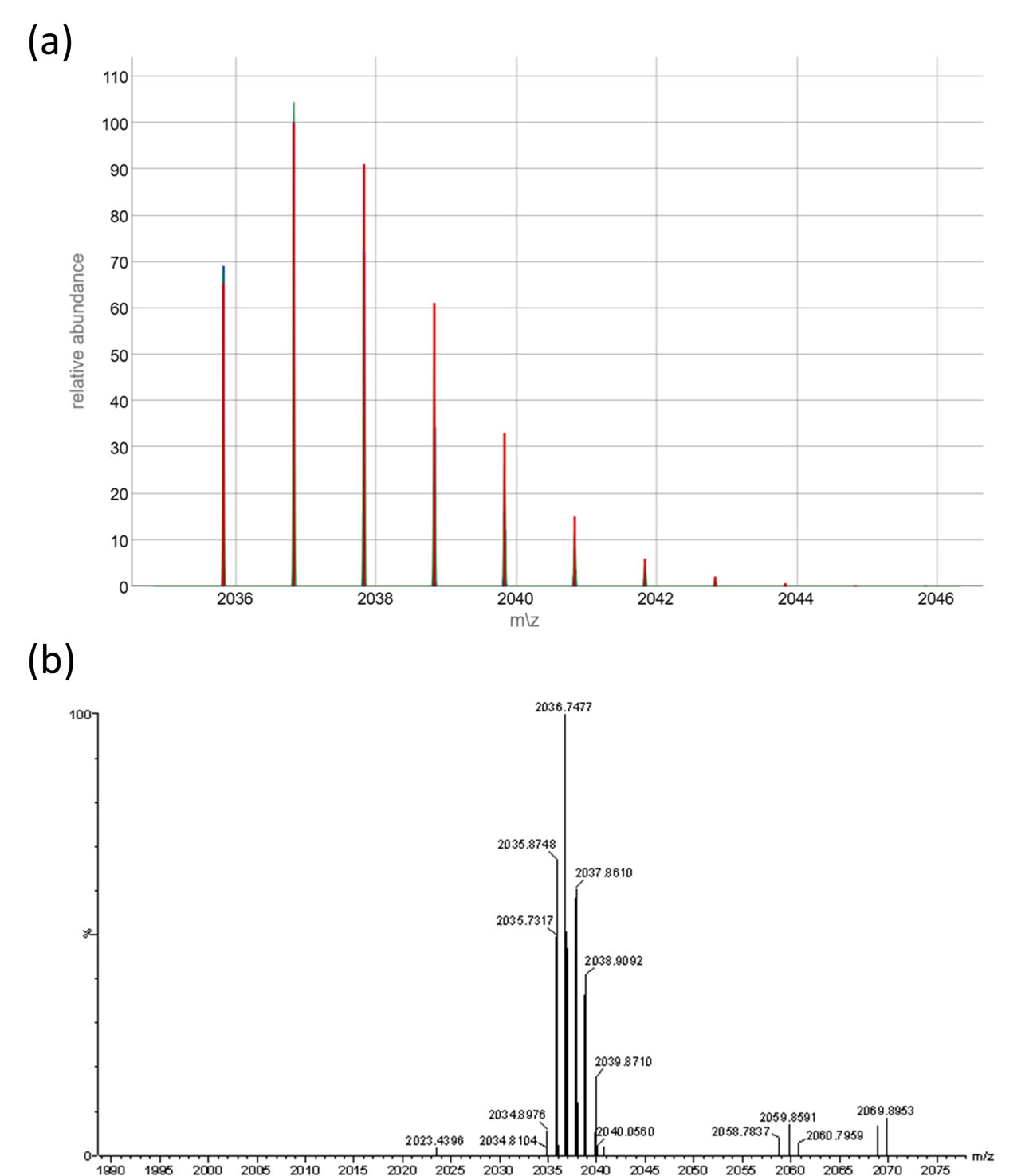
**

**Figure S27.** (a) Simulated isotopic patterns for the [M+H]^+^ ion and (b) the high-resolution MALDI-TOF MS spectra of dSpiro.


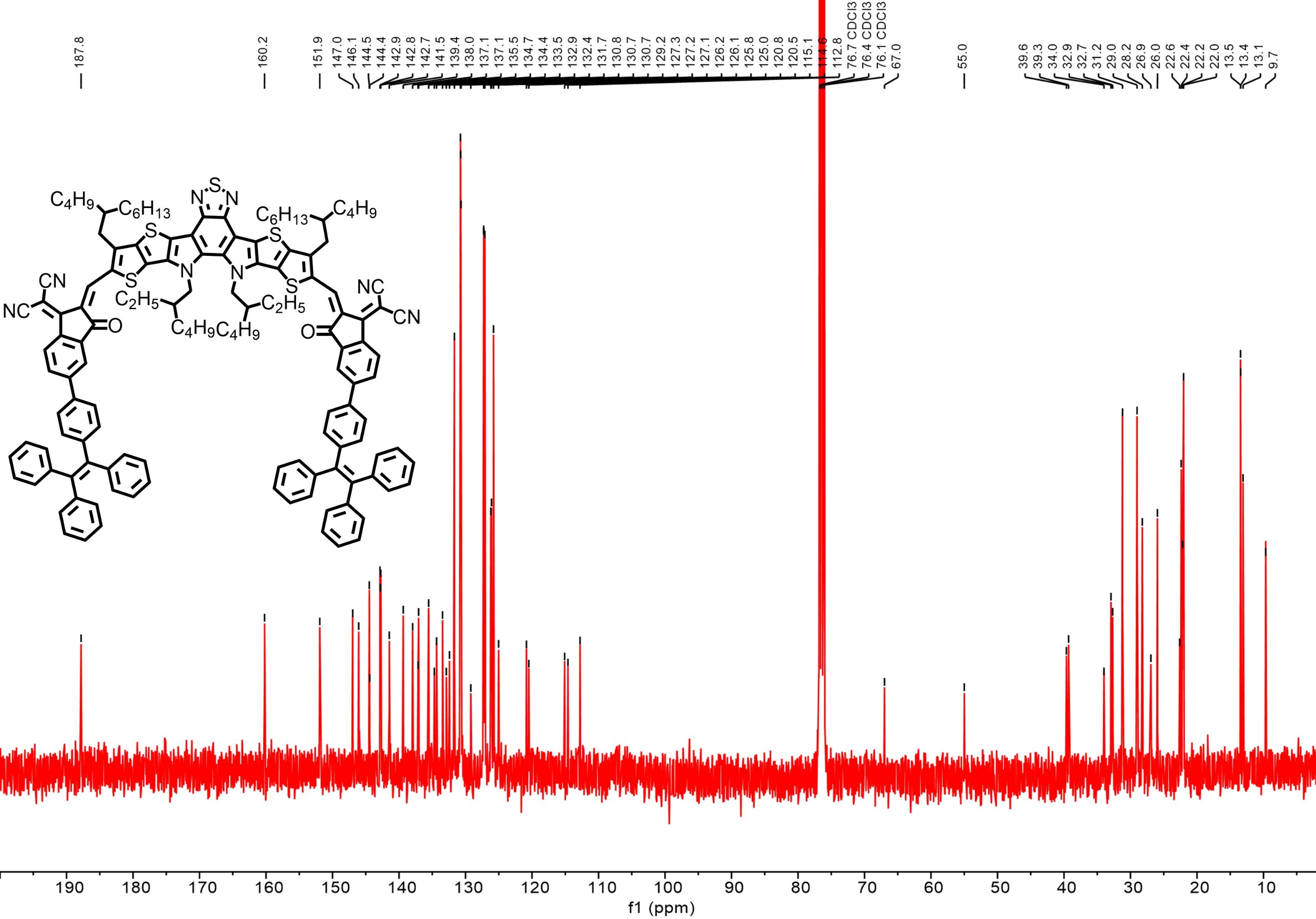


**Figure S28.** ^13^C NMR of dTPE.

1. **Light Stability Measurements**

**
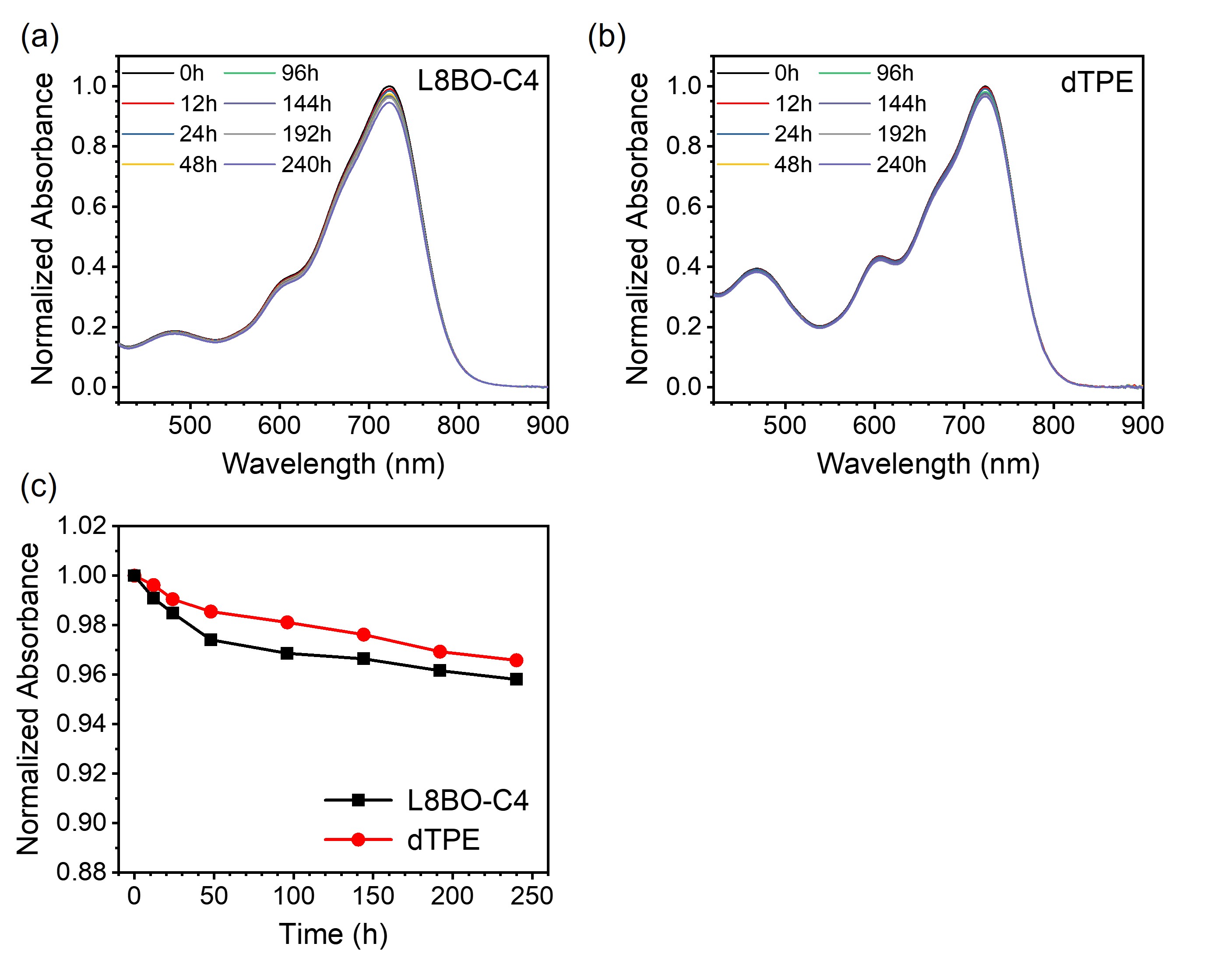
**

**Figure S29.** The absorption spectra of (a) L8BO-C4 and (b) dTPE choloform solution under natural light illumination for different time, (c) the decay curves of solutions at characteristic absorption peak.


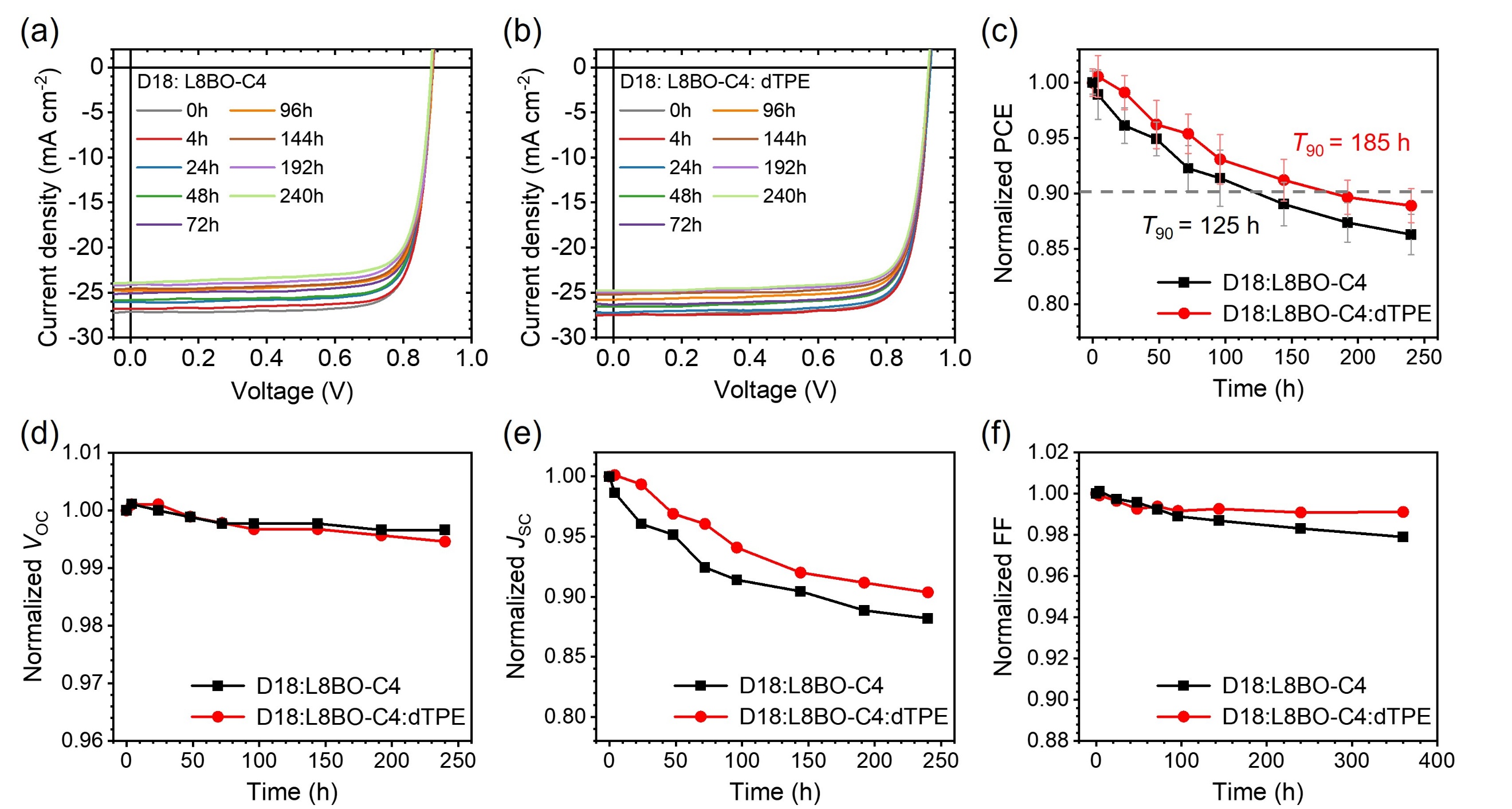


**Figure S30.** The characteristic *J*-*V* curves of (a) D18:L8BO-C4 and (b) D18:L8BO-C4:dTPE devices under different light storing time. The normalized (c) PCE, (d) *V*_OC_, (e) *J*_SC_, and (f) FF decay curves of the D18:L8BO-C4 and D18:L8BO-C4:dTPE devices under continuous 100 mW cm^-2^ illumination provided by LED-solar simulators in glove box. The error bars were calculated from five individual devices.

In order to further evaluate the application potential our strategy, the stability tests and the universality measurements were performed. The light stability of materials in chloroform was characterized by absorption spectra. As shown in Figure S29, after 240 hours natural light illumination, the peak absorption of L8BO-C4 decreased by 5.3%, while dTPE only decreased by 3.5%, indicating its superior light stabiltiy. Subsequently, the light stability of the binary and ternary devices was conducted under continuous illumination of simulated solar illumination. As shown in Figure S30, the *T*_90_ values for the PCE of D18:L8BO-C4 and D18:L8BO-C4:dTPE were calculated to be 125 h and 185 h, respectively, demonstrating that dTPE, as a guest acceptor, can improve the light stability of corresponding OSC devices. The thermal stability of devices was also tests under continuous annealing at 85°C. As shown in Figure S32, the *T*_85_ lifetimes were 130 h and 225 h for the binary and ternary devices, separately, indicating that the incorporation of dTPE enhances the thermal stability of OSCs. The detailed performance results are summarized in Tables S8 and S9.

1. **Thermal Stability Measurements**

**
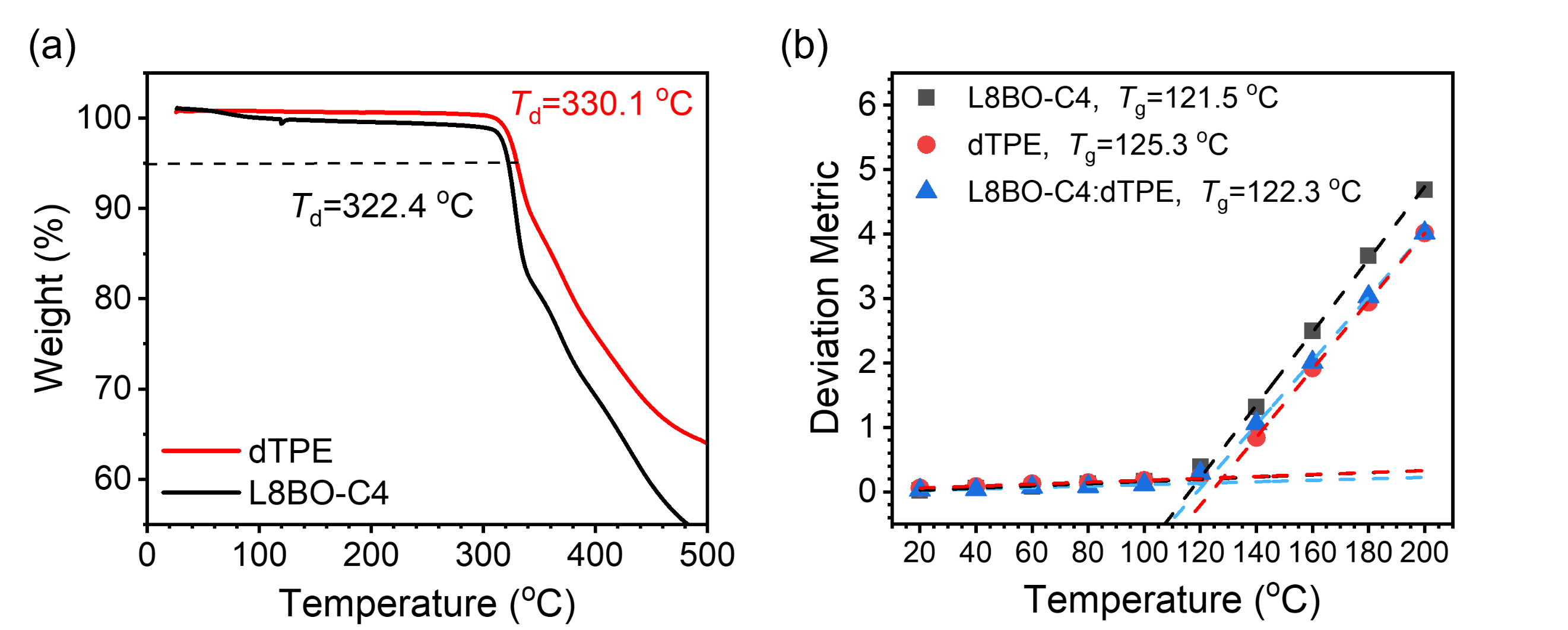
**

**Figure S31.** The (a) TGA curve of dTPE and L8BO-C4 under N_2_ atmosphere at a scan rate of 10 ^o^C min^−1^ . The *T*_d_ of dTPE and L8BO-C4 are 330.1 ^o^C and 322.4 ^o^C, respectively. (b) Plots of the DMT of L8BO-C4, dTPE, and L8BO-C4:dTPE films as a function of annealing temperature.


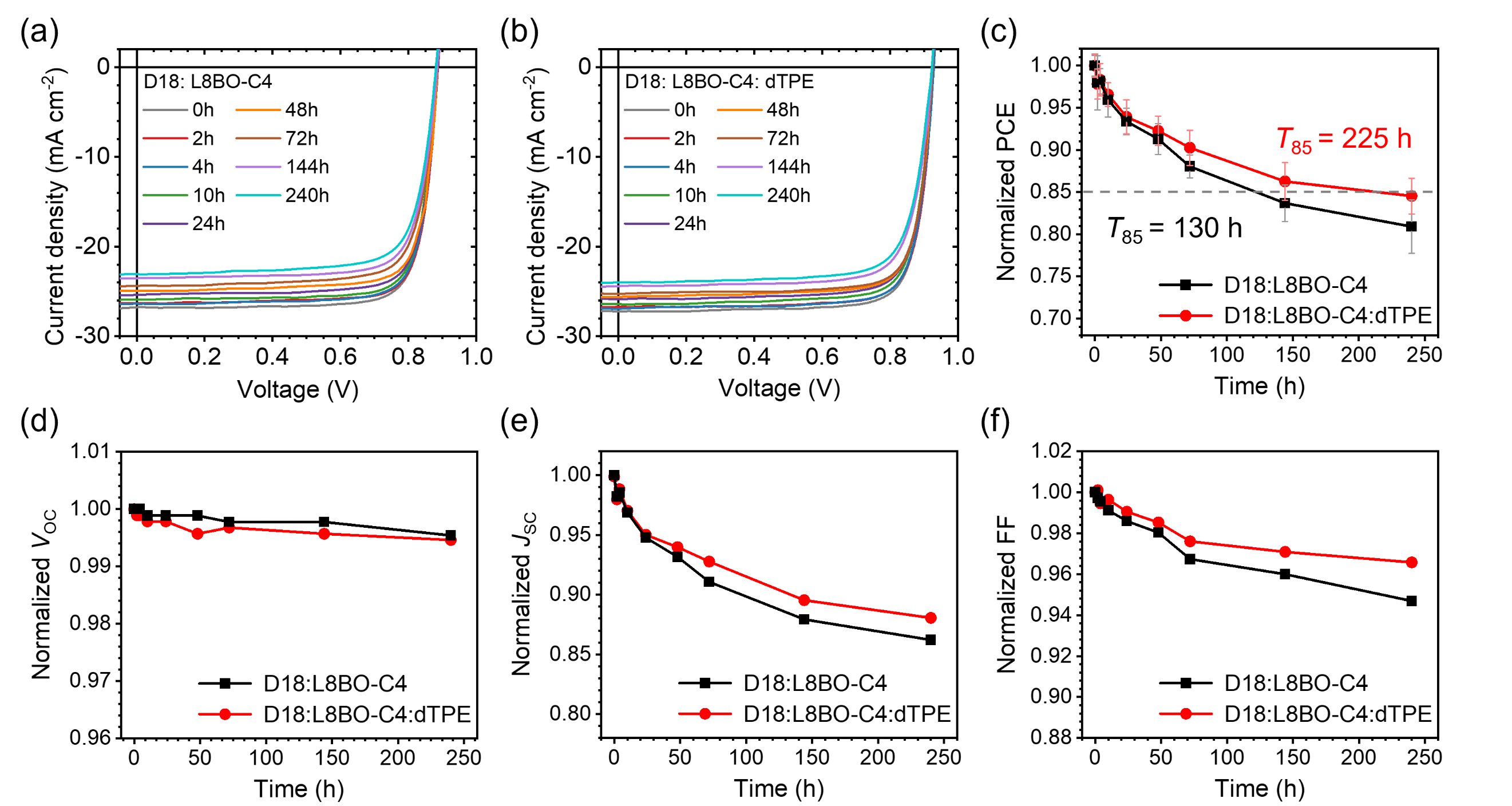


**Figure S32.** The characteristic *J*-*V* curves of (a) D18:L8BO-C4 and (b) D18:L8BO-C4:dTPE devices under different annealing time. (c) The normalized (c) PCE, (d) *V*_OC_, (e) *J*_SC_, and (f) FF decay curves of the D18:L8BO-C4 and D18:L8BO-C4:dTPE devices under 85 ^o^C continuous annealing in glove box. The error bars were calculated from six individual devices.

1. **Universality**

**
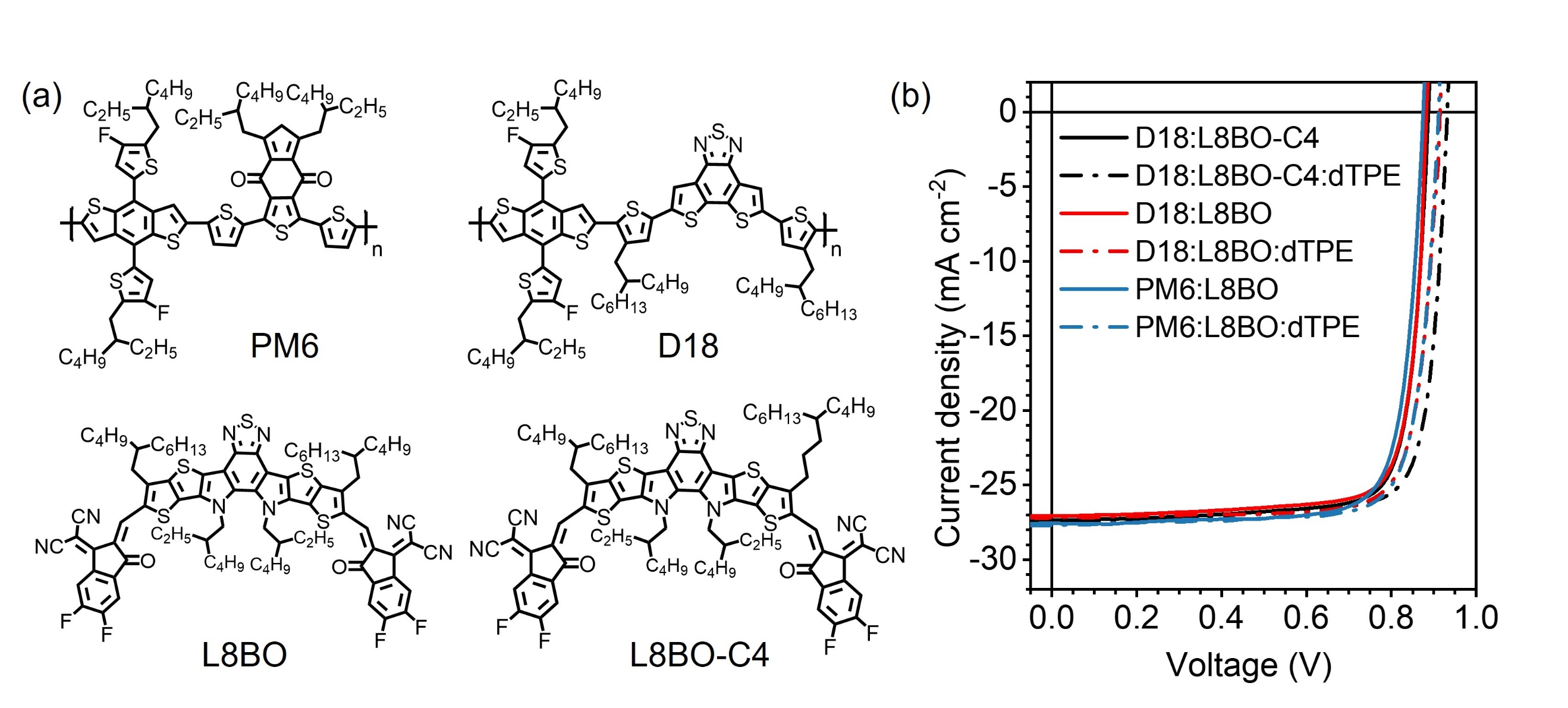
**

**Figure S33.** (a) The chemical structures of PM6, D18, L8BO, and L8BO-C4. (b) The characteristic *J*-*V* curves of different binary and ternary devices.

1. **Impedance analysis**

**
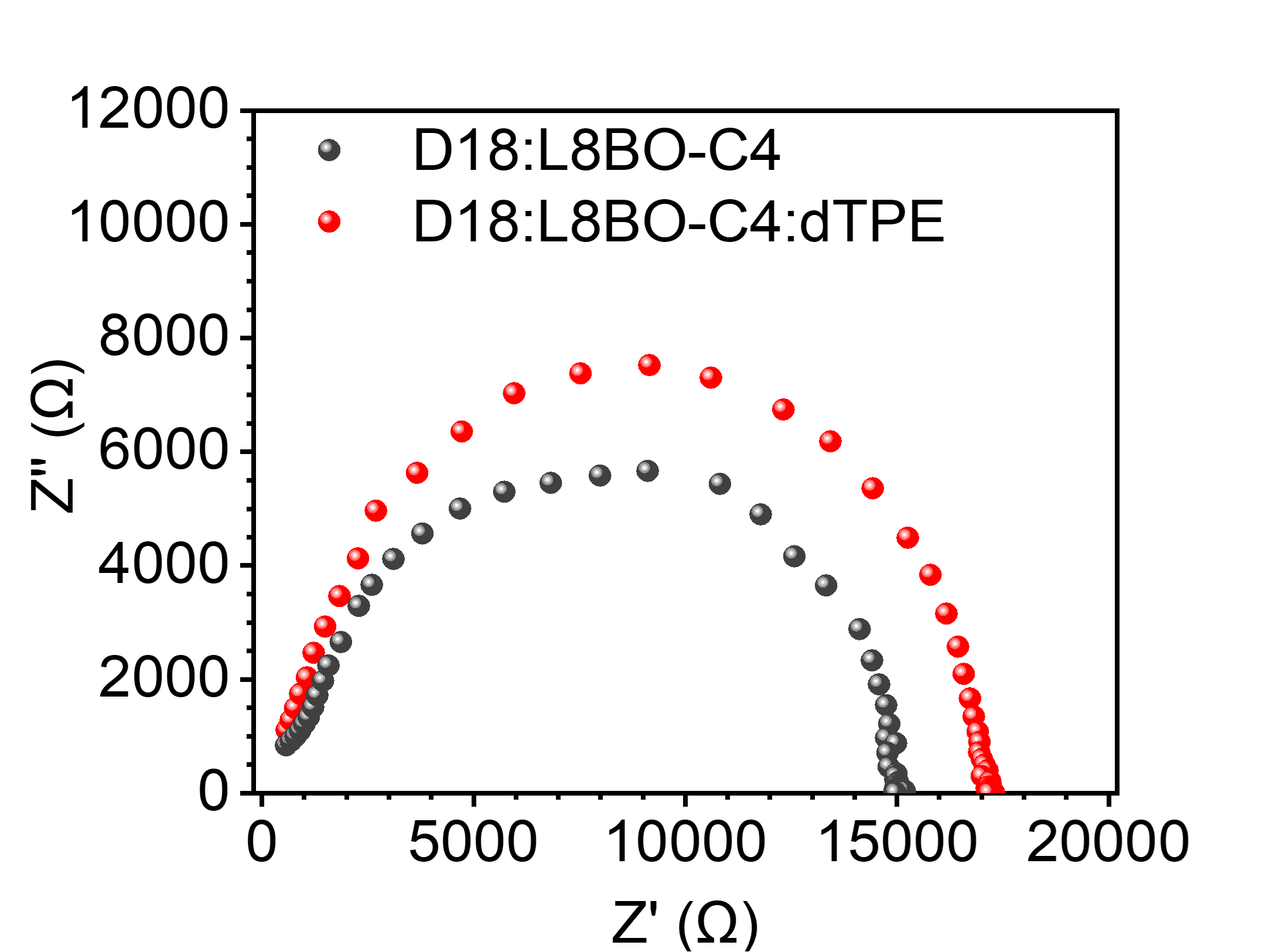
**

**Figure S34.** Nyquist plots of the OSC devices with D18:L8BO-C4 and D18:L8BO-C4:dTPE active layer under dark conditions.

**Supplementary Tables**

**Table S1.** Detailed photovoltaic parameters of the ternary OPV devices with different third content under AM 1.5 G illumination (100 mW cm^−2^).

| Active layer | Blend Ratio | *V*_OC_ [V] | *J*_SC_ [mA cm^-2^] | FF [%] | PCE [%] |
| --- | --- | --- | --- | --- | --- |
| D18:L8BO-C4:dTPE | 1:1.2:0 | 0.886 | 27.33 | 80.23 | 19.37 |
|  | 1:1.1:0.1 | 0.908 | 27.55 | 80.29 | 20.12 |
|  | 1:1:0.2 | 0.929 | 27.51 | 80.18 | 20.51 |
|  | 1:0.9:0.3 | 0.944 | 27.21 | 79.16 | 20.29 |
|  | 1:0.8:0.4 | 0.958 | 26.59 | 76.73 | 19.46 |
|  | 1:0.6:0.6 | 0.979 | 24.32 | 71.63 | 17.09 |
|  | 1:0:1.2 | 1.069 | 13.69 | 56.38 | 8.21 |
|  | 1:1.2:0.1 | 0.902 | 27.49 | 80.25 | 19.91 |
|  | 1:1.2:0.05 | 0.890 | 27.37 | 80.19 | 19.57 |

**Table S2.** The detailed photovoltaic parameters and hole/electron mobilities of binary and ternary blend films

| Active layer | *J*_ph_  [mA cm^-2^] | *J*_sat_  [mA cm^-2^] | *η*  [%] | *J*_d,-2V_  [A cm^-2^] | *μ*_e_ × 10^4^  [cm^2^ V^-1^ s^-1^] | *μ*_h_ × 10^4^  [cm^2^ V^-1^ s^-1^] | *μ*_h_*/μ_e_* |
| --- | --- | --- | --- | --- | --- | --- | --- |
| D18: L8BO-C4 | 27.44 | 27.67 | 99.0 | 2.67 × 10^-4^ | 4.66 ± 0.19 | 6.49 ± 0.17 | 1.39 |
| D18: dTPE | 12.35 | 14.96 | 82.5 | 3.26 × 10^-6^ | 3.14 ± 0.12 | 7.64 ± 0.29 | 2.43 |
| D18: dSpiro | - | - | - | - | 1.83 ± 0.20 | 8.45 ± 0.31 | 4.62 |
| D18: L8BO-C4: dTPE (1:1.1:0.1) | - | - | - | - | 5.58 ± 0.30 | 7.09 ± 0.11 | 1.27 |
| D18: L8BO-C4: dTPE(1:1:0.2) | 27.69 | 27.86 | 99.3 | 1.89 × 10^-5^ | 5.67 ± 0.25 | 7.55 ± 0.23 | 1.33 |

**Table S3.** Basic optical and electrical properties of L8BO-C4, dTPE, and dSpiro.

| Material | λ_sol_  [nm] | λ_film_  [nm] | *ε*  [L mol^−1^ cm^−1^] | *E*_g_  [eV] | *E*_HOMO_  [eV] | *E*_LUMO_  [eV] | PLQY  [%] | *μ*_e_  [×10^−4^ cm^2^ V^−1^ s^−1^] |
| --- | --- | --- | --- | --- | --- | --- | --- | --- |
| L8BO-C4 | 728 | 802 | 1.76 × 10^5^ | 1.40 | -5.64 | -3.89 | 1.33 ± 0.13 | 9.67 ± 0.26 |
| dTPE | 727 | 779 | 1.51 × 10^5^ | 1.46 | -5.56 | -3.83 | 4.69 ± 0.19 | 5.28 ± 0.19 |
| dSpiro | 728 | 781 | 1.23 × 10^5^ | 1.44 | -5.47 | -3.77 | 2.43 ± 0.21 | 2.87 ± 0.17 |

**Table S4.** The detailed GIWAXS characterization data of pure films.

| Film | Direction | (100) |  |  |  | (010) |  |  |  |
| --- | --- | --- | --- | --- | --- | --- | --- | --- | --- |
|  |  | Location | d-spacing | FWHM | CL | Location | d-spacing | FWHM | CL |
|  |  | [Å^-1^] | [Å] | [Å^-1^] | [Å] | [Å^-1^] | [Å] | [Å^-1^] | [Å] |
| D18 | in-plane | 0.30 | 20.53 | 0.081 | 70 | 1.70 | 3.69 | 0.172 | 33 |
|  | out-of-plane | 0.33 | 19.27 | 0.066 | 86 | 1.72 | 3.65 | 0.240 | 24 |
| L8BO-C4 | in-plane | 0.36 | 17.30 | 0.127 | 45 |  |  |  |  |
|  | out-of-plane |  |  |  |  | 1.76 | 3.56 | 0.159 | 36 |
| dTPE | in-plane | 0.43 | 14.57 | 0.140 | 41 |  |  |  |  |
|  | out-of-plane |  |  |  |  | 1.62 | 3.86 | 0.183 | 31 |
| dSpiro | in-plane | 0.48 | 13.06 | 0.219 | 25 |  |  |  |  |
|  | out-of-plane |  |  |  |  | 1.61 | 3.89 | 0.342 | 17 |

**Table S5.** The detailed GIWAXS characterization data of blend films.

| Film | Direction | (100) |  |  |  | (010) |  |  |  |
| --- | --- | --- | --- | --- | --- | --- | --- | --- | --- |
|  |  | Location | d-spacing | FWHM | CL | Location | d-spacing | FWHM | CL |
|  |  | [Å^-1^] | [Å] | [Å^-1^] | [Å] | [Å^-1^] | [Å] | [Å^-1^] | [Å] |
| D18: | in-plane | 0.31 | 20.46 | 0.060 | 94 |  |  |  |  |
| L8BO-C4 | out-of-plane |  |  |  |  | 1.73 | 3.62 | 0.214 | 26 |
| D18: | in-plane | 0.31 | 20.43 | 0.063 | 90 |  |  |  |  |
| dTPE | out-of-plane |  |  |  |  | 1.71 | 3.66 | 0.239 | 23 |
| D18: | in-plane | 0.30 | 20.73 | 0.069 | 81 |  |  |  |  |
| dSpiro | out-of-plane |  |  |  |  | 1.69 | 3.71 | 0.265 | 20 |
| D18:L8BO- | in-plane | 0.31 | 20.38 | 0.057 | 100 |  |  |  |  |
| C4: dTPE | out-of-plane |  |  |  |  | 1.75 | 3.58 | 0.198 | 29 |

**Table S6.** Summary of the energy loss analysis of the state-of-art organic solar cells in the last year.

| Cited Ref. | *E_loss_* [eV] | Δ*E*_3_ [eV] | *EQE*_EL_ |
| --- | --- | --- | --- |
| *J. Am. Chem. Soc.* **2025**, *147*, 24491−24501[^9^](#_ENREF_9) | 0.521 | 0.213 | 2.67×10^-4^ |
|  | 0.498 | 0.192 | 5.88×10^-4^ |
|  | 0.491 | 0.189 | 6.63×10^-4^ |
| *Energy Environ. Sci.*, **2025**, *18*, 5378-5388[^10^](#_ENREF_10) | 0.613 | 0.224 | 1.72×10^-4^ |
|  | 0.608 | 0.243 | 8.38×10^-5^ |
|  | 0.578 | 0.211 | 2.80×10^-4^ |
| *Energy Environ. Sci.*, **2025**, *18*, 6214-6223[^11^](#_ENREF_11) | 0.592 | 0.218 | 2.15×10^-4^ |
|  | 0.571 | 0.196 | 5.02×10^-4^ |
|  | 0.545 | 0.137 | 4.11×10^-3^ |
| *Nat. Mater.* **2025**, *24*, 433-443[^1^](#_ENREF_1) | 0.549 | 0.224 | 1.67×10^-4^ |
|  | 0.557 | 0.209 | 1.65×10^-4^ |
|  | 0.543 | 0.210 | 2.90×10^-4^ |
| *J. Am. Chem. Soc.* **2025**, *147*, 21241–21251[^12^](#_ENREF_12) | 0.551 | 0.245 | - |
|  | 0.528 | 0.223 | - |
| *Adv. Mater.* **2025,** *37*, 2413376[^13^](#_ENREF_13) | 0.491 | 0.143 | 3.54×10^-3^ |
|  | 0.476 | 0.171 | 1.14×10^-3^ |
| *Angew. Chem. Int. Ed.* **2025**, e202416883[^14^](#_ENREF_14) | 0.537 | 0.225 | 1.62×10^-4^ |
|  | 0.476 | 0.195 | 5.60×10^-4^ |
|  | 0.471 | 0.165 | 1.64×10^-3^ |
| *Nat. Commun.* **2025**, *16*, 1784[^15^](#_ENREF_15) | 0.556 | 0.227 | 2.01×10^-4^ |
|  | 0.534 | 0.207 | 1.64×10^-4^ |
| *Energy Environ. Sci.*, **2025**, *18*, 2536–2545[^16^](#_ENREF_16) | 0.527 | 0.229 | 1.83×10^-4^ |
|  | 0.531 | 0.233 | 1.27×10^-4^ |
| *Adv. Mater.* **2025**, *37*, 2500282[^17^](#_ENREF_17) | 0.533 | 0.194 | 4.63×10^-4^ |
|  | 0.526 | 0.195 | 4.41×10^-4^ |
| This work | 0.516 | 0.190 | 5.39×10^-4^ |
|  | 0.452 | 0.130 | 5.68×10^-3^ |

**Table S7.** Summary of the PCE and *V*_OC_ of the state-of-art ternary organic solar cells reported in last year.

| Cited | Active layer | *V*_OC_ [V] | PCE [%] |
| --- | --- | --- | --- |
| *Energy Environ. Sci.*, **2025**, *18,* 6214-6223[^11^](#_ENREF_11) | PM6:L8BO:Z-Tri | 0.927 | 20.32 |
| *Nat. Mater.* **2025**, *24*, 433-443[^1^](#_ENREF_1) | PM6:L8-BO-C4:L8-BO-C4-Br | 0.889 | 20.10 |
| *Nat. Energy*, **2024**, *9*, 975–986[^18^](#_ENREF_18) | D18:Z8:L8-BO | 0.900 | 19.80 |
| *Mat. Sci. & Eng. R*, **2026**, *167*, 101118[^19^](#_ENREF_19) | PM6:BTP-eC9:Pz-E2F | 0.862 | 19.43 |
|  | PM6:BTP-eC9:Pz-E2Cl | 0.853 | 18.82 |
|  | PM6:BTP-eC9:Pz-E2F | 0.860 | 20.03 |
| *J. Mater. Chem. C,* **2025**  DOI: 10.1039/d5tc03087k[^20^](#_ENREF_20) | PM6:BTA-E3:Cl24-H | 0.861 | 20.21 |
| *Energy Environ. Sci.,* **2025**  DOI: 10.1039/d5ee04322k[^21^](#_ENREF_21) | D18:N3:QX-Cl | 0.847 | 20.41 |
|  | PM6:BTP-eC9:NA6 | 0.872 | 19.40 |
|  | PM6:BTP-eC9:NA7 | 0.883 | 20.14 |
| *ACS Appl. Energy Mater.* **2025**, *18*, 13741–13751[^22^](#_ENREF_22) | PM6:Y6:IDT-T-PCN | 0.859 | 17.36 |
| *Adv. Funct. Mater*. **2025**, e16196[^23^](#_ENREF_23) | PM6:L8-BO-C4: L8-BO-C4-Br | 0.896 | 19.64 |
|  | PM6:L8-BO | 0.888 | 19.16 |
| *Adv. Mater.* **2025**, e10980[^24^](#_ENREF_24) | LWPM6:Y6:TYT-S | 0.850 | 19.23 |
|  | HWPM6:Y6:TYT-S | 0.851 | 19.12 |
| *Energy Environ. Sci.*, **2025**, *18*, 6608-6617[^25^](#_ENREF_25) | PM6:L8-BO:BQx-O | 0.896 | 19.0 |
|  | D18:L8-BO:BQx-O | 0.930 | 19.91 |
| *Joule*, **2024**, *8*, 2304-2324[^26^](#_ENREF_26) | PM6:PY-V-g: PffBQx-T | 0.915 | 18.6 |
| *Energy Environ. Sci.*, **2025**, *18*, 7071-7081[^27^](#_ENREF_27) | D18:L8-BO:PY-DT | 0.917 | 20.0 |
| *Adv. Mater.* **2025**, *37*, 2503702[^28^](#_ENREF_28) | P[4,8]BBO:PM6:PY-IT | 0.89 | 19.1 |
| *J. Mater. Chem. A*, **2025**, *13*, 22163-22170[^29^](#_ENREF_29) | PM6 : BTP-4Cl : S-EDOT | 0.882 | 18.29 |
| *Chem. Eng. J.* **2025**, *516*, 164193[^30^](#_ENREF_30) | PM6:L8-BO: P-ICB3F | 0.89 | 18.87 |
| *J. Mater. Chem. C,* **2025**, *13*, 13768[^31^](#_ENREF_31) | BTR-Cl:Y6:BO-4I | 0.846 | 16.26 |
| *Small* **2025**, *21*, 2503200[^32^](#_ENREF_32) | D18:L8-BO:WLA1 | 0.913 | 18.52 |
|  | D18:L8-BO:WLA2 | 0.911 | 19.64 |
| This work | D18:L8BO-C4:dTPE (1:1:0.2) | 0.929 | 20.51 |
|  | D18:L8BO-C4:dTPE (1:0.9:0.3) | 0.944 | 20.29 |
|  | D18:L8BO-C4:dTPE (1:0.8:0.4) | 0.958 | 19.46 |

**Table S8.** Summary of the photovoltaic parameters of binary and ternary devices under under continuous simulated solar illumination.

| Active layer | Storing time [h] | V_OC_ [V] | *J*_SC_ [mA cm^-2^] | FF [%] | PCE [%] |
| --- | --- | --- | --- | --- | --- |
| D18:L8BO-C4 | 0 | 0.885 | 27.12 | 79.94 | 19.12 |
|  | 4 | 0.886 | 26.75 | 80.03 | 18.91 |
|  | 24 | 0.885 | 26.05 | 79.73 | 18.38 |
|  | 48 | 0.884 | 25.81 | 79.60 | 18.15 |
|  | 72 | 0.883 | 25.07 | 79.33 | 17.64 |
|  | 96 | 0.883 | 24.79 | 79.06 | 17.43 |
|  | 144 | 0.883 | 24.53 | 78.89 | 17.02 |
|  | 192 | 0.882 | 24.10 | 78.59 | 16.71 |
|  | 240 | 0.882 | 23.92 | 78.26 | 16.50 |
| D18:L8BO-C4:dTPE | 0 | 0.927 | 27.40 | 80.10 | 20.32 |
|  | 4 | 0.928 | 27.43 | 80.03 | 20.40 |
|  | 24 | 0.928 | 27.22 | 79.82 | 20.11 |
|  | 48 | 0.926 | 26.55 | 79.51 | 19.55 |
|  | 72 | 0.925 | 26.32 | 79.60 | 19.38 |
|  | 96 | 0.924 | 25.78 | 79.43 | 18.91 |
|  | 144 | 0.924 | 25.21 | 79.51 | 18.54 |
|  | 192 | 0.923 | 24.98 | 79.27 | 18.20 |
|  | 240 | 0.922 | 24.76 | 79.19 | 18.07 |

**Table S9.** Summary of the photovoltaic parameters of binary and ternary devices under under 80 ^o^C continuous annealing in glove box.

| Active layer | Annealing time [h] | V_OC_ [V] | *J*_SC_ [mA cm^-2^] | FF [%] | PCE [%] |
| --- | --- | --- | --- | --- | --- |
| D18:L8BO-C4 | 0 | 0.886 | 26.76 | 80.24 | 19.02 |
|  | 2 | 0.886 | 26.28 | 80.02 | 18.63 |
|  | 4 | 0.886 | 26.37 | 79.88 | 18.66 |
|  | 10 | 0.885 | 25.92 | 79.53 | 18.25 |
|  | 24 | 0.885 | 25.36 | 79.11 | 17.76 |
|  | 48 | 0.885 | 24.93 | 78.66 | 17.36 |
|  | 72 | 0.884 | 24.37 | 77.62 | 16.74 |
|  | 144 | 0.884 | 23.53 | 77.03 | 16.01 |
|  | 240 | 0.882 | 23.07 | 75.98 | 15.39 |
| D18:L8BO-C4:dTPE | 0 | 0.928 | 27.21 | 80.05 | 20.25 |
|  | 2 | 0.927 | 26.69 | 80.13 | 19.81 |
|  | 4 | 0.927 | 26.92 | 79.62 | 19.90 |
|  | 10 | 0.926 | 26.43 | 79.76 | 19.56 |
|  | 24 | 0.926 | 25.88 | 79.29 | 19.02 |
|  | 48 | 0.924 | 25.60 | 78.87 | 18.68 |
|  | 72 | 0.925 | 25.27 | 78.13 | 18.28 |
|  | 144 | 0.924 | 24.39 | 77.72 | 17.50 |
|  | 240 | 0.923 | 23.96 | 77.31 | 17.12 |

**Table S10.** Summary of the photovoltaic parameters of different binary and ternary devices.

| Active layer | Blend Ratio | V_OC_ [V] | *J*_SC_ [mA cm^-2^] | FF [%] | PCE [%] |
| --- | --- | --- | --- | --- | --- |
| D18:L8BO-C4 | 1:1.2 | 0.886 | 27.33 | 80.23 | 19.37 |
| D18:L8BO-C4:dTPE | 1:1:0.2 | 0.929 | 27.51 | 80.18 | 20.51 |
| PM6:L8BO | 1:1.2 | 0.875 | 27.58 | 79.71 | 19.22 |
| PM6:L8BO:dTPE | 1:1:0.2 | 0.912 | 27.71 | 79.53 | 20.09 |
| D18:L8BO | 1:1.2 | 0.882 | 27.06 | 80.13 | 19.10 |
| D18:L8BO:dTPE | 1:1:0.2 | 0.914 | 27.17 | 79.91 | 19.84 |

**References**

[1] C. Li; J. Song; H. Lai; H. Zhang; R. Zhou; J. Xu; H. Huang; L. Liu; J. Gao; Y. Li; M. H. Jee; Z. Zheng; S. Liu; J. Yan; X. K. Chen; Z. Tang; C. Zhang; H. Y. Woo; F. He; F. Gao; H. Yan; Y. Sun. *Nat Mater* **2025**, *24*, 433-443.

[2] B. Hess; C. Kutzner; D. van der Spoel; E. Lindahl. *J. Chem. Theory Comput.* **2008**, *4*, 435-447.

[3] V. Coropceanu; X.-K. Chen; T. Wang; Z. Zheng; J.-L. Brédas. *Nat. Rev. Mater.* **2019**, *4*, 689-707.

[4] S. Pronk; S. Páll; R. Schulz; P. Larsson; P. Bjelkmar; R. Apostolov; M. R. Shirts; J. C. Smith; P. M. Kasson; D. van der Spoel; B. Hess; E. Lindahl. *Bioinformatics* **2013**, *29*, 845-854.

[5] J. Wang; R. M. Wolf; J. W. Caldwell; P. A. Kollman; D. A. Case. *J Comput Chem* **2004**, *25*, 1157-1174.

[6] C. I. Bayly; P. Cieplak; W. Cornell; P. A. Kollman. *The Journal of Physical Chemistry* **1993**, *97*, 10269-10280.

[7] M. Ghasemi; N. Balar; Z. Peng; H. Hu; Y. Qin; T. Kim; J. J. Rech; M. Bidwell; W. Mask; I. McCulloch; W. You; A. Amassian; C. Risko; B. T. O'Connor; H. Ade. *Nat Mater* **2021**, *20*, 525-532.

[8] H. Hu; M. Ghasemi; Z. Peng; J. Zhang; J. J. Rech; W. You; H. Yan; H. Ade. *Adv Mater* **2020**, *32*, e2005348.

[9] J. Zhu; R. Zeng; E. Zhou; C. Li; J. Deng; M. Du; Q. Guo; M. Ji; Z. Wang; Y. Lin; F. Han; J. Zhuang; S. Tan; L. Kan; L. Zhu; M. Zhang; F. Liu. *J. Am. Chem. Soc.* **2025**, *147*, 24491-24501.

[10] J. Zhang; X. Duan; X. Li; G. Dai; J. Deng; X. Wang; J. Qiao; H. Wu; L. Liu; H. Huang; S. Liu; J. Yan; H. Zhang; X.-T. Hao; R. Yang; F. Gao; Y. Sun. *Energy Environ. Sci.* **2025**, *18*, 5378-5388.

[11] Y. Chen; X. Duan; J. Zhang; Z. Ge; H. Ma; X. Sun; H. Zhang; J. Gao; X. Wang; X. Wang; Z. Tang; R. Yang; F. Gao; Y. Sun. *Energy Environ. Sci.* **2025**, *18*, 6214-6223.

[12] H. Mou; Y. Yin; H. Chen; J. Xu; J. Ding; C. Ju; J. Zhu; Y. Wang; W. Chen; G. Xu; T. Zhang; J. Li; Y. Li; Y. Li. *J Am Chem Soc* **2025**, *147*, 21241-21251.

[13] K. Liu; Y. Jiang; F. Liu; G. Ran; M. Wang; W. Wang; W. Zhang; Z. Wei; J. Hou; X. Zhu. *Adv Mater* **2025**, *37*, e2413376.

[14] Q. Jiang; X. Yuan; Y. Li; Y. Luo; J. Zhu; F. Zhao; Y. Zhang; W. Wei; H. Feng; H. Li; J. Wu; Z. Ma; Z. Tang; F. Huang; Y. Cao; C. Duan. *Angew. Chem. Int. Ed.* **2025**, *64*, e202416883.

[15] J. Wang; C. Sun; Y. Li; F. Bi; H. Jiang; C. Yang; X. Bao; J. Chu. *Nat Commun* **2025**, *16*, 1784.

[16] X. Sun; F. Wang; G. Yang; X. Ding; J. Lv; Y. Sun; T. Wang; C. Gao; G. Zhang; W. Liu; X. Xu; S. Satapathi; X. Ouyang; A. Ng; L. Ye; M. Yuan; H. Zhang; H. Hu. *Energy Environ. Sci.* **2025**, *18*, 2536-2545.

[17] Y. Jiang; K. Liu; F. Liu; G. Ran; M. Wang; T. Zhang; R. Xu; H. Liu; W. Zhang; Z. Wei; Y. Cui; X. Lu; J. Hou; X. Zhu. *Adv Mater* **2025**, *37*, e2500282.

[18] Y. Jiang; S. Sun; R. Xu; F. Liu; X. Miao; G. Ran; K. Liu; Y. Yi; W. Zhang; X. Zhu. *Nat. Energy* **2024**, *9*, 975-986.

[19] G. Tian; Y. Chen; Y. Li; L. Liu; Q. Ma; S. Duan; C. Uragami; H. Hashimoto; P. Huang; C. Yang; Y. Yang; S. Lu; Z. Xiao. *Materials Science and Engineering: R: Reports* **2026**, *167*, 101118.

[20] Y. Li; Y. Gong; X. Li; H. He; S. Qin; J. Zhang; J. Zhang; F. Pan; L. Meng; Y. Li. *J. Mater. Chem. C* **2025**, DOI:10.1039/d1035tc03087k.

[21] K. Hu; Y. Ge; H. Yang; Y. Xu; J. Qian; X. Zhu; Y. Wu; C. Cui; Y. Li. *Energy Environ. Sci.* **2025**, DOI: 10.1039/d1035ee04322k.

[22] X. Tang; X. Peng; H. Liao; X. Zeng; X. Zhang; Y. Zhu; C. Weng; P. Shen. *ACS Appl. Energy Mater.* **2025**, *8*, 13741-13751.

[23] G. Chen; H. Huang; W. Ma; J. Song; L. Guo; C. Li; L. Wang; Z. Qin; M. Zhang; Y. Liu; B. He; C. Dai; Z. He; H. Chen; Y. Sun; F. Liu; J. Yan; S. Liu. *Adv. Funct. Mater.* **2025**, e16196.

[24] Y. Yang; L. Wei; L. Zhan; Y. Liu; H. Lu; X. Wu; A. Wupur; T. Chen; J. Yu; X. Sun; H. Hu; R. Sun; J. Min; Y. Luo; J. Wu; W. Fu; S. Yin; H. Chen. *Adv Mater* **2025**, e10980.

[25] L. Chen; W. Liang; A. Sergeev; J. Y. L. Lai; X. Zeng; K. S. Wong; J. Zhang; S. H. Pun; H. Yan; H. Hu. *Energy Environ. Sci.* **2025**, *18*, 6608-6617.

[26] H. Yu; Y. Wang; C. H. Kwok; R. Zhou; Z. Yao; S. Mukherjee; A. Sergeev; H. Hu; Y. Fu; H. M. Ng; L. Chen; D. Zhang; D. Zhao; Z. Zheng; X. Lu; H. Yin; K. S. Wong; H. Ade; C. Zhang; Z. Zhu; H. Yan. *Joule* **2024**, *8*, 2304-2324.

[27] F. Sun; J. Wu; B. Cheng; L. Kan; F. Hua; W. Sun; H. Wang; Y. Huo; S. Chen; X. Xia; X. Du; F. Liu; E. Wang; X. Guo; Y. Li; M. Zhang. *Energy Environ. Sci.* **2025**, *18*, 7071-7081.

[28] M. Liu; L. Wu; Y. Hai; Y. Luo; Y. Li; R. Chen; Y. Ma; T. Jia; Q. Li; S. Liu; R. Ma; Y. P. Cai; J. Wu; G. Li; S. Liu. *Adv. Mater.* **2025**, *37*, 2503702.

[29] B. Zhou; B. Shao; B. Fan; W. Chen; Q. Shi; Y. Nai; H. Yang; J. Yuan; Y. Zou. *J. Mater. Chem. A* **2025**, *13*, 22163-22170.

[30] Y. Zhai; Y. Shi; K. Yang; R. Fang; H. Bi; J. Zhang; Y. Chang; A. Tang; Z. Wei; K. Lu. *Chem. Eng. J.* **2025**, *516*, 164193.

[31] W. Zhou; J. Wang; W. Shi; J. Wang; S. Yuan; B. Lan; B. Kan. *J. Mater. Chem. C* **2025**, *13*, 13768-13775.

[32] Z. Wei; Y. Liu; Y. Wang; G. Ran; H. Li; Y. Cheng; H. Lu; X. Yan; C. Zhang; S. Song; Y. Shu; Y. Liu; W. Zhang; Z. Bo. *Small* **2025**, *21*, 2503200.
